# Targeting PTDSS1 to modulate GSH synthesis triggers mitophagy and induces ferroptosis in esophageal squamous cell carcinoma cells

**DOI:** 10.1038/s41419-026-08702-4

**Published:** 2026-04-23

**Authors:** Xiao Zhang, Shurui Cao, Chenchen Zhou, Wen Jiang, Hongshun Wang, Bingqing Hui, Xiaheng Deng, Yanhong Gu

**Affiliations:** 1 Department of Oncology and Cancer Rehabilitation Center, The First Affiliated Hospital of Nanjing Medical University, Nanjing, Jiangsu China; 2 The First Clinical Medical College of Nanjing Medical University, Nanjing, Jiangsu China; 3 Jiangsu Provincial Key Laboratory of Chronic Digestive Diseases, The First Affiliated Hospital with Nanjing Medical University, Nanjing, China; 4 Department of Thoracic Surgery, The First Affiliated Hospital of Nanjing Medical University, Nanjing, China

**Keywords:** Oesophageal cancer, Translational research

## Abstract

PTDSS1 is an emerging oncogenic protein associated with poor survival rates across various cancer types, including esophageal squamous cell carcinoma (ESCC). However, its regulatory mechanisms and therapeutic potential in ESCC remain incompletely understood. Through single-cell RNA sequencing (scRNA-seq) analysis, we identified a PTDSS1-high malignant epithelial subpopulation characterized by resistance to ferroptosis and mitophagy. Our investigations demonstrated that PTDSS1 regulates glutathione (GSH) synthesis and coordinates mitophagy in ESCC cells. Mechanistically, PTDSS1 knockdown promotes interaction between TRIM21 and SLC3A2, leading to diminished SLC3A2 protein expression and subsequent reduction in GSH synthesis. This elevates cellular oxidative stress, thereby triggering PINK1/Parkin mitophagy pathway and ultimately inducing apoptosis and ferroptosis. Furthermore, at the mitochondrial level, the knockdown of PTDSS1 decreases phosphatidylserine (PS) and facilitates mitochondrial fusion protein 2 (MFN2) translocation, providing substrates for mitophagy. Collectively, our findings elucidate a novel mechanism by which PTDSS1 protects ESCC cells from death and offer new perspectives for therapeutic strategies that target PTDSS1 to induce mitophagy and ferroptosis in ESCC.

## Introduction

Esophageal cancer represents the sixth leading cause of cancer-related mortality globally, with esophageal squamous cell carcinoma (ESCC) accounting for approximately 90% of cases. ESCC is characterized by late-stage diagnosis, aggressive metastasis, therapeutic resistance, and high recurrence rates [1, 2]. Endoscopic therapy, surgery and chemoradiotherapy remain the main treatment approaches for ESCC, with immunotherapy emerging as a valuable addition since 2018. Combining immunotherapy with neoadjuvant chemoradiotherapy has shown promise in maximizing regression of locally advanced esophageal cancer [3, 4]. Despite these advances, intrinsic or acquired resistance to immunotherapy remains a significant obstacle to improving patient outcomes. Targeted therapies against EGFR, FGFR, PI3K, and CDK4/6 have shown initial efficacy [5]. however, the substantial intratumoral heterogeneity of ESCC has limited the impact of many molecular targeted therapies on patient prognosis. Therefore, there is an urgent clinical need to identify novel biomarkers and develop innovative treatment strategies to enhance treatment efficacy and improve long-term outcomes in ESCC patients.

Ferroptosis was first precisely characterized as a distinct form of regulated cell death by Brent R. Stockwell and colleagues in 2012 [6]. The core mechanism resides in the abnormal accumulation of iron -dependent lipid peroxides (LPOs), exerting an inhibitory effect on the progression of malignant tumors [7, 8]. Most studies have disclosed a mechanistic correlation between mitochondrial dysfunction and ferroptosis, regulating the ferroptosis process of tumor cells in a mitochondria-dependent manner [9, 10]. The forefront research advancements reveal that the development of novel anti-tumor therapeutic strategies, based on the synergistic regulatory mechanism of mitophagy and ferroptosis and targeting the activation of the mitophagy pathway and the induction of ferroptosis, exhibits remarkable therapeutic potential in overcoming tumor drug resistance and inhibiting metastasis [11–14].

In this study, we identified a PTDSS1-high malignant epithelial subpopulation through single-cell RNA sequencing (scRNA-seq) analysis, revealing its association with ferroptosis and mitophagy resistance. PTDSS1 encodes phosphatidylserine synthase, an enzyme responsible for the synthesis of phosphatidylserine (PS) in the endoplasmic reticulum (ER). PS is then transported to the plasma membrane (PM) and mitochondria through distinct pathways [15, 16]. Previous studies have shown that reducing PS levels in mitochondria can inhibit mitochondrial protein import and disrupt mitochondrial morphology, leading to mitochondrial dysfunction. Interestingly, reducing the transport of PS from ER to PM can partially rescue the mitochondrial defects caused by the loss of PTDSS1 function [17–19]. Mitochondrial dysfunction and aberrant energy metabolism represent major hallmarks of cancer that can activate mitophagy, potentially promoting tumor progression [20, 21]. However, the role of PTDSS1 in regulating mitochondrial structure and function in ESCC, and its relationship with mitophagy, remains poorly defined.

Mitophagy represents a selective autophagic process capable of eliminating damaged mitochondria and reduces reactive oxygen species (ROS) production [22]. thereby maintaining mitochondrial homeostasis. Two primary pathways mediate mitophagy: the ubiquitin-dependent route mediated by PINK1-Parkin, and the ubiquitin-independent course mediated by receptor proteins such as BNIP3, NIX, and FUNDC1 [23]. Regarding the interplay between mitophagy and cancer, the precise role of mitophagy remains a subject of ongoing debate. On one hand, mitophagy can facilitate cancer cell survival by degrading damaged mitochondria and reducing the production of mitochondrial ROS (mtROS); on the other hand, oxidative stress or prolonged mitochondrial damage can lead to pathological overactivation of mitophagy, potentially inducing ferroptosis and apoptosis [22, 24, 25]. Inducing excessive mitophagy to promote ferroptosis in cancer cells has emerged as a promising anti-tumor therapeutic strategy [24, 26, 27].

In this study, we found that in ESCC, targeted suppression of the expression of PTDSS1 facilitates the combination of SLC3A2 and TRIM21 in PM, inhibiting glutathione (GSH) generation and inducing oxidative stress in tumor cells. Additionally, the reduction of PS levels promotes the transport of mitochondrial fusion protein 2 (MFN2) to the outer mitochondrial membrane, where it is specifically recognized and bound by PINK1. Subsequently, MFN2 is preferentially ubiquitinated by Parkin on mitochondria following PINK1 recruitment, activating mitophagy. Oxidative stress accelerates the PINK1/Parkin/MFN2-mediated mitophagy pathway, thereby inducing ferroptosis and apoptosis in tumor cells and inhibiting tumor progression. This discovery may help develop selective treatment strategies for ESCC patients to prevent cancer progression and metastasis.

## Material and methods

### Data acquisition and preprocessing

Single-cell RNA sequencing (scRNA-seq) datasets from three publicly available Gene Expression Omnibus (GEO) repositories (accession codes: GSE160269, GSE188990, GSE197677) were processed for analysis. Raw gene expression matrices were subjected to stringent quality control using the Scanpy Python package (v1.10.3). Cells with <200 detected genes, <250 unique molecular identifiers (UMIs), or >12% mitochondrial gene content to ensure data quality. A total of 59,280 malignant cells were retained for downstream analysis. Batch effects across datasets were corrected using the bbknn algorithm (v1.6.0) integrated into Scanpy.

### Cell clustering and annotation

Normalized data were log-transformed, and principal component analysis (PCA) was performed to reduce dimensionality using the first 30 principal components. Graph-based clustering was applied using the Louvain algorithm with a resolution of 0.5 to identify major cell populations. For cell type annotation, we employed a comprehensive marker-based approach using established lineage-specific markers: epithelial cells (EPCAM, KRT8, KRT18, KRT19, CDH1); cancer cells (MKI67, BIRC5, TOP2A, UBE2C, CCNB1); T cells (CD3D, CD3E, CD8A, CD4, CCR7); B cells (CD79A, CD79B, MS4A1, CD19, CD20); macrophages (CD68, CD163, MSR1, FCGR1A, C1QA); fibroblasts (COL1A1, COL3A1, DCN, LUM, FAP); endothelial cells (PECAM1, VWF, CLDN5, ENG, KDR); mast cells (TPSAB1, TPSB2, CPA3, MS4A2, HPGDS); NK cells (NCAM1, NKG7, KLRD1, KLRF1, GNLY); and dendritic cells (CD1C, CLEC9A, CLEC10A, LAMP3, IDO1).

We implemented CellTypist (v1.1.0) with “Immune_All” and “Epithelial” reference models to assign probabilistic cell type labels, using a prediction score threshold of 0.7. The CancerFinder-scf algorithm (v2.0) was applied to distinguish malignant from non-malignant cells based on CNV inference and transcriptional dysregulation scores, with a malignancy score threshold of 0.75. Cells classified as malignant by both methods were retained for downstream analysis (*n* = 59,280).

### Subpopulation characterization and pathway analysis

Unsupervised clustering of the identified cancer cells was performed using the leiden algorithm at a resolution of 0.5, revealing five distinct subpopulations with unique transcriptional profiles. For functional characterization of these clusters, we assembled curated gene sets for key cancer-related pathways, including ferroptosis (*n* = 137 genes, based on the ferroptosis signatures from FerrDb and MSigDB hallmark collection) and mitophagy (*n* = 84 genes, based on the PINK1/PARKIN signaling pathway from Reactome and GO:0000422 terms). Pathway activity was quantified using a modified Z-score method, where for each pathway and each cell, we calculated the average expression of pathway genes after subtracting the cell-specific mean expression and dividing by the standard deviation of expression across all genes in that cell. This controlled for cell-specific technical variations in gene detection sensitivity.

To determine statistical significance of pathway enrichment across clusters, we employed the Wilcoxon rank-sum test with Bonferroni correction for multiple comparisons (adjusted *p* < 0.01). Differential expression analysis between clusters was performed using the MAST algorithm, which accounts for the bimodal expression distribution characteristic of single-cell data. Gene expression patterns within clusters were visualized using dotplots and feature plots to identify cluster-specific markers and pathway components.

### TCGA and GEO datasets description

We retrieved ESCC patient gene expression profiles and clinical information from The Cancer Genome Atlas (TCGA) data portal (https://tcga-data.nci.nih.gov/tcga/). and obtained GSE38129 and GSE67269 microarray datasets from the Gene Expression Omnibus (GEO) database (http://www.ncbi.nlm.nih.gov/geo/). Using the SangerBox platform (http://SangerBox.com/Tool), we analyzed the expression of PTDSS1 in different esophageal tissues and at various stages, and evaluated its correlation with overall survival rate and progression-free survival period.

### Survival and clinicopathological correlation analysis

Clinical outcomes associated with PTDSS1 expression were evaluated using Kaplan–Meier survival curves and log-rank tests. ESCC patient cohorts from TCGA and GEO (GSE53625) were stratified into high- and low-expression groups based on median PTDSS1 levels. Hazard ratios (HRs) and 95% confidence intervals (CIs) were calculated using Cox proportional hazards models. Associations between PTDSS1 expression and tumor stage were analyzed using ANOVA.

### Tissue microarrays and immunohistochemical staining

The tissue microarray procured from Hunan Aifang Biotechnology Co., Ltd. encompassed 80 cases of ESCC tissues, constituted by 0.6 mm diameter perforated blocks. The detailed clinical case information is presented in Table 1. In the immunohistochemistry (IHC) assay, the paraffin-embedded tissue sections were initially dewaxed with xylene and subsequently dehydrated with a gradient ethanol solution. The tissue sections were baked in an oven at 65 °C for 1–2 h, followed by antigen retrieval and incubation with primary antibodies at 4 °C overnight. The sections were then brought back to room temperature and conjugated with biotinylated goat anti-mouse or rabbit anti-IgG (Aifang Biological, Hunan, China) and stained with a DAB staining kit (Solarbio, Beijing, China). After fixation and mounting, the staining intensity was evaluated: cells presenting brownish-yellow staining in the membrane or cytoplasm were regarded as positive. The intensity and proportion of positive cells were scored on a scale from 0 to 3. For each sample, the two scores were multiplied to obtain the final score. Under 200× magnification, the positive stained cells in 5 fields of view were enumerated, and the total number of cells was regarded as the positive cell count. The antibodies are listed in Table S1.

**Table 1:** Distributions of clinical pathological parameters of patients with different expression of PTDSS1.

Clinical pathological parameters	PTDSS1		*P*
	Low expression	High expression	
No. of patients	25	49	-
*Gender*			0.6
Male	23	47	
Female	2	2	
*Age*			0.083
<60	13	15	
≥60	12	34	
*T stage*			**0.003**
1–2	11	6	
3–4	14	43	
*N stage*			**0.0001**
N0–N1	24	27	
N2–N3	1	22	
*M stage*			1.0000
0	24	46	
1	1	3	
*Stage*			**0.021**
I–II	16	17	
III–IV	9	32	
*Grade*			0.314
1–2	2	9	
3	33	40	

### Cell culture, lentivirus construction and plasmid transfection

Kyse30, Kyse150, Eca109 were obtained from Biowing Applied Biotechnology Co., Ltd via STR profiling (Shanghai, China). These cells were cultured in RPMI-1640 medium (Gibco, Grand Island, USA) supplemented with 10% fetal bovine serum (FBS) (Gibco, Grand Island, USA) and 1% penicillin/streptomycin (Invitrogen, Carlsbad, CA, USA) at 37 °C with 5% CO_2_.

The pHBLV-CMV-MCS-EF1-T2A-Puro lentiviral (Hanbio, Shanghai, China) expression vector was constructed and used to infect Kyse30 and Eca109 cells. To enhance the infection efficiency, 5 μg/mL of polybrene (Hanbio, Shanghai, China) was added to the culture medium as an enhancing reagent. After 72 h of infection, 1 μg/mL of puromycin (Sigma-Aldrich, MO, USA) was used for screening for 5 days. The constructed positive plasmid was verified by DNA sequencing.

### Real-time PCR

Total RNA was isolated with Trizol reagent, followed by cDNA synthesis using the SYBR PrimeScript RT-PCR Kit (Takara Biochemicals, Tokyo, Japan). qRT-PCR was performed on a 7500 Real-Time PCR System (Applied Biosystems, Foster City, CA, USA) with β-actin as the endogenous control. Relative mRNA levels were determined via the ΔΔCt method (Ratio = 2 − ΔΔCt). Three independent replicates were performed, with primer sequences detailed in Table S2.

### Western blotting

Cell lysates were prepared with RIPA buffer (Thermo Fisher Scientific, MA, USA) containing PMSF (Beyotime, Shanghai, China). The total proteins of each group (approximately 15 μg) were separated by sodium dodecyl sulfate-polyacrylamide gel electrophoresis (SDS-PAGE) and transferred onto polyvinylidene fluoride (PVDF) membranes (Millipore, Bedford, Massachusetts, USA). The membranes were incubated with primary antibodies (diluted at a ratio of 1:1000) overnight at 4 °C. Subsequently, the membranes were washed three times with TBST for 5 min each time, and then incubated with secondary antibodies at room temperature for 1–2 h. Finally, the membranes were developed using the enhanced chemiluminescence method (ECL, Beyotime, Shanghai, China). The antibodies employed are detailed in Table S2.

### Immunofluorescence

Cells were seeded onto sterile glass coverslips and cultured overnight at 37 °C and 5% CO₂ to promote adhesion. Subsequently, the cells were fixed with pre-cooled methanol for 20 min. After fixation, the cells were blocked with 1% bovine serum albumin (BSA) for 30 min and then incubated with the primary antibody overnight at 4 °C. The next day, the cells were incubated with fluorescently labeled secondary antibodies at room temperature for 30 min. Finally, images were captured using a Zeiss fluorescence microscope (ZEISS, Oberkochen, DE).

### Cell proliferation and TUNEL assay

For the CCK-8 assay, approximately 2000 tumor cells were inoculated into each well of a 96-well plate. In accordance with the aforementioned conditions, 10 µL of CCK-8 reagent (Beyotime, Shanghai, China) was added to each well. Absorbance was determined at a wavelength of 450 nm using a microplate reader. At least three replicate wells were established for each group to ensure the reliability of the data.

For the colony formation experiment, approximately 400 tumor cells were inoculated into each well of a 6-well plate and allowed to grow for 14 days. Upon the conclusion of the incubation period, the cells were fixed with 4% formaldehyde for 20 min and subsequently stained with crystal violet at room temperature for 20 min. Once the staining was completed, the formed colonies were enumerated.

The TUNEL staining kit (Vazyme, Nanjing, China) was employed to identify apoptotic cells in situ. After being treated with 10% goat serum and 0.2% Triton X-100 for 5 min, the labeling reaction containing terminal deoxynucleotidyl transferase was conducted in a humidity chamber at room temperature for 2 h. Subsequently, the nuclei were counterstained with DAPI staining solution at room temperature for 5 min. After the sections were washed with PBS, they were observed using a Zeiss microscope.

### Invasion and wound healing assays

2 × 10^4^ cells were inoculated into the Matrigel-coated membrane chambers (Transwell chambers) and placed within 24-well plates. 100 μl of serum-free medium was added to the upper chamber, and 600 microliters of RPMI-1640 medium containing 10% FBS was incorporated into the lower chamber. The plates were incubated at 37 °C for 48 h. Post-incubation, the floating cells were eliminated, and the cells in the lower chamber were fixed with methanol for 20 min and stained with crystal violet.

In the wound healing experiment, cells were seeded in 6-well plates and cultivated until approximately 90% confluence was achieved. Subsequently, a uniform scratch was formed on the surface of the plate by gently scraping with the tip of a 10 μL pipette. After being washed with PBS, the cells were further maintained in serum-free RPMI-1640 medium. Photographs were captured at 0 and 48 h following scratch formation. Image analysis was conducted using Image-Pro Plus 6.0 software, and the results were presented as the average of three independent experiments.

### RNA library preparation, sequencing, and enrichment analysis

The RNA sequencing of cells was accomplished by Tsingke biotechnology (Tsingke, Beijing, China). All libraries were sequenced on the Illumina HiSeq platform, generating high-quality sequencing data. Each sample was subjected to SNP and INDEL variant detection using the GATK software. Subsequently, hierarchical clustering analysis was employed to systematically analyze the differentially expressed genes (DEGs) to reveal the gene expression patterns among different groups and samples.

### Metabonomics

Metabolomics analysis was conducted by Tsingke biotechnology (Tsingke, Beijing, China). The cells were initially washed three times with sterile pre-cooled PBS solution, followed by extraction with an acetonitrile-methanol solution. Subsequently, they were detected using an ultra-high performance liquid chromatography system (UHPLC, Thermo Vanquish) coupled with a high-resolution mass spectrometer (HRMS, Thermo Q Exactive HF-X). Chromatographic separation was achieved using an HSS T3 column (2.1 × 100 mm, 1.8 μm), with the mobile phase consisting of 0.1% formic acid water (A) - acetonitrile (B) at a flow rate of 0.3 mL/min. Mass spectrometry data were processed for peak extraction, normalization, and metabolite annotation (mzCloud database) via Compound Discoverer 3.3. The criteria for screening differential metabolites were VIP > 1.0 (OPLS-DA model) and *p* < 0.05 (t-test). Metabolic pathway analysis was accomplished through the KEGG database and MetaboAnalyst 5.0.

### Glutathione (GSH) detection assay

In accordance with the instructions provided by the Glutathione (GSH) and Oxidized Glutathione (GSSG) Assay Kit (Beyotime Biotechnology, Shanghai, China), the level of glutathione in cell or tumor lysates was measured.

### Intracellular reactive oxygen species (ROS) detection assay

ROS was determined by employing the reactive oxygen species detection kit (Beyotime Biotechnology, Shanghai, China) in accordance with the operational guidelines provided by the manufacturer. The specific procedures were as follows: Initially, the cell samples were collected and incubated with DCFH-DA at 37 °C for 30 min; subsequently, the cells were washed with serum-free medium to eliminate the unbound dye. Eventually, the DCF fluorescence intensity in the 1 × 10^7^/mL cell suspension was detected using flow cytometry with an excitation wavelength of 488 nm and an emission wavelength of 525 nm, or observed through fluorescence microscopy.

### Extracellular flux analysis

All experiments were conducted using a Seahorse XFp extracellular flux analyzer (Agilent). Cells were seeded (10,000 to 25,000 cells per well) in XFp mini-plates (Agilent) and allowed to adhere overnight. For the analysis of mitochondrial oxygen consumption rate (OCR, pmol/min), cells were maintained in unbuffered serum-free DMEM (Basal DMEM, Agilent) supplemented with pyruvate (1 mM), glutamine (2 mM), glucose (10 mM), at pH 7.4 and 37 °C, and ambient CO_2_ for 1 h prior to the assay. During the assay, cells were successively subjected to stress with oligomycin (1 µM), FCCP (1.0 µM, except 0.5 µM for MiaPaCa-2), and a mixture of rotenone/antimycin A (0.5 µM each). All results were normalized based on the cell number evaluated by Hoechst (2 µg/mL) incorporation after cold methanol/acetone fixation. The results presented are representative ones among three independent experiments.

### Mitochondrial membrane potential assay

Cells were inoculated in 6-well plates at a density of 2 × 10^5^ cells per well. The JC-1 fluorescent probe was assembled in accordance with the instructions of the kit (Beyotime Biotechnology, Shanghai, China), and images were acquired via fluorescence microscopy. When the mitochondrial membrane potential was high, JC-1 aggregated in the mitochondrial matrix to form polymers, exhibiting red fluorescence; whereas when the mitochondrial membrane potential was low, JC-1 was unable to aggregate in the mitochondrial matrix and existed in monomeric form, thereby green fluorescence was observed. The change in mitochondrial membrane potential could be detected based on the variation in fluorescence color.

### Transmission electron microscopy

Cells were inoculated in 6-well plates at a density of 2 × 10^6^ cells per well and cultivated in a 37 °C, 5% CO_2_ incubator for 24 h. Subsequently, the supernatant was removed, and 1 mL of fixative was added to each well, followed by fixation at room temperature for 2–4 h. Once the fixation was completed, the cells were scraped off and collected in 15 mL centrifuge tubes, and centrifuged at 800 rpm for 5 min. The obtained cell pellets were subjected to secondary fixation with 2% osmium tetroxide and then underwent gradient dehydration through successive solutions of varying concentrations of ethanol and propylene oxide. Subsequently, the cell pellets were embedded in epoxy resin and polymerized at 60 °C for 20 h. Ultrathin sections were placed on 200-mesh copper grids and double-stained with lead citrate and uranyl acetate. Ultimately, the ultrathin sections were observed using a transmission electron microscope.

### Cell Ferrous Iron (Fe^2+^) assay

Cells were inoculated into 24-well plates at an appropriate density and subjected to overnight culturing in a 37 °C, 5% CO_2_ incubator. Subsequently, the supernatant was discarded, and the cells were rinsed 2 to 3 times with PBS. In accordance with the manual of the Cell Ferrous Iron (Fe^2+^) Assay Kit (MedChemexpress, New Jersey, USA), the cells were washed 2 to 3 times anew with staining buffer. Adequate staining working solution was added to ensure full coverage of the monolayer cells. The cells were incubated in a 37 °C incubator for 20 to 60 min. Ultimately, the cells were observed using a fluorescence microscope.

### Detection of malondialdehyde (MDA) level

After collecting the cells, use the cell lysis buffer to fully lyse the cells. Then, centrifuge the lysate at 10,000–12,000 × *g* for 10 min and keep the supernatant for later use. According to the instructions of the Beyotime Lipid Peroxidation Assay Kit (Beyotime Biotechnology, Shanghai, China), prepare the detection working solution and mix it thoroughly with the supernatant. Place the mixture in a 100 °C water bath or boiling water bath and heat for 15 min. After cooling to room temperature, centrifuge at 1000 × *g* for 10 min at room temperature. Take 200 μL of the supernatant and add it to a 96-well plate. Use a microplate reader to measure the absorbance at 532 nm.

### Lipid peroxidation assay

Cells were digested using trypsin and collected in 1.5 mL centrifuge tubes. Subsequently, they were centrifuged at 800 rpm for 5 min at room temperature. The supernatant was discarded, and the cells were washed twice with 1 mL of PBS, followed by centrifugation under the same conditions each time. Thereafter, the cells were resuspended in 200 μL of PBS and transferred to round-bottom 96-well plates. Next, the cells were centrifuged once more at 800 rpm for 5 min, the supernatant was removed, and 200 μL of staining working solution containing 5 μM BODIPY (Thermo, Massachusetts, USA) was added. The cells were then incubated in a 37 °C, 5% CO_2_ incubator for 30 min. After the incubation period, the cells were washed twice with 200 μL of PBS and ultimately resuspended in 200 μL of PBS. Lipid peroxidation levels were detected via flow cytometry (excitation wavelength: 665 nm, emission wavelength: 676 nm).

### MS and co-IP assays

Cells were first washed with phosphate-buffered saline (PBS) and subsequently lysed using IP lysis buffer (Thermo Fisher Scientific, USA). For the immunoprecipitation (IP) experiments, Protein G magnetic beads (pre-washed with IP buffer) were incubated with antibodies (IgG from CST, USA; Flag antibody from Sigma-Aldrich, USA). A portion of the cell lysate was reserved as input, while the remaining lysate was incubated with the antibody-conjugated beads overnight at 4 °C. The next day, the immune complexes were separated from the beads using a magnetic rack and then boiled for 15 min in SDS-PAGE loading buffer. Purified protein complexes were resolved by SDS-PAGE and stained using the Fast Silver Stain Kit (Beyotime, China). Gel slices containing the proteins of interest were excised and sent to Oebiotech (Shanghai, China) for subsequent MS analysis. Proteins of interest in the Co-IP samples were further validated by Western blotting.

### Multispectral fluorescent immunohistochemistry

We conducted multispectral immunofluorescence staining in accordance with the instructions provided by the multiplex immunofluorescence kit (Aifang Biology, Hubei, China). In brief, the slides were heated, dewaxed with xylene, and rehydrated using a gradient of alcohol. After antigen retrieval and blocking, the primary antibodies were added and incubated overnight at 4 °C. Opal polymer horseradish peroxidase (HRP) was utilized as the secondary antibody. Once the slides were washed, tyramide signal amplification (TSA) dyes were applied. Subsequently, the primary and secondary antibodies were eliminated using a microwave oven, and the slides were washed and blocked again. Next, the second primary antibody and 4’,6-diamidino-2-phenylindole (DAPI) were introduced. Eventually, the slides were covered with ProLong Gold antifade reagent and inspected using a TissueGnostics automated multispectral microscope. Five fields of view at 200× magnification of the monochromatic slides were imaged, and a spectral library for unmixing was generated by employing StrataQuest image analysis software (v.6.0.1.181). Subsequently, the index cases stained with the multiplex staining method were imaged. The channels were unmixed by means of the spectral library, and the tissues and cells were segmented and scored.

### Mouse models

Four-week-old BALB/c nude mice, with a weight ranging approximately from 16.5 to 17.5 g, were furnished by Nanjing Charles River Laboratory Animal Technology Co., Ltd. All the nude mice were reared in SPF-level animal facilities. We procured lentiviral cells carrying distinct constructs and mixed 5 × 10^5^ cells with 100 μL of PBS and Matrigel, followed by subcutaneous injection into the lateral abdominal regions of BALB/c nude mice. Once the average tumor volume attained 50 mm³, subsequent treatments were initiated: RSL3 (5 mg/kg/d) daily by intraperitoneal injection and PTDSS1 inhibitor DS68591889 (30 mg/kg/d) were administered daily by gavage. After the mouse model was established, the body weight and tumor size of each mouse were monitored every four days. The tumor volume (V) was computed based on the formula V = 0.5 × length × width². All animal experiments were stringently carried out in compliance with the “Guide for the Care and Use of Laboratory Animals” compiled by the National Academy of Sciences and the National Institutes of Health (NIH Publication 86-23, revised in 1985) to guarantee that the animals received humane care.

### Statistical analysis

Statistical analyses were performed in Python (v4.3.1). Adjusted *p* < 0.05 were considered statistically significant unless otherwise specified. Data were analyzed by employing SPSS 22.0 and GraphPad Prism 8.0 software in accordance with the manufacturer’s instructions. The measurement data were presented as median (interquartile range), and comparisons were made using the chi-square test. The quantitative data were expressed as mean ± standard deviation and compared through analysis of variance and the least significant difference test. Spearman’s rank correlation test and linear regression analysis were adopted to assess the correlation between the expression levels detected by qPCR. Univariate and multivariate Cox regression analyses were utilized to identify the common genes associated with overall survival. The cumulative survival rates were determined via the Kaplan–Meier method. Statistical significance was set at *p* < 0.05.

## Results

### High PTDSS1 expression was associated with the malignant progression of ESCC

**Fig. 1:**
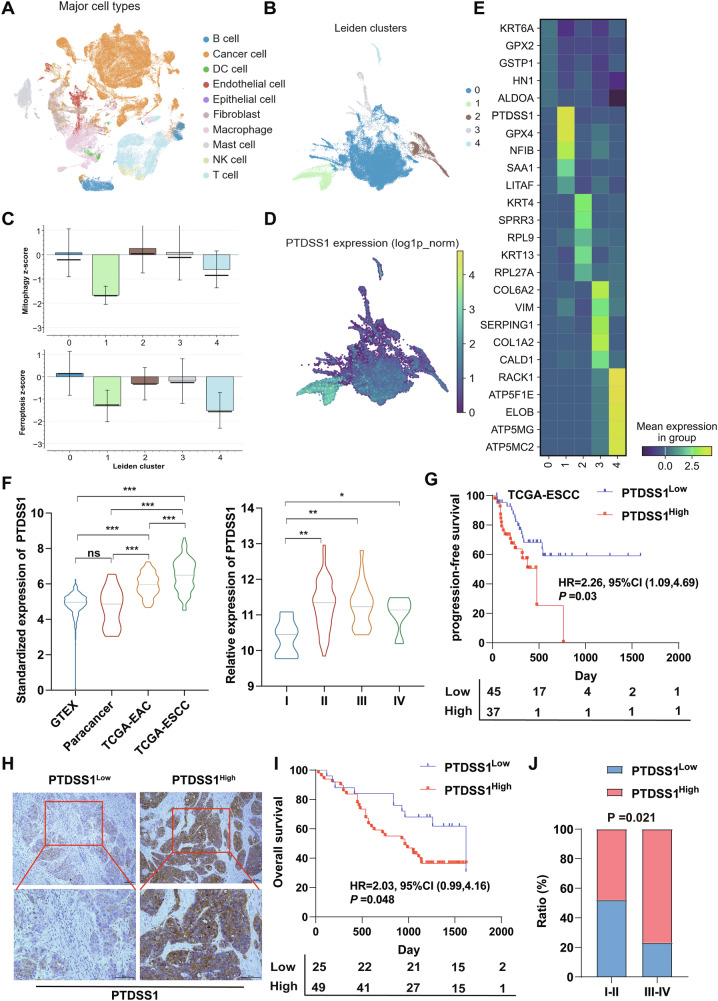
High PTDSS1 expression was associated with the malignant progression of ESCC. **A** UMAP visualization of 59,280 malignant cells identified from three esophageal squamous cell carcinoma single-cell datasets (GSE160269, GSE188990 and GSE197677) after quality control and batch effect correction. **B** Unsupervised clustering revealing five distinct cancer cell subpopulations. **C** Bar plot showing pathway enrichment analysis of ferroptosis and mitophagy pathway activity across the five clusters. **D** Dot plot visualization of PTDSS1 expression levels across the identified cell clusters. **E** Heatmap showing the top 5 marker genes for each subpopulation cluster, highlighting the distinctive molecular signatures of each cancer cell subset. **F** Violin plots showing PTDSS1 expression: left panel displays expression across different tissue types; right panel shows expression across different TCGA-ESCC stages. **G** Kaplan–Meier survival analysis based on PTDSS1 expression levels. **H** The protein expression of PTDSS1 in the tissue microarray. Scale bar: 100 μm. **I**, **J** The correlation of PTDSS1 expression in tissue microarrays with the prognosis and staging of ESCC. *P < 0.05, **P < 0.01, ***P < 0.001.

To comprehensively analyze ESCC at the single-cell level, we integrated datasets from three Gene Expression Omnibus (GEO) databases (GSE160269, GSE188990, and GSE197677). These datasets underwent rigorous quality control and batch effect correction (Fig. S1A). We performed graph-based clustering, with Leiden clustering used for further refinement. To accurately identify cancer cells, we applied both CellTypist-assisted manual annotation and the widely used CancerFinder-scf algorithm, identifying 59,280 malignant cells (Fig. 1A). Cell type identity was confirmed through marker gene expression analysis across all identified cell populations (Fig. S1B). Unsupervised clustering analysis of the identified cancer cells revealed five transcriptionally distinct subpopulations with unique molecular signatures (Fig. 1B). To characterize their functional differences, we quantified ferroptosis and mitophagy pathway activity using the Z-score method, revealing significant enrichment in Leiden cluster 1 (Fig. 1C). Notably, PTDSS1 expression was significantly elevated in this cluster, a finding we further validated through dotplot visualization (Fig. 1D, E), which also showed correlations with key ferroptosis-related genes including GPX4 and ACSL4 (Fig. S1C).

Further analysis of PTDSS1 expression across different tissue types using data from TCGA-EAC, TCGA-ESCC, and GTEx, as well as using data from four GEO databases, revealed significant associations with disease progression (Fig. 1F, Fig. S1D). Analysis of the TCGA-ESCC cohort demonstrated that PTDSS1 expression was significantly upregulated in mid-to-late-stage ESCC patients compared to early-stage counterparts (Fig. 1F). Kaplan–Meier analysis demonstrated that elevated PTDSS1 expression correlates with poorer prognosis in ESCC, while this association was less pronounced in ESA (Fig. 1G, Fig. S1E). To validate our bioinformatic findings at the protein level, we performed immunohistochemical (IHC) staining on 74 ESCC microarray tissues, categorizing samples into high and low expression groups based on PTDSS1 staining intensity (Fig. 1H). Our analysis confirmed a significant association between high PTDSS1 expression and unfavorable prognosis, as well as strong correlations with tumor stage, size and lymph node metastasis, though not with distant metastasis (Fig. 1I, J, Table 1). These findings suggest that PTDSS1 contributes to ESCC malignant progression.

### Targeting PTDSS1 exerts tumor suppressor effects in esophageal squamous cell carcinoma

**Fig. 2:**
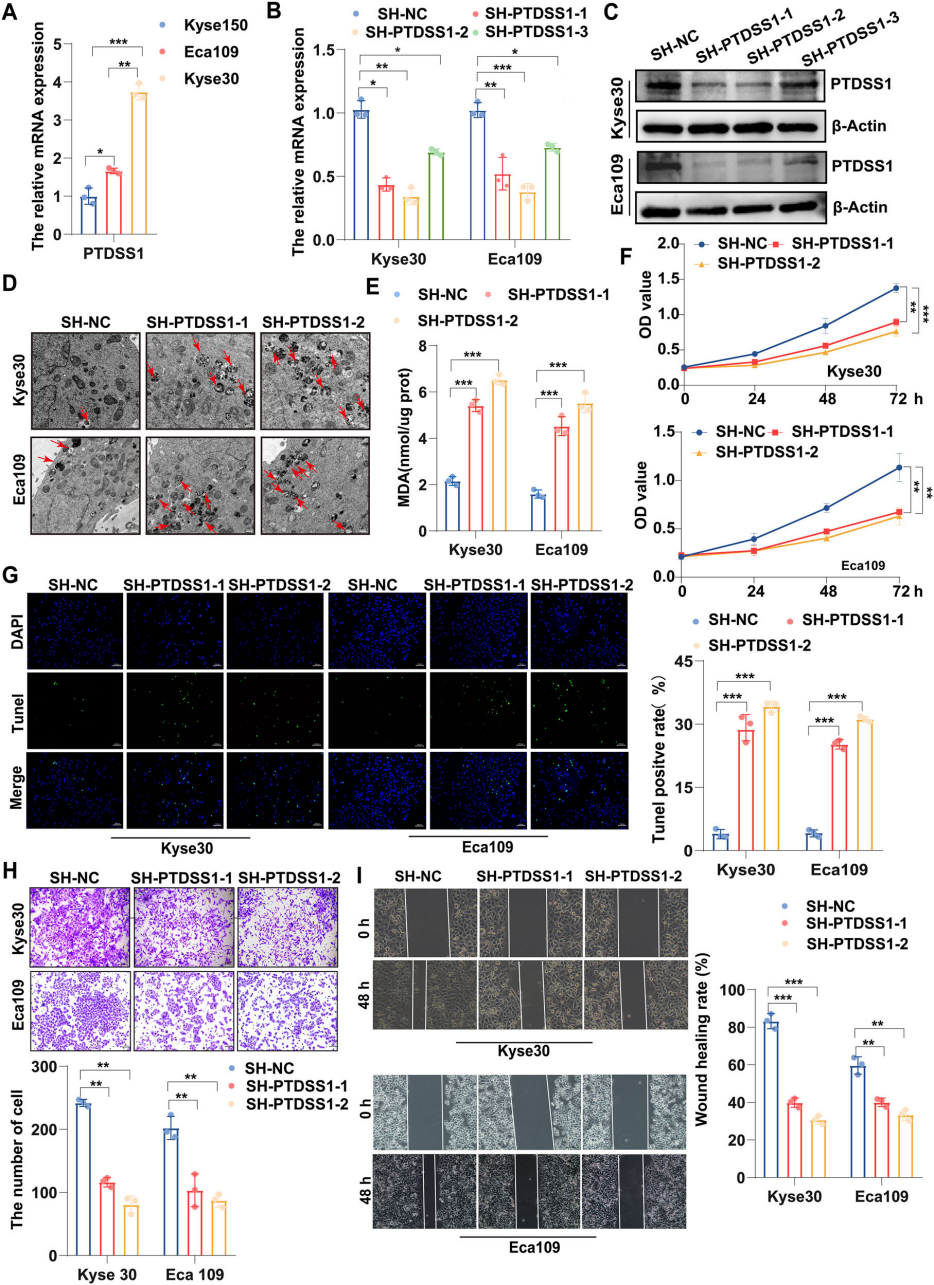
Targeting PTDSS1 exerts tumor suppressor effects in ESCC. **A** PTDSS1 expression in three ESCC cell lines. **B**, **C** PTDSS1 knockdown efficiency in Kyse30 and Eca109 cell lines verified by RT-PCR and Western blot. **D** Mitochondrial ultrastructural changes observed by TEM. Red arrows indicate mitophagy. Scale bar: 500 nm. **E** Intracellular MDA concentration. **F** Cell proliferation measured by CCK8 assay. **G** Apoptosis detected by TUNEL assay. Scale bar: 100 μm. **H** Representative images of transwell invasion assays. Scale bar: 100 μm. **I** Representative images of wound healing assays. Scale bar: 100 μm. *P < 0.05, **P < 0.01, ***P < 0.001.

To validate the role of PTDSS1 in ESCC, we selected two cell lines (Kyse30 and Eca109) with high expression of PTDSS1 from three ESCC cell lines and conducted transfection using lentiviral vectors specifically interfering with the PTDSS1 gene (Kyse30^SH-PTDSS1^ and Eca109^SH-PTDSS1^) (Fig. 2A). Kyse30^SH-PTDSS1^ and Eca109^SH-PTDSS1^ cells exhibited marked reduction in PTDSS1 expression at both mRNA and protein levels compared to controls (Fig. 2B, C). Transmission electron microscopy (TEM) analysis revealed that mitophagy activity and malondialdehyde (MDA) levels were significantly increased in the PTDSS1-knockdown group compared to controls, whereas PTDSS1 overexpression markedly suppressed these phenomena (Fig. 2D, E, Fig. S2A, B). Cell Counting Kit 8 (CCK8) assays revealed significantly attenuated proliferative capacity in Kyse30^SH-PTDSS1^ and Eca109^SH-PTDSS1^ cells versus controls, while PTDSS1 overexpression enhanced tumor cell clonogenicity (Fig. 2F, Fig. S2C). TUNEL assays demonstrated pro-apoptotic effects of PTDSS1 knockdown and anti-apoptotic activity upon its overexpression (Fig. 2G, Fig. S2D). The results of trans-well invasion and wound healing assay showed that PTDSS1 knockdown significantly reduced in the number of cells migrating through the matrigel layer after 48 h and decreased the wound closure rates (WCRs), while overexpression of PTDSS1 accelerated cell invasion and migration abilities (Fig. 2H-I, Fig. S2E, F). In summary, PTDSS1 inhibits mitophagy and ferroptosis, promotes cell proliferation, suppresses apoptosis, and enhances cell invasion and migration capabilities. These findings suggest that targeting PTDSS1 can achieve significant anti-cancer effects.

### Targeting PTDSS1 leads to GSH depletion and mitochondrial dysfunction

**Fig. 3:**
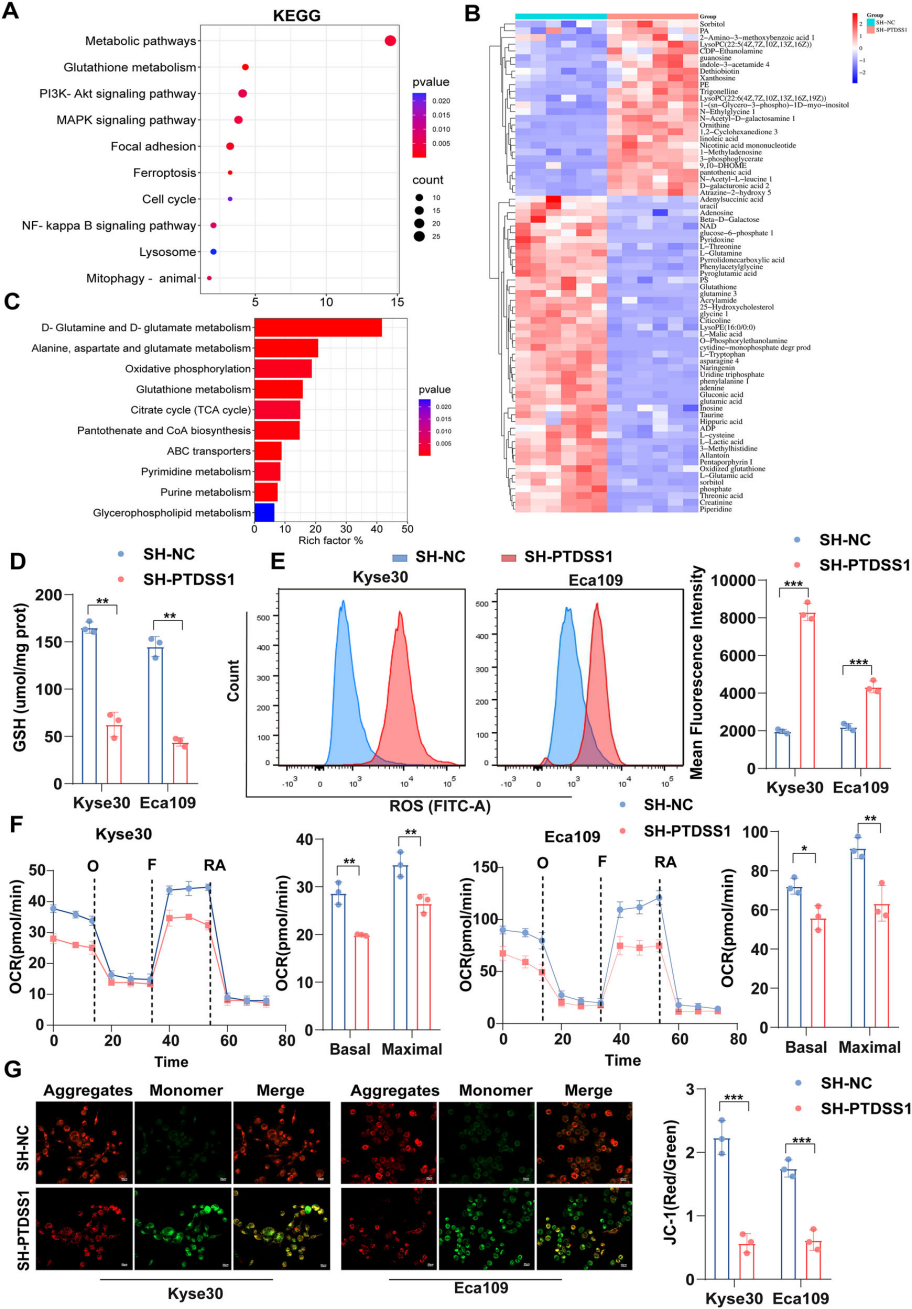
PTDSS1 inhibition mainly leads to GSH depletion and mitochondrial dysfunction. **A** KEGG enrichment analysis of differentially expressed genes in Kyse30 cell transcriptome. **B** Cluster analysis of differential metabolites in Kyse3^SH-PTDSS1^versus control cells. **C** KEGG pathway enrichment of differential metabolites in Kyse30^SH-PTDSS1^ versus control cells. **D** Intracellular GSH content in SH-PTDSS1 versus control cells. **E** Flow cytometry quantification of intracellular ROS in SH-PTDSS1 versus control cells. **F** Oxygen consumption rate (OCR) in SH-PTDSS1 versus control cells. **G** Mitochondrial membrane potential (MMP) in SH-PTDSS1 versus control cells. Scale bar: 20 μm. *P < 0.05, **P < 0.01, ***P < 0.001.

PS catalyzed by PTDSS1 is crucial for maintaining mitochondrial functional stability and cellular structural homeostasis [16, 28]. To explore the regulatory mechanism of PTDSS1, we collected Kyse30^SH-PTDSS1^ and its control cell lines for transcriptome sequencing. KEGG pathway enrichment analysis showed that the majority of differentially expressed genes (DEGs) were enriched in metabolism-related pathways, particularly those involved in glutathione metabolism, ferroptosis and mitophagy (Fig. 3A). Parallel analysis of high-expression and low-expression groups of PTDSS1 from the TCGA database confirmed that these DEGs were predominantly enriched in metabolism-related pathways, with a strong emphasis on glutathione metabolism and mitochondrial metabolism (Fig. S3A). These findings imply that PTDSS1 plays a significant role in tumor metabolism regulation.

Further metabolomics analysis of Kyse30^SH-PTDSS1^ cell line compared with the control group revealed significant alterations in three metabolic pathways, glutamine-glutamate metabolism, glutathione metabolism, and oxidative phosphorylation (Fig. 3B, C). Notably, PTDSS1 knockdown led to a marked reduction in key metabolites for glutathione synthesis, including L-glutamine, L-glutamate, L-cysteine, and glycine, suggesting a potential role of PTDSS1 in the regulation of GSH synthesis (Fig. S3B). The GSH system serves as a primary antioxidant mechanism within cells, and in cancer cells, and elevated GSH levels in cancer cells are crucial for eliminating excessive ROS and detoxifying exogenous substances [29, 30]. GSH depletion leads to ROS accumulation, inducing oxidative stress, mitochondrial dysfunction, and ferroptosis [30, 31]. making the glutathione system an attractive target for cancer treatment [32]. We observed that PTDSS1-knockdown cells exhibited significantly lower GSH levels and elevated ROS levels (Fig. 3D, E), demonstrating that PTDSS1 is essential for maintaining the cellular redox balance. Importantly, overexpression of PTDSS1 reversed these effects (Fig. S3C, D).

To further investigate how PTDSS1 affects mitochondrial function, we assessed mitochondrial oxidative phosphorylation (OXPHOS) using the Seahorse XF96 analyzer. PTDSS1 knockdown caused a significant decrease in oxygen consumption rate (OCR), basal respiration, and maximal respiratory capacity, accompanied by a reduction in mitochondrial membrane potential (MMP) (Fig. 3F, G). Conversely, PTDSS1 overexpression significantly augmented OCR, basal respiration, and maximal respiratory capacity, as well as enhanced MMP (Fig. S3E, F). These results suggest that targeting PTDSS1 may primarily disrupts GSH synthesis, leading to excessive ROS accumulation, which ultimately results in oxidative stress, impaired mitochondrial oxidative phosphorylation, and mitochondrial dysfunction.

### The GSH depletion caused by knockdown of PTDSS1 can induce mitophagy

**Fig. 4:**
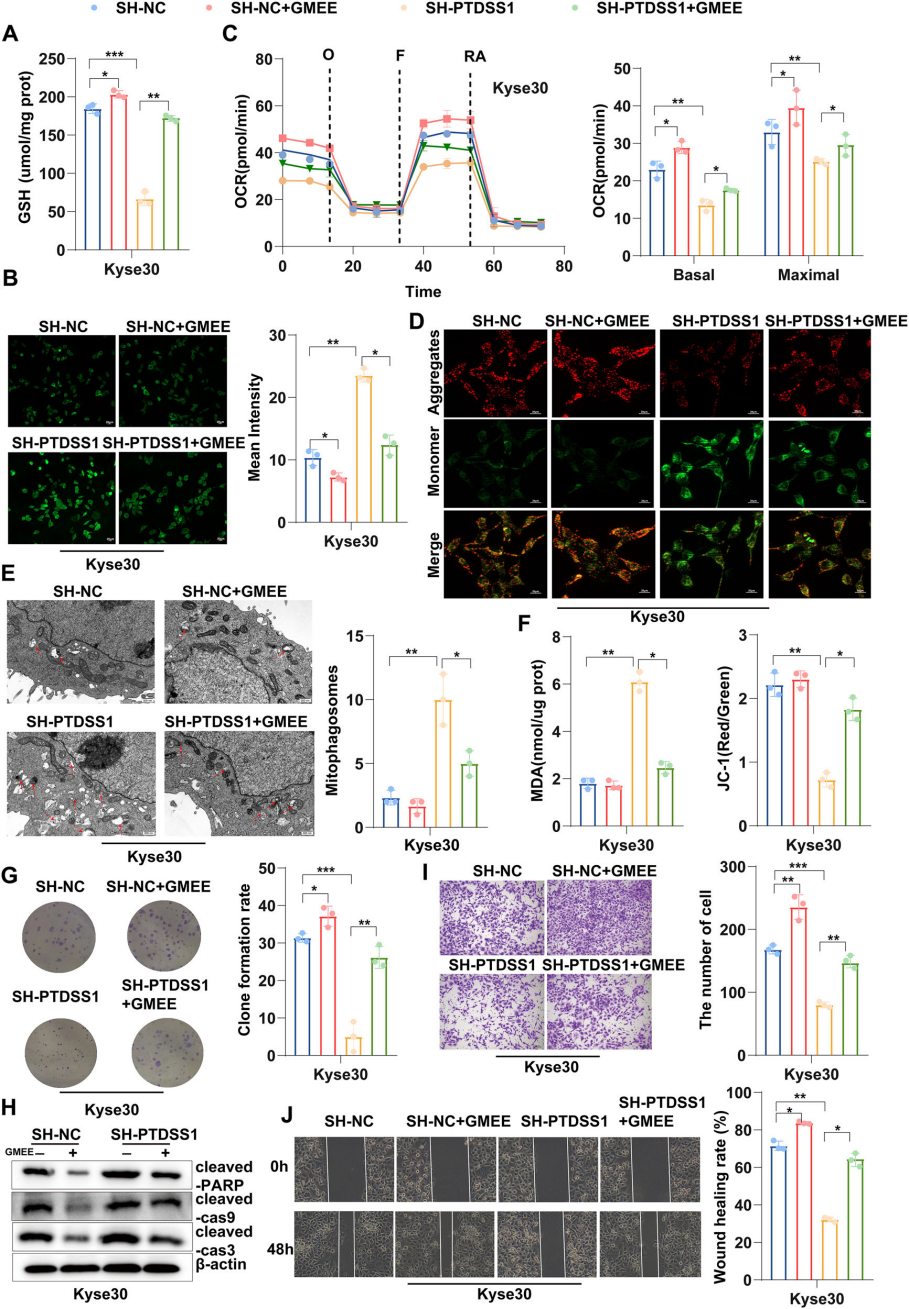
The GSH depletion caused by knockdown of PTDSS1 can induce mitophagy. **A** Intracellular GSH content in SH-PTDSS1 and control Kyse30 cells with or without 10 mM glutathione monoethyl ester (GMEE) supplementation. **B** Intracellular ROS levels after 10 mM GMEE supplementation. Scale bar: 20 μm. **C** OCR in SH-PTDSS1 and control cells after 10 mM GMEE supplementation. **D** MMP after 10 mM GMEE treatment. Scale bar: 20 μm. **E** Mitochondrial ultrastructural changes observed by TEM after 10 mM GMEE supplementation; red arrows indicate mitophagy. Scale bar: 500 nm. **F** Intracellular MDA concentration in Kyse30 cells after 10 mM GMEE treatment. **G** Colony size and number in Kyse30 cells after 10 mM GMEE treatment. **H** Apoptotic protein expression assessed by Western blot after 10 mM GMEE treatment in Kyse30 cells. **I** Representative images of transwell invasion assays after 10 mM GMEE treatment in Kyse30 cells. **J** Representative images of wound healing assays after 10 mM GMEE treatment in Kyse30 cells. Scale bar: 100 μm. *P < 0.05, **P < 0.01, ***P < 0.001.

GSH depletion can induce oxidative stress, leading to reduced mitochondrial oxidative phosphorylation capacity and resultant mitochondrial dysfunction [33]. To confirm that GSH depletion was the main driver of oxidative stress and mitochondrial impairment, we supplemented glutathione monoethyl ester (GMEE) in PTDSS1-knockdown cells and control cells to restore intracellular GSH levels (Fig. 4A, Fig. S4A). As expected, GMEE supplementation significantly decreased intracellular ROS levels (Fig. 4B, Fig. S4B), confirming that reduced GSH inhibits ROS accumulation and reduces oxidative stress. Moreover, mitochondrial function was largely restored, with GMEE treatment reversing the decline in OCR, basal respiration, and maximal respiratory capacity caused by PTDSS1 knockdown, as well as MMP (Fig. 4C, D, Fig. S4C, D).

Excessive ROS accumulation has been shown to trigger mitochondrial dysfunction [34]. and in our study, we observed that PTDSS1 knockdown caused extensive mitophagy, a process through which damaged mitochondria and mtROS are removed [35]. TEM analysis revealed that the mitochondrial cristae structure was disordered in PTDSS1-knockdown cells, with numerous blank areas indicating mitophagy. Importantly, GMEE supplementation inhibited mitophagy process (Fig. 4E, Fig. S4E), further emphasizing that GSH depletion is a key regulator of mitophagy in PTDSS1-deficient cells. Additionally, PTDSS1 knockdown significantly increased the levels of MDA, a lipid peroxidation product and ferroptosis marker, which was effectively reversed by GMEE supplementation (Fig. 4F, Fig. S4F). Functionally, GMEE supplementation reversed the anti-tumor effects of PTDSS1 depletion, including inhibition of cell proliferation, reduced invasion and migration, and increased apoptosis (Fig. 4G–J, Fig. S4G–J). These findings demonstrate that PTDSS1 depletion induces GSH depletion, leading to ROS accumulation, mitochondrial dysfunction, excessive mitophagy, and ultimately triggering ferroptosis and apoptosis, thereby exerting an anti-tumor effect.

### The depletion of GSH resulting from the knockdown of PTDSS1 can induce mitophagy via the PINK1/Parkin/MFN2 pathway to promote ferroptosis and apoptosis

**Fig. 5:**
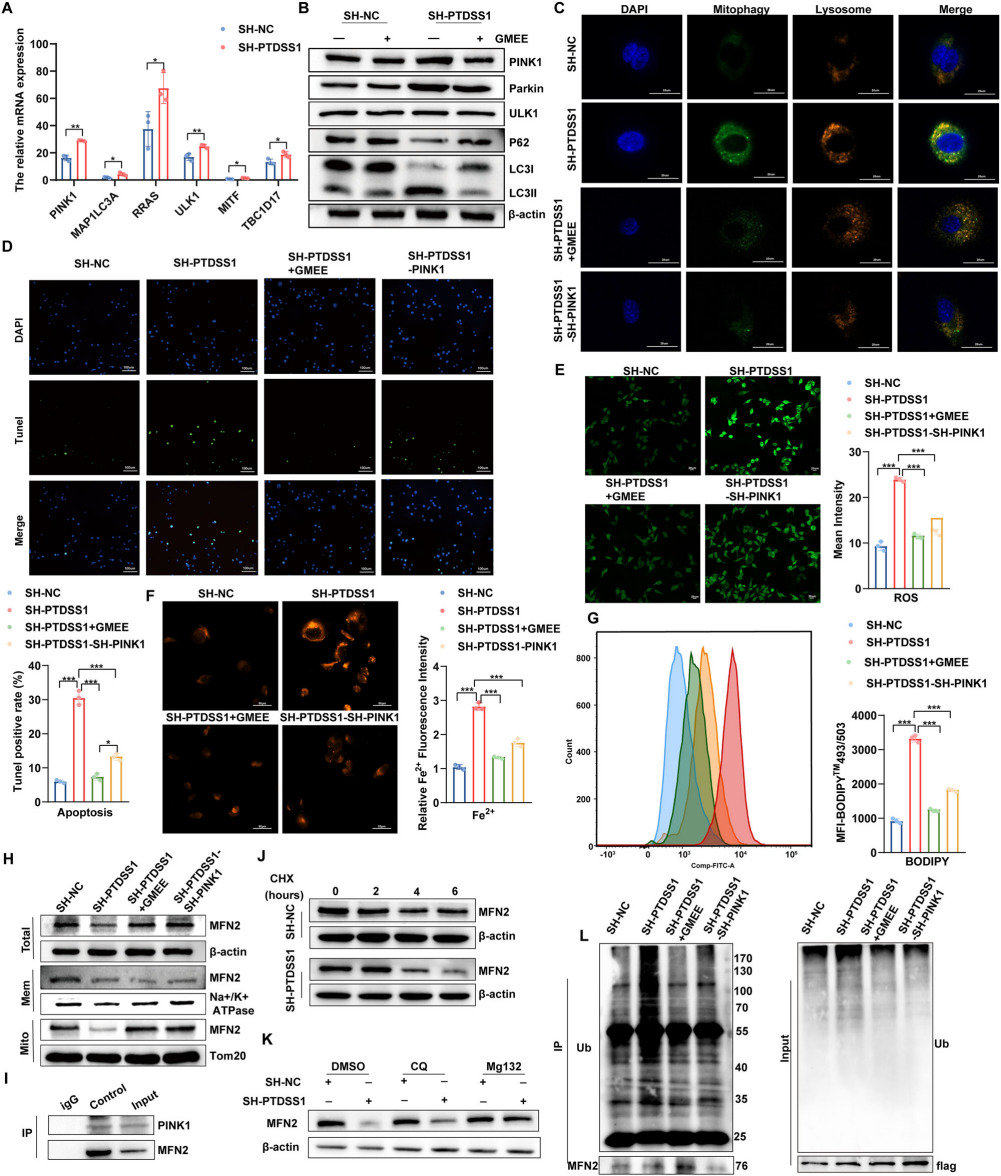
PTDSS1 knockdown-induced GSH depletion activates the PINK1/Parkin/MFN2 mitophagy pathway, promoting ferroptosis and apoptosis. **A** Differential expression of mitophagy pathway genes in the transcriptome. **B** Expression of mitophagy-related proteins (PINK1, Parkin, ULK1, P62, LC3I/II) in Kyse30^SH-PTDSS1^ versus Kyse30^SH-NC^ cells after 10 mM GMEE treatment, assessed by Western blot. **C** Co-localization of lysosomes and mitochondria in Kyse30^SH-PTDSS1^ cells after 10 mM GMEE treatment or PINK1 knockdown. Scale bar: 20 μm. **D** Apoptosis in Kyse30^SH-PTDSS1^ cells after 10 mM GMEE treatment or PINK1 knockdown. Scale bar: 100 μm. **E** Intracellular ROS levels in Kyse30^SH-PTDSS1^ cells after 10 mM GMEE treatment or PINK1 knockdown. Scale bar: 20 μm. **F** Intracellular Fe^2+^ levels in Kyse30^SH-PTDSS1^ cells treated with 10 mM GMEE or PINK1 knockdown. Scale bar: 50 μm. **G** Lipid peroxidation in Kyse30^SH-PTDSS1^ cells after 10 mM GMEE treatment or PINK1 knockdown, assessed by flow cytometry. **H** MFN2 protein expression in isolated mitochondrial and plasma membranes by Western blot. **I** PINK1-MFN2 interaction in Kyse30 cells demonstrated by co-immunoprecipitation. **J** Cycloheximide (CHX) chase assay for MFN2 in SH-NC or SH-PTDSS1 infected Kyse30 cells treated with CHX (500 μg/ml) for indicated times. **K** MFN2 expression in SH-NC or SH-PTDSS1 Kyse30 cells after treatment with 10 μM chloroquine (CQ) or 10 μM MG132 for 5 h. **L** MFN2 ubiquitination levels in different treatment groups assessed by immunoprecipitation; MG132 (10 μM) was added 5 h before cell harvest. *P < 0.05, **P < 0.01, ***P < 0.001.

Transcriptome analysis revealed that mitophagy-related pathways were significantly upregulated in PTDSS1 knockdown cells (Fig. 5A). Specifically, PINK1 and Parkin levels were significantly increased, with ULK1 slightly elevated, higher LC3B-II/LC3B-I ratio, and reduced P62 expression. Importantly, GMEE treatment significantly suppressed the PINK1/Parkin-mediated mitophagy pathway (Fig. 5B), indicating that PTDSS1 knockdown activates this pathway via GSH depletion in tumor cells, which plays a key role in ferroptosis and apoptosis [24, 27]. For further confirmation, mitophagy was quantified using lysosomal and mitophagy-specific dyes, and cell viability was assessed. PTDSS1 knockdown increased the colocalization of lysosomal and mitophagy markers, inhibited cell proliferation, and activated apoptosis-related proteins, leading to an increase in apoptotic cells. These effects were reversed by either GMEE supplementation or PINK1 knockout (Fig. 5C, D, Fig. S5A, B). Ferroptosis, induced by ROS and Fe^2+^ accumulation, is dependent on lipid peroxidation caused by Fe^2+^ buildup. We found that PTDSS1 knockdown significantly increased intracellular ROS levels, Fe^2+^ accumulation, and lipid peroxidation, but both GMEE supplementation and PINK1 intervention alleviated ferroptosis (Fig. 5E–G, Fig. S5C). These results suggest that inhibiting oxidative stress and mitophagy can mitigate ferroptosis and apoptosis caused by PTDSS1 knockdown.

PTDSS1 and PTDSS2 synthesize PS using phosphatidylcholine (PC) and phosphatidylethanolamine (PE) as substrates [36–38]. Once synthesized in the mitochondrial-associated membranes (MAM) of the endoplasmic reticulum (ER), PS is transported to the mitochondria to maintain mitochondrial function or to the plasma membrane (PM) [39]. Notably, PS from the ER can specifically bind to MFN2 for transport to the mitochondrial outer membrane, where MFN2 becomes a target of Parkin-mediated ubiquitination and mitophagy [16, 40, 41]. In PTDSS1 knockdown cells, we observed a significant decrease in PTDSS1 expression, while PTDSS2 levels remained unchanged. Metabolomics analysis further confirmed a significant reduction in cellular PS levels (Fig. S5D–F). Importantly, PTDSS1 did not regulate MFN2 expression at the mRNA level (Fig. S5G). After isolating mitochondria and other membrane fractions to examine MFN2 expression, we found that GMEE supplementation or PINK1 knockdown restored MFN2 expression on the mitochondrial membrane, while no changes were detected in other membranes (Fig. 5H). These results suggest that PS deficiency promotes the transfer of MFN2 from the plasma membrane to the mitochondrial membrane, where it undergoes degradation. STRING database analysis showed an interaction between MFN2 and PINK1, which was confirmed by co-immunoprecipitation (Fig. S5H, Fig. 5I). Cycloheximide (CHX) chase assay revealed that PTDSS1 knockout significantly shortened the half-life of MFN2 (Fig. 5J, Fig. S5I). Additionally, MG132, a proteasome inhibitor, but not chloroquine (CQ), reversed MFN2 downregulation caused by PTDSS1 knockout (Fig. 5K). Immunoprecipitation (IP) experiments confirmed that PTDSS1 knockout increased MFN2 ubiquitination, an effect reversed by GMEE supplementation or PINK1 knockout, suggesting that PTDSS1 knockdown promotes MFN2 binding to PINK1 and its degradation via the ubiquitin-proteasome pathway (Fig. 5L).

In summary, PTDSS1 deficiency induces GSH depletion and oxidative stress, activating the PINK1/Parkin/MFN2 mitophagy pathway and promoting ferroptosis and apoptosis in tumor cells.

### The knockdown of PTDSS1 regulates the generation of GSH by facilitating the combination of TRIM21 and SLC3A2

**Fig. 6:**
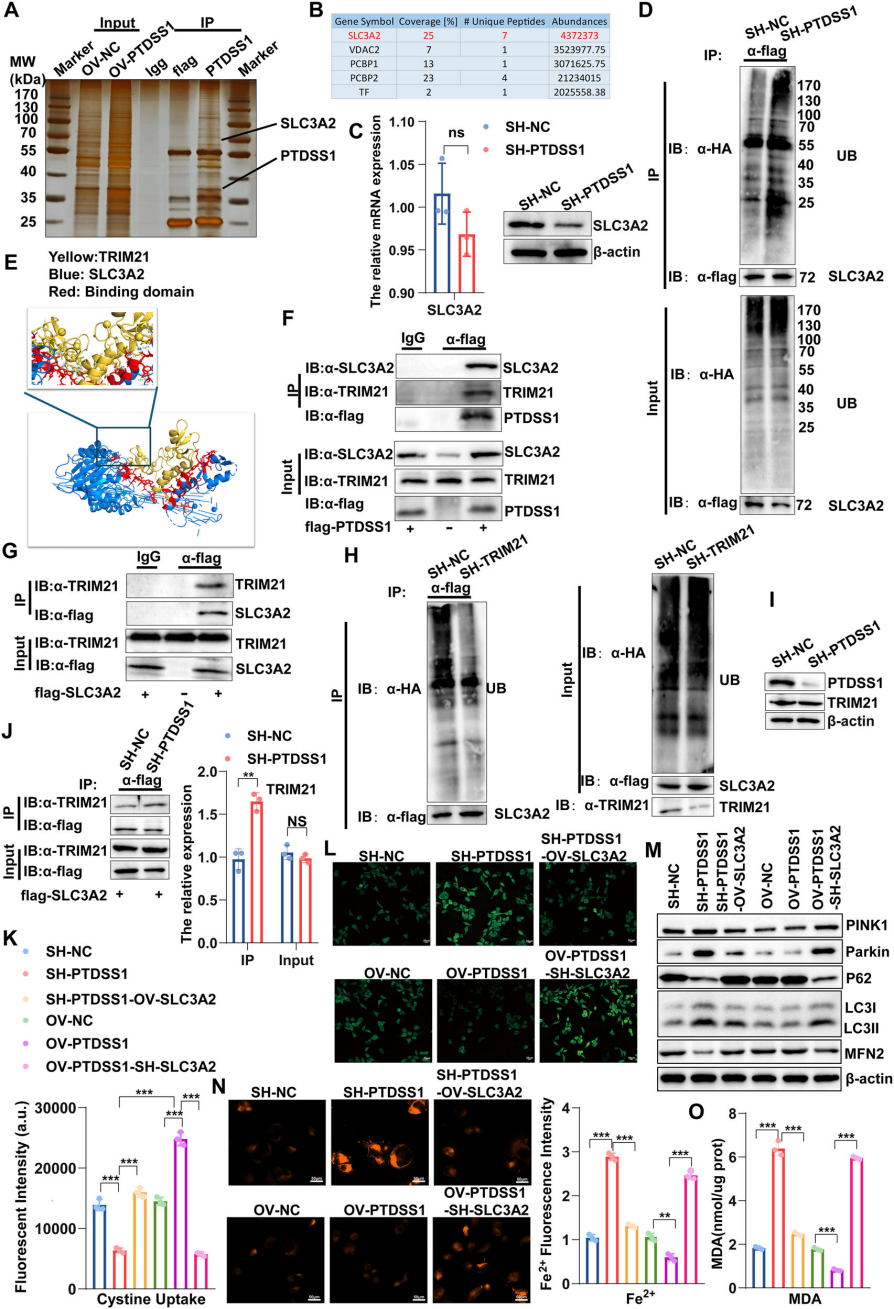
The knockdown of PTDSS1 regulates the generation of GSH by facilitating the combination of TRIM21 and SLC3A2. **A**, **B** Affinity purification and identification of PTDSS1-interacting proteins: Kyse30 cell lysates stably expressing Flag-PTDSS1 were purified using an anti-Flag affinity column, eluted products were separated by SDS-PAGE, visualized by silver staining, and protein bands were analyzed by mass spectrometry. **C** SLC3A2 expression in SH-PTDSS1 versus SH-NC groups assessed by RT-PCR and Western blot. **D** SLC3A2 ubiquitination levels in Kyse30^SH−NC^ versus Kyse30^SH−PTDSS1^ cells; MG132 (10 μM) was added 5 h before cell harvest. **E** Schematic showing specific binding sites between SLC3A2 and TRIM21 at the molecular level. **F**, **G** Interaction between PTDSS1 and SLC3A2, and between TRIM21 and SLC3A2, demonstrated by immunoprecipitation and immunoblotting in Kyse30 cells transfected with indicated plasmids. **H** SLC3A2 ubiquitination levels in Kyse30^SH−NC^ versus Kyse30^SH−TRIM21^ cells; MG132 (10 μM) was added 5 h before cell harvest. **I** PTDSS1 and TRIM21 protein expression in Kyse30^SH−NC^ versus Kyse30^SH−PTDSS1^ cells by Western blot. **J** TRIM21-SLC3A2 interaction in Kyse30^SH−NC^ versus Kyse30^SH−PTDSS1^ cells assessed by immunoprecipitation. **K** Cystine uptake in different treatment groups. **L** ROS levels observed by fluorescence microscopy. Scale bar: 20 μm. **M** Expression of key mitophagy proteins after various cell treatments by Western blot. **N** Intracellular Fe^2+^ levels in different treatment groups observed by fluorescence microscopy. Scale bar: 50 μm. **O** MDA content in different treatment groups. *P < 0.05, **P < 0.01, ***P < 0.001.

Through screening differentially expressed genes in the common pathways enriched in cell and tissue sequencing, we discovered that PTDSS1 significantly positively correlates with the key genes for GSH synthesis and ferroptosis (GCLM, GCLC, GPX4) (Fig. S6A, B). To determine how PTDSS1 regulates the generation of GSH, we transfected Kyse30 cells with flag-tagged PTDSS1, isolated immune complexes using Co-IP, separated them via SDS-PAGE, and performed silver staining for protein visualization. Mass spectrometry analysis was conducted using liquid chromatography-tandem mass spectrometry (LC-MS/MS) identified differentially expressed proteins that were enriched in the ferroptosis pathway and interacted with the SLC3A2 protein (Fig. S6C, D, Fig. 6A, B). SLC3A2 regulates XC-/GSH/GPX4 system and participate in the regulation of GSH synthesis to inhibit ferroptosis caused by lipid hydroperoxides accumulation [42, 43]. Although knockdown of PTDSS1 had no significant effect on the RNA level of SLC3A2, a significant decrease in SLC3A2 expression was observed at the protein level (Fig. 6C). We also observed that the ubiquitination level of SLC3A2 was conspicuously elevated in SH-PTDSS1 cells (Fig. 6D). Mass spectrometry analysis revealed that PTDSS1 could interact with the ubiquitinated protein TRIM21, and TRIM21 has a binding site with SLC3A2, suggesting that PTDSS1 may affect the protein stability of SLC3A2 by regulating the interaction between TRIM21 and SLC3A2 (Fig. S6E, Fig. 6E). We detected the binding of TRIM21 with PTDSS1 and SLC3A2 through co-immunoprecipitation experiments, verifying physical interaction between TRIM21 and these two proteins (Fig. 6F, G). Notably, after TRIM21 knockdown, the ubiquitination level of SLC3A2 was significantly reduced, suggesting that TRIM21 may act as a ubiquitination enzyme for SLC3A2 (Fig. 6H). Considering the interaction between PTDSS1 and the ubiquitination enzyme TRIM21 of SLC3A2, we hypothesized that the reduction in PTDSS1 abundance might promote the interaction between TRIM21 and SLC3A2, thereby leading to the ubiquitination and degradation of SLC3A2. Through co-immunoprecipitation experiments, we observed a significant increase in the interaction between SLC3A2 and TRIM21 in SH-PTDSS1 cells, while the total TRIM21 protein level in the cell lysate remained unchanged (Fig. 6I-J), supporting our hypothesis.

Our functional assays indicated that PTDSS1 activates key proteins (such as GCLM, GCLC, and GPX4) in the XC-/GSH/GPX4 system via SLC3A2-mediated cystine uptake, promoting GSH synthesis (Fig. 6K, Fig. S6F–H). We then investigated the role of SLC3A2 in mitophagy and ferroptosis. The results indicated that SLC3A2 was capable of effectively suppressing the intracellular ROS accumulation, the activation of the PINK1/Parkin/MFN2 mitophagy pathway, as well as the increase of Fe^2+^ accumulation and the content of MDA after knockdown of PTDSS1 (Fig. 6L–O). Subsequently, the results of the cell viability assay indicated that under the condition where PTDSS1 expression was inhibited, treatment with the ferroptosis inducer RSL3 significantly increased the mortality rate of tumor cells. Notably, either SLC3A2 overexpression or treatment with the ferroptosis inhibitor Liproxstatin-1 (LIP-1) rescued cells from this death phenotype (Fig. S6I).

Collectively, our findings demonstrate that the knockdown of PTDSS1 facilitates the binding of TRIM21 to SLC3A2, increasing SLC3A2 ubiquitination and suppressing the activation of the XC-/GSH/GPX4 system. This cascade activates the PINK1/Parkin/MFN2 mitophagy pathway and ultimately induces cellular apoptosis and ferroptosis.

### PTDSS1 inhibition combined with RSL3 treatment aggravates ferroptosis and apoptosis

**Fig. 7:**
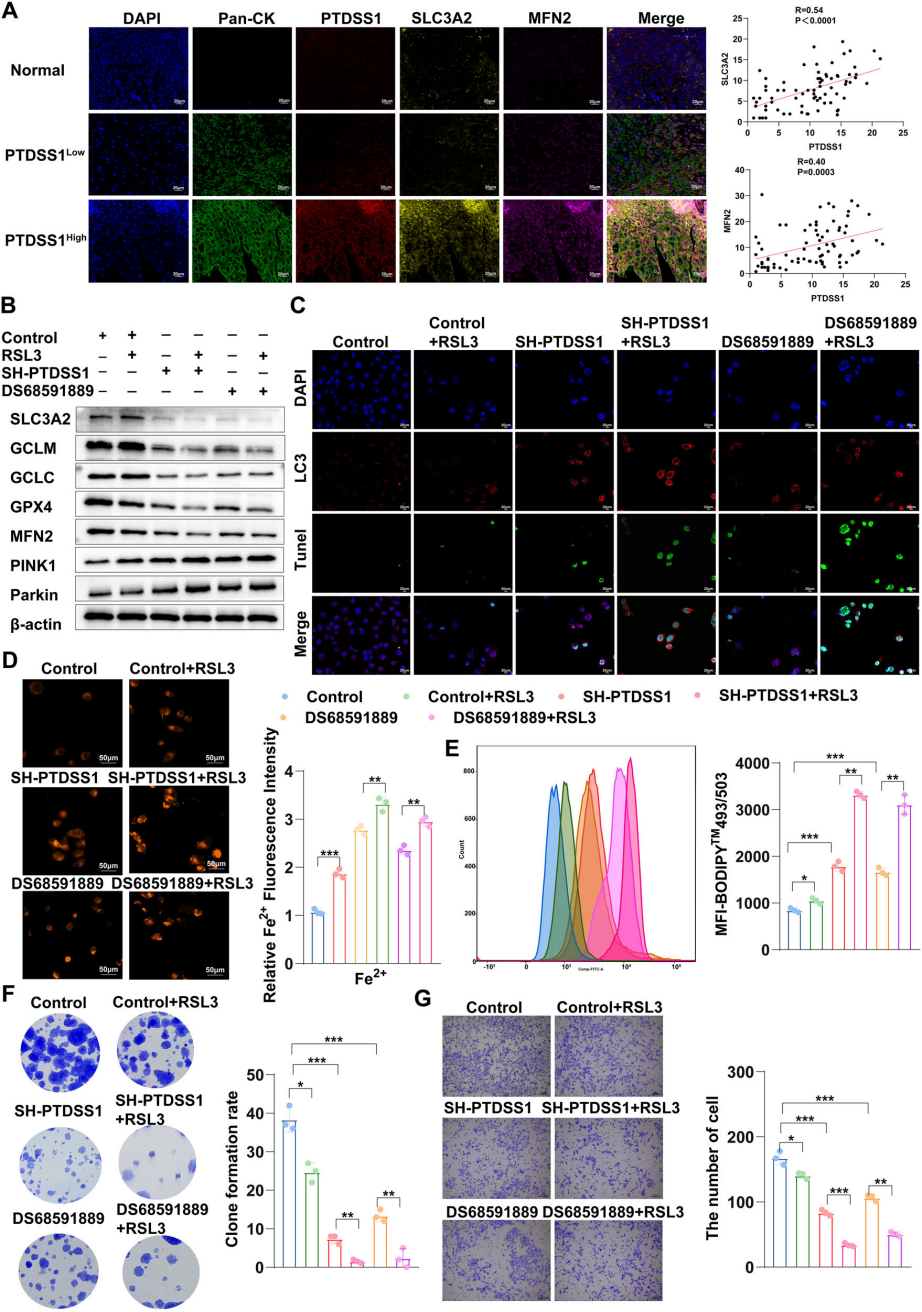
PTDSS1 inhibition combined with RSL3 treatment aggravates ferroptosis and apoptosis. **A** Multiplex immunofluorescence was employed to detect the expression of the key proteins regulated by PTDSS1 in tissue microarrays. Scale bar: 20 μm. **B** Effects of PTDSS1 inhibitor DS68591889 (100nΜ), SH-PTDSS1, and RSL3 (5 μΜ) combination on mitophagy and glutathione uptake proteins. **C** Mitophagy and apoptosis following combined treatment with PTDSS1 inhibitor DS68591889, SH-PTDSS1, and RSL3, assessed by cellular immunofluorescence. Scale bar: 20 μm. **D** Intracellular Fe^2+^ content after combined treatment with PTDSS1 inhibitor DS68591889, SH-PTDSS1, and RSL3. Scale bar: 50 μm. **E** Intracellular lipid peroxidation levels were detected by flow cytometry after the combination of PTDSS1 inhibitors DS68591889 and SH-PTDSS1 with RSL3. **F** Colony formation ability of cells was detected after the combination of PTDSS1 inhibitors DS68591889 and SH-PTDSS1 with RSL3. **G** Cell invasion ability of cells was detected after the combination of PTDSS1 inhibitors DS68591889, SH-PTDSS1 and RSL3. Scale bar: 100 μm. *P < 0.05, **P < 0.01, ***P < 0.001.

We employed Multiplex immunofluorescence technique to analyze the correlations between PTDSS1 and SLC3A2 as well as MFN2 proteins in tissue microarrays. The outcomes demonstrated a remarkable positive correlation between PTDSS1 and these two proteins (Fig. 7A), validating our previous findings at the tissue level. We further evaluated the anti-tumor effect of the combination of PTDSS1 inhibitor DS68591889 and ferroptosis activator RSL3, and compared it with the PTDSS1 knockdown group. At the protein level, PTDSS1 inhibitors significantly suppressed the expression of ferroptosis suppressor proteins (including SLC3A2, GCLM, GCLC, and GPX4), while RSL3 only had a significant effect on GPX4 and had a minor impact on other proteins. However, in the process of mitophagy, RSL3 enhanced mitophagy activation caused by PTDSS1 inhibition, evidenced by upregulated PINK1 and Parkin expression and downregulated MFN2 protein. We noted that PTDSS1 knockdown produced comparable effects to PTDSS1 inhibitor treatment (Fig. 7B). Using cell immunofluorescence and TUNEL assay, we observed more intuitively that RSL3 aggravated mitophagy and apoptosis of tumor cells induced by PTDSS1 inhibition. Simultaneously, RSL3 significantly increased intracellular Fe^2+^ content, promoted lipid peroxidation, and thereby inhibited the clonal formation and invasion capabilities of tumor cells (Fig. 7C–G). These results suggest that RSL3 potentiates mitophagy caused by PTDSS1 interference by inhibiting the expression of GPX4 protein, thereby enhancing ferroptosis and apoptosis to achieve a stronger anti-tumor effect.

### The anti-tumor effect of the combination of PTDSS1 inhibitor and RSL3 was examined in vivo experiments

**Fig. 8:**
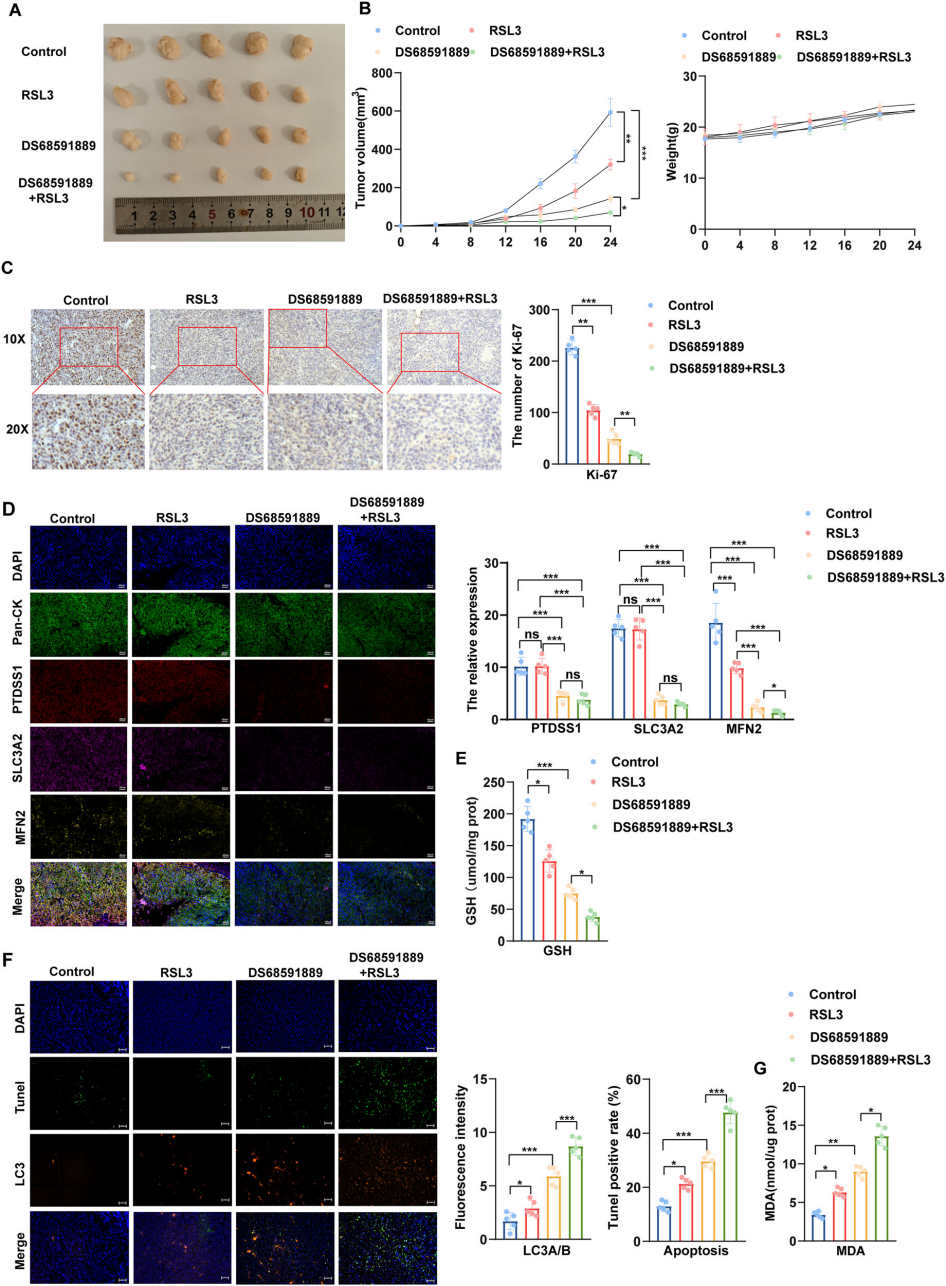
The anti-tumor effect of the combination of PTDSS1 inhibitor and RSL3 was examined in vivo experiments. **A**, **B** Tumor growth inhibition by PTDSS1 inhibitor DS68591889, ferroptosis activator RSL3, or their combination in xenograft models. **C** Tumor proliferation assessed by Ki-67 immunohistochemical staining after treatment with PTDSS1 inhibitor DS68591889, RSL3, or their combination. Scale bar: 50 μm. **D** Expression of PTDSS1, SLC3A2, and MFN2 proteins after treatment with PTDSS1 inhibitor DS68591889, RSL3, or their combination, detected by multiplex immunofluorescence. Scale bar: 40 μm. **E** Intracellular GSH levels after treatment with PTDSS1 inhibitor DS68591889, RSL3, or their combination. **F** Mitophagy (LC3) and apoptosis (TUNEL) after treatment with PTDSS1 inhibitor DS68591889, RSL3, or their combination, assessed by tissue immunofluorescence. Scale bar: 50 μm. **G** Intracellular MDA levels after treatment with PTDSS1 inhibitor DS68591889, RSL3, or their combination. *P < 0.05, **P < 0.01, ***P < 0.001.

A subcutaneous xenograft tumor model was established in nude mice using Kyse30 cells with high expression of PTDSS1. One week post-implantation, the experimental groups were treated with RSL3 (5 mg/kg/d), PTDSS1 inhibitor (DS68591889, 30 mg/kg/d), or a combination of both. After 17 days of treatment, the mice were sacrificed and the tumors were removed. The tumor growth curve showed that both RSL3 and PTDSS1 inhibitor alone could significantly inhibit tumor growth, while the combination of the two exhibited a better anti-tumor effect. No significant weight loss or death was observed during the treatment period (Fig. 8A, B), suggesting good tolerability of these drugs.

Mechanistic studies revealed that the combination of RSL3 and DS68591889 significantly inhibited the proliferation activity of tumor cells, as indicated by the reduction in Ki-67 levels (Fig. 8C). Multiplex immunofluorescence analysis verified the regulatory effect of this combined treatment on protein expression: the results showed that DS68591889 significantly reduced the expression of SLC3A2 and MFN2 proteins, while RSL3 only inhibited the expression of MFN2 protein (Fig. 8D). Subsequently, we measured the intracellular GSH content and found that RSL3 could intensify the inhibitory effect of DS68591889 on GSH synthesis, thus exacerbating cellular oxidative stress (Fig. 8E). Our analysis of mitophagy, apoptosis, and MDA levels demonstrated that RSL3 enhanced DS68591889 -induced mitophagy in tumor cells, resulting in increased apoptosis and ferroptosis, strengthening DS68591889 ‘s anti-tumor effect (Fig. 8F, G). Together, these findings indicate that RSL3 exacerbates PTDSS1 inhibitor-induced mitophagy by inhibiting the generation of GSH, thereby promoting ferroptosis and apoptosis in tumor cells.

## Discussion

**Fig. 9:**
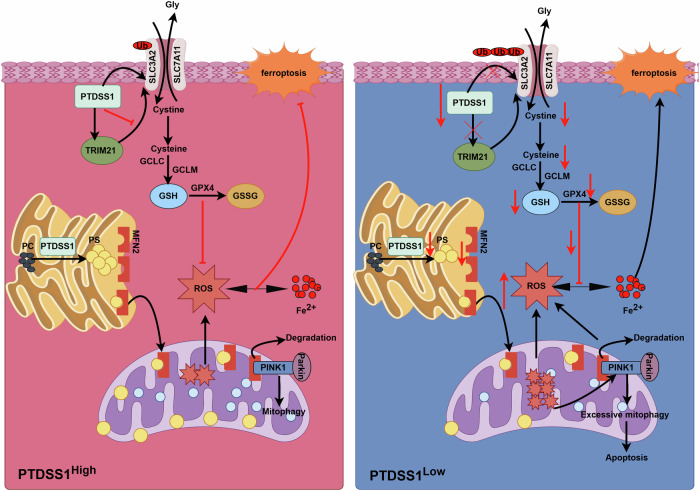
Mechanism by which targeting PTDSS1 induces mitophagy and ferroptosis in ESCC. Schematic model showing how PTDSS1 inhibition triggers SLC3A2 ubiquitination via TRIM21, leading to suppressed GSH synthesis, increased oxidative stress, activation of PINK1/Parkin/MFN2-mediated mitophagy, and ultimately resulting in tumor cell ferroptosis and apoptosis.

In this study, our comprehensive single-cell transcriptomic analysis revealed a PTDSS1-high malignant epithelial subpopulation with significant enrichment in ferroptosis and mitophagy regulatory pathways. We found that the high expression of PTDSS1 is significantly correlated with poor prognosis in ESCC patients. PTDSS1 is a crucial enzyme for synthesizing phosphatidylserine, which is synthesized in the ER and transported to the PM and mitochondria via multiple pathways to maintain cellular homeostasis [15]. Our findings demonstrate, for the first time, that PTDSS1 in ESCC exhibits dual regulatory functions: modulating GSH synthesis via SLC3A2 in the plasma membrane while simultaneously governing MFN2 protein distribution in mitochondria. Targeted inhibition of PTDSS1 expression results in increased ubiquitination of SLC3A2 and inhibition of GSH synthesis, thereby inducing oxidative stress in tumor cells, activating the PINK1/Parkin/MFN2 mitophagy pathway, and ultimately promoting tumor cell apoptosis and ferroptosis (Fig. 9). Our research suggests that targeting PTDSS1 in combination with ferroptosis induction could offer a novel therapeutic approach for the treatment of ESCC.

To our knowledge, no previous studies have reported the specific mechanism through which PTDSS1 regulates mitophagy and ferroptosis. Through combined analysis of transcriptomics and metabolomics, we discovered that knockdown of PTDSS1 significantly suppressed the generation of GSH. GSH constitutes a significant component of the cellular antioxidant system [44]. Within tumor cells, elevated GSH levels are indispensable for eliminating excessive ROS and detoxifying exogenous substances, thereby rendering it a potential target for cancer therapy [32]. GSH depletion can lead to the accumulation of intracellular ROS, which directly impacts mitophagy and ferroptosis [45].

Mitophagy is a selective autophagy process specifically targeting mitochondria, which removes aged and damaged mitochondria through the specific sequestration and phagocytosis of mitochondria in lysosomes, thereby limiting the production of mtROS [46]. The relationship between ROS and mitophagy in cancer remains controversial [47]. Moderate ROS levels can promote mitophagy through multiple pathways, thereby promoting tumor progression. For instance, ROS can induce mitophagy by activating the PI3K/AKT signaling pathway, thereby promoting the proliferation and invasion of hepatocellular carcinoma (HCC) [48]. ROS can also trigger PINK1-dependent mitophagy to eliminate damaged mitochondria and protect cells from apoptosis [49]. Conversely, severe oxidative stress or persistent mitochondrial damage can induce excessive pathological mitophagy activation, further elevating ROS levels [35]. and triggering cell death and tissue damage [49]. For instance, propionate-induced ROS and the imbalance of redox homeostasis cause mitophagy, thereby enhancing ferroptosis and apoptosis [24].

In our study, PTDSS1 knockdown inhibited GSH synthesis, resulting in ROS accumulation, mitochondrial dysfunction, and increased mitophagy—as evidenced by electron microscopy, PINK1/Parkin signaling pathway activation, and elevated intracellular MDA content. Consequently, tumor cell proliferation and survival were inhibited. Importantly either GSH supplementation or PINK1 knockdown in PTDSS1-depleted cells significantly reversed these effects, indicating that PTDSS1 knockdown induces oxidative stress by inhibiting GSH synthesis, activating PINK1/Parkin-mediated mitophagy, and promoting tumor cell ferroptosis and apoptosis.

Furthermore, it has been reported that PTDSS1, during endoplasmic reticulum synthesis, can specifically bind to PS and transport it to the outer mitochondrial membrane [16]. This process plays a crucial role in maintaining the structure and function of mitochondria [15]. Our metabolomics analysis revealed that knockdown of PTDSS1 led to a decrease in PS content, thereby promoting the transport of MFN2 from the plasma membrane to the outer mitochondrial membrane. As a specific substrate of PINK1/Parkin, MFN2 undergoes ubiquitination and degradation by Parkin after PINK1 recognition. When exogenous GSH is supplemented or PINK1 is knocked down, only the expression level of MFN2 in the outer mitochondrial membrane can be restored. Therefore, targeting PTDSS1 induces GSH depletion, leading to oxidative stress, activates the PINK1/Parkin/MFN2 mitophagy pathway, and promotes tumor ferroptosis and apoptosis.

Ferroptosis, driven by ROS and Fe^2+^ accumulation leading to lipid peroxidation, is modulated by various cellular metabolic and signaling pathways. Evidence for the role of mitochondria in regulating ferroptosis is growing increasingly, yet the related research findings are still controversial [50]. In pancreatic cancer cells, targeting the oncogene Myoferlin triggers mitophagy and promotes ferroptosis [51]. In melanoma cells, inhibiting mitochondrial complex I induces mitophagy-dependent ROS increases, leading to ferroptosis [27]. Similarly, targeting SIRT3 sensitizes glioblastoma to ferroptosis by promoting mitophagy and inhibiting SLC7A11 [26]. These studies collectively report demonstrate that mitophagy has a promoting effect on ferroptosis. Consistent with these findings, knockdown of PTDSS1 leads to enhanced mitophagy, which can cause accumulation of intracellular ROS and Fe^2+^, promoting ferroptosis. In oxidative stress-induced mitophagy, the level of ROS can be increased, further inducing mitophagy to form a positive feedback regulation [22]. Mitophagy can increase the lysosomal iron content of mitochondrial iron-sulfur clusters. Peroxidation and permeabilization of the lysosomal membrane lead to iron leakage, causing iron accumulation [52, 53]. Therefore, the oxidative stress induced by GSH depletion resulting from PTDSS1 knockdown may be the key mechanism driving excessive mitophagy and subsequent ferroptosis in tumor cells.

The system Xc- (comprising SLC7A11 and SLC3A2) mediates the exchange of cystine and glutamate across the plasma membrane, facilitating GSH synthesis [54]. Our proteomic analysis revealed interactions between PTDSS1, SLC3A2 and TRIM21. PTDSS1 knockdown strengthens SLC3A2-TRIM21 interaction, resulting in SLC3A2 ubiquitination and degradation, which subsequently inhibits cystine uptake and reduces glutathione synthesis enzyme expression (GCLM, GCLC, and GPX4), ultimately suppressing GSH synthesis. When we added the ferroptosis inducer RSL3 to inhibit GPX4 activity, it enhanced the anti-tumor effect of PTDSS1 knockdown, evidenced by significantly increased mitophagy, ferroptosis, and apoptosis. It is worth noting that SLC3A2 overexpression largely rescued GSH depletion-induced mitophagy and ferroptosis caused by PTDSS1 knockdown. However, the regulation of mitochondrial structure and function by PS in mitochondria is not yet fully understood, possibly due to differences in mitochondrial structure and function between tumor cells and normal cells.

In conclusion, our research demonstrates that targeting PTDSS1 induces oxidative stress by suppressing SLC3A2-mediated GSH synthesis and activating PINK1/Parkin/MFN2-dependent mitophagy, thereby promoting tumor cell apoptosis and ferroptosis while inhibiting tumor progression. We further found that the ferroptosis inducer RSL3 and the PTDSS1 inhibitor DS68591889 have a synergistic effect, and animal experiments verified the safety of this synergy. Therefore, we propose PTDSS1 as a potential biomarker for cancer diagnosis and prognosis, and suggest developing targeted treatment strategies for patients with high PTDSS1 expression to improve clinical outcomes in ESCC.

## Supplementary information

Supplementary-Figure

Supplementary Table

Original WB images

Reproducibility checklist

## Data Availability

All data supporting this study are present in the paper and Supplementary Materials. Online public data can be acquired from the TCGA database (http://cancergenome.nih.gov/), Gene Expression Omnibus (GEO) datasets (https://www.ncbi.nlm.nih.gov/geo/).
