# Supplementary material for: Targeting PTDSS1 to modulate GSH synthesis triggers mitophagy and induces ferroptosis in esophageal squamous cell carcinoma cells
Source: Cell Death Dis. 2026 Apr 23;17(1):538. doi: 10.1038/s41419-026-08702-4 (PMC13236975; doi:10.1038/s41419-026-08702-4)
Supplement: Supplementary file 1 — Supplementary-Figure [file 41419_2026_8702_MOESM1_ESM.pdf]

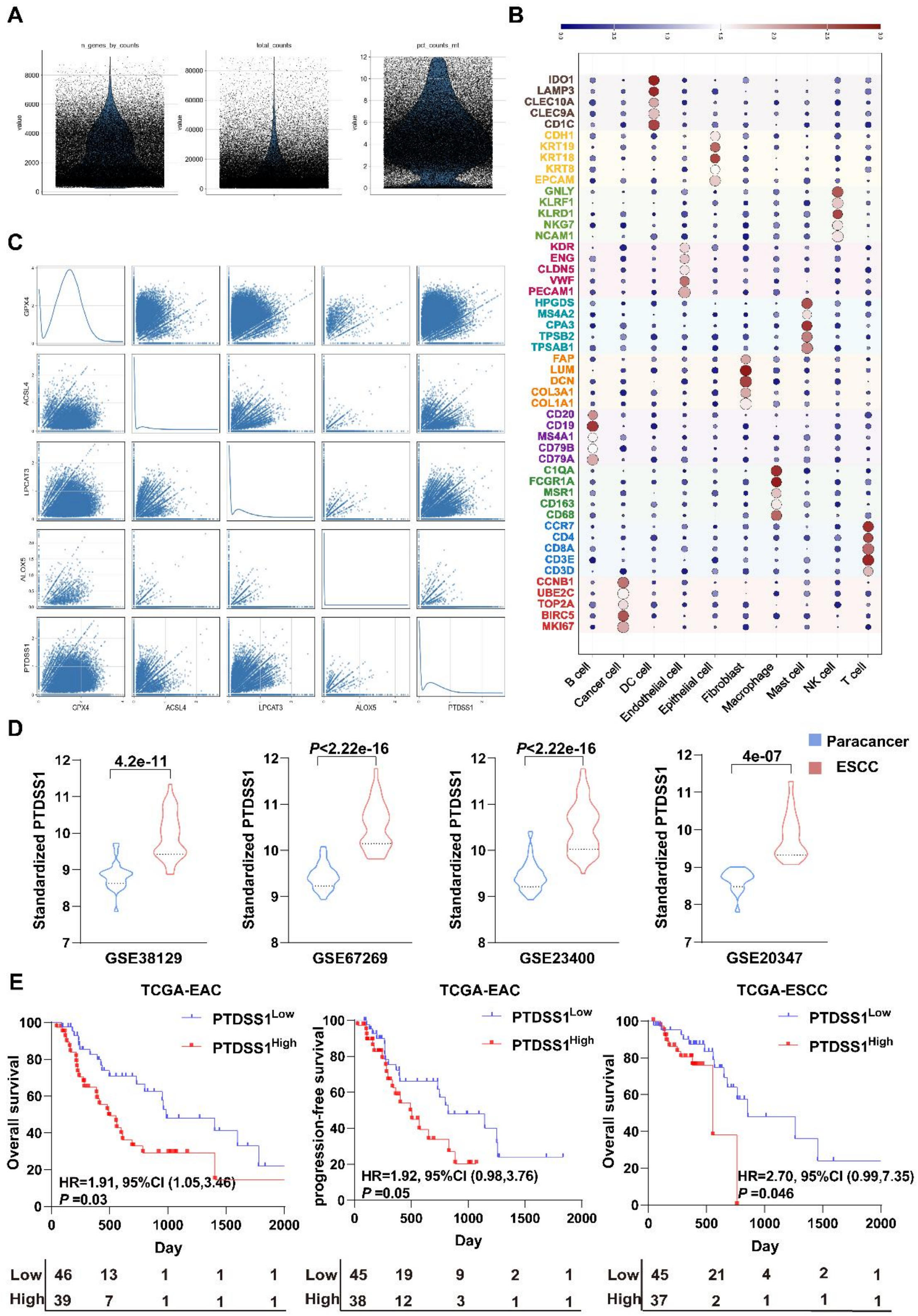

Figure S1. PTDSS1 expression correlates with malignant progression and poor prognosis in ESCC.

(A) Violin plots depicting quality control metrics (gene count, UMI count, and mitochondrial gene percentage) used for cell filtering across integrated ESCC datasets.

(B) Heatmap showing marker gene expression patterns across identified cell types, confirming distinct transcriptional profiles for each cell population. (C) Correlation matrix illustrating relationships between PTDSS1 and key ferroptosis-related genes (GPX4, ACSL4, LPCAT3, ALOX5) across malignant cells, highlighting PTDSS1's potential role in ferroptosis regulation. (D) The expression of PTDSS1 in four GEO databases. (E) Kaplan-Meier analysis showing high PTDSS1 expression predicts poor prognosis in esophageal squamous cell carcinoma (ESCC), with less pronounced effect in esophageal adenocarcinoma (ESA). **P<0.05, **P<0.01, ***P<0.001.*

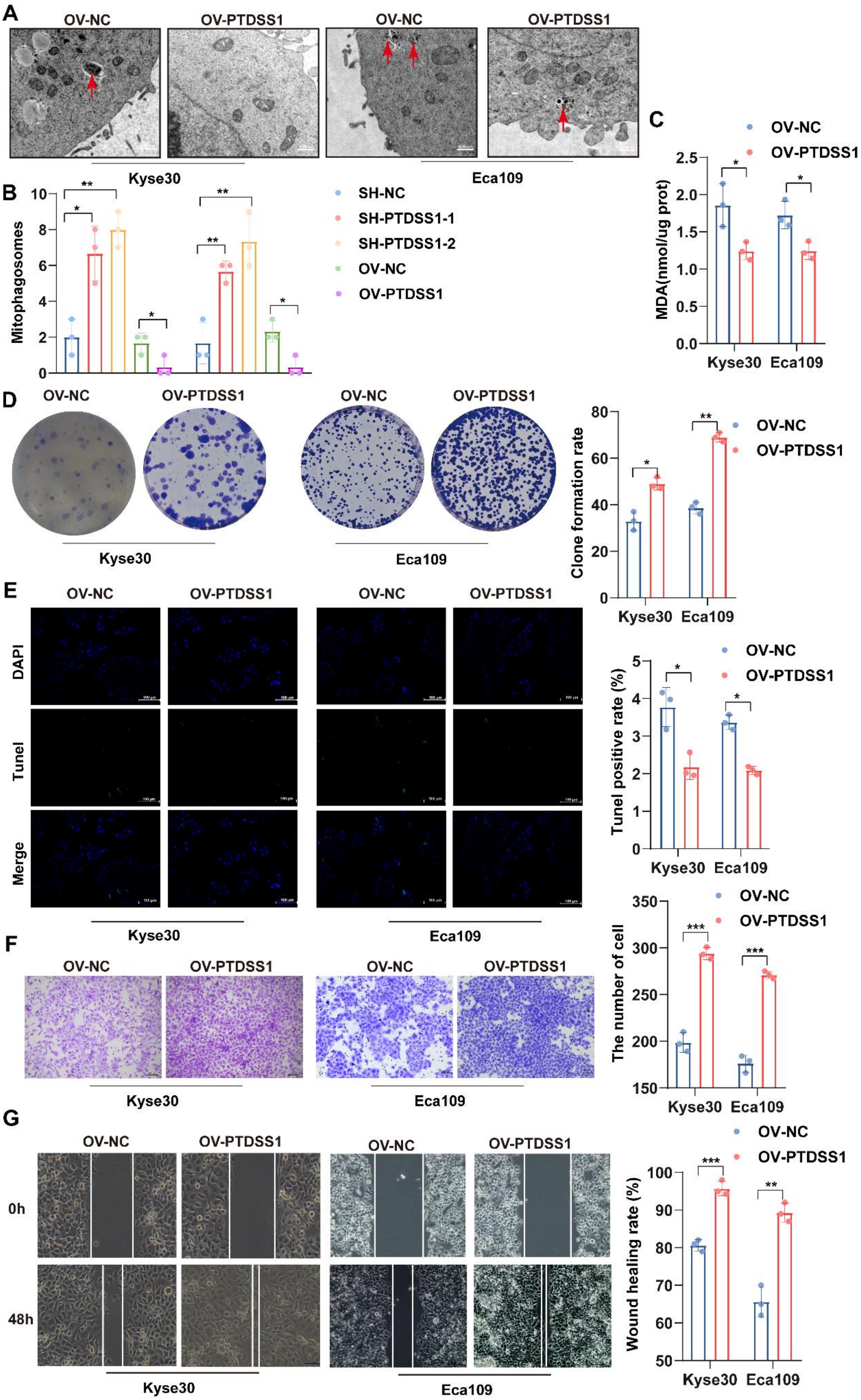

Figure S2. Overexpression of PTDSS1 promotes the malignant progression of

ESCC.

(A) Mitochondrial ultrastructural changes observed by TEM. Ultrastructural analysis revealed that mitophagy was characterized by mitochondrial swelling, cristae disorganization, and eventual engulfment within autophagosomes, followed by lysosomal degradation. Red arrows indicate mitophagy. Scale bar: 500 nm. (B) Quantitative analysis of mitophagy. (C) Intracellular MDA concentration. (D) Colony formation assay results in PTDSS1-overexpressing cells versus controls. (E) TUNEL assay detection of apoptosis in PTDSS1-overexpressing cells versus controls. (F) Representative images of trans-well invasion assays in PTDSS1-overexpressing cells versus controls. (G) Representative images of wound healing assays in PTDSS1-overexpressing cells versus controls. Scale bar: 100 μm. **$P<0.05$, **$P<0.01$, ***$P<0.001$.*

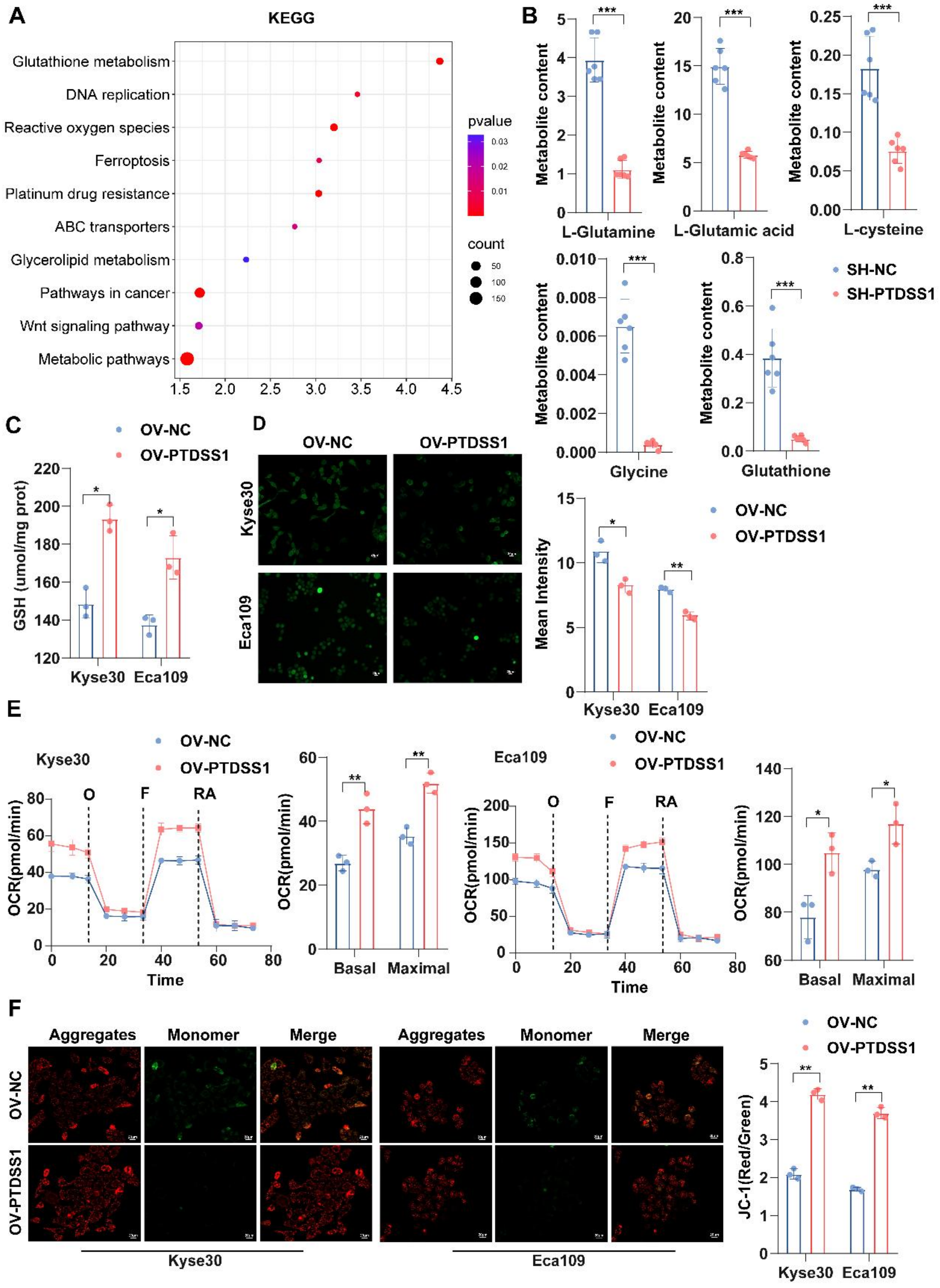

Figure S3. PTDSS1 overexpression enhances mitochondrial function in ESCC cells.

(A) KEGG pathway enrichment analysis of differentially expressed genes in ESCC dataset from TCGA. (B) Metabolomics analysis showing levels of metabolites in the intracellular glutamine-glutamate pathway. (C) Intracellular GSH content in Eca109 cells with PTDSS1 modulation. (D) Intracellular ROS levels visualized by

fluorescence microscopy in Eca109 cells. (E) Oxygen consumption rate (OCR) measurements in Eca109 cells with PTDSS1 modulation. (F) Mitochondrial membrane potential (MMP) assessment in Eca109 cells with PTDSS1 modulation. Scale bar: 20 μm. *$P<0.05$, **$P<0.01$, ***$P<0.001$.

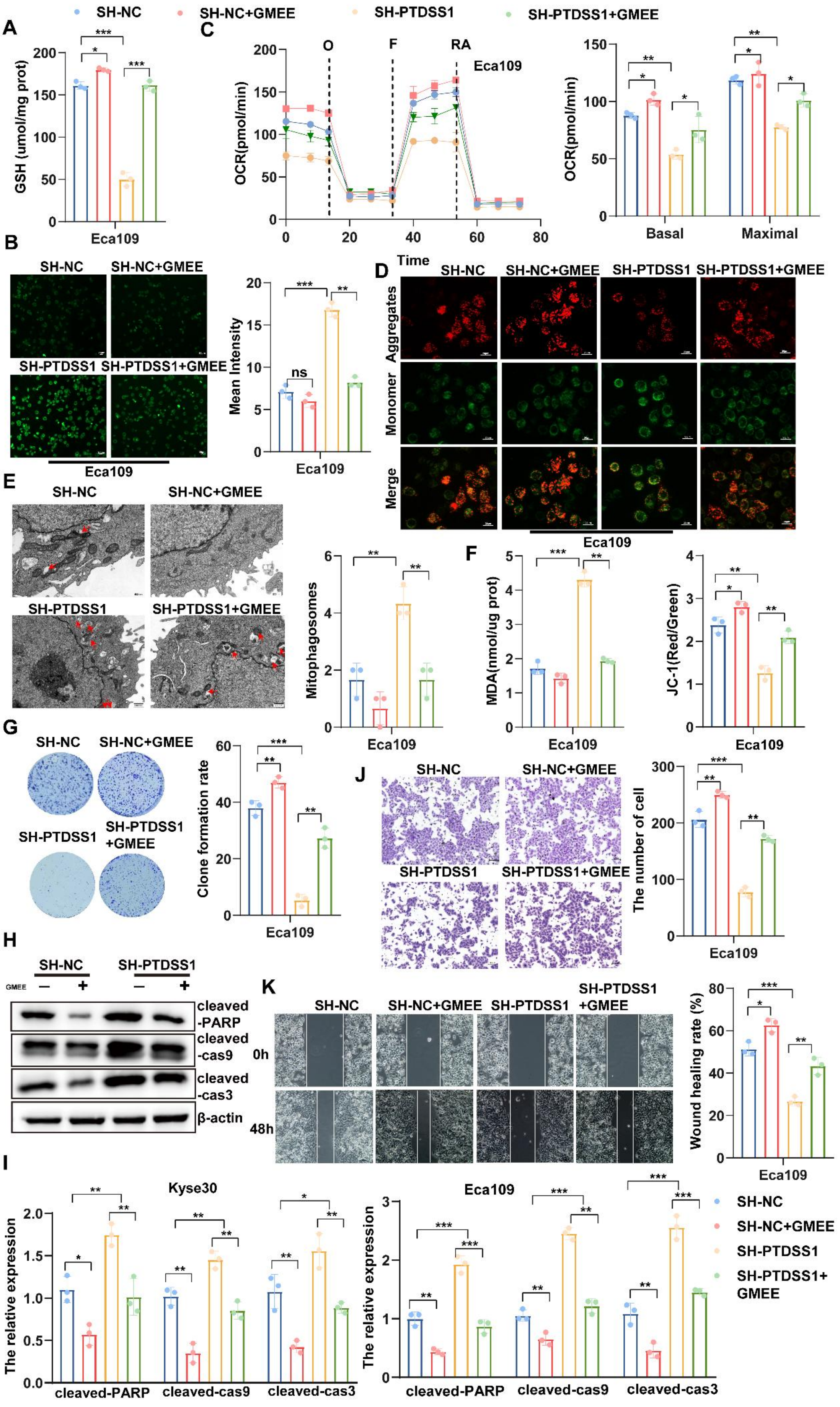
SH-NC
SH-NC+GMEE
SH-PTDSS1
SH-PTDSS1+GMEE
A
GSH (umol/mg prot)
Eca109
C
OCR(pmol/min)
O
F
RA
Eca109
Time
Basal
Maximal
B
Mean Intensity
ns
D
Aggregates
Monomer
Merge
E
Mitophagosomes
F
MDA(nmol/ug prot)
JC-1(Red/Green)
G
Clone formation rate
J
The number of cell
H
GMEE
cleaved
-PARP
cleaved
-cas9
cleaved
-cas3
β-actin
K
0h
48h
Wound healing rate (%)
I
Kyse30
The relative expression
cleaved-PARP
cleaved-cas9
cleaved-cas3
SH-PTDSS1+
GMEE

Figure S4. The GSH depletion caused by knockdown of PTDSS1 can induce mitophagy in Eca109 cell lines.

(A) Intracellular GSH content in Eca109 SH-PTDSS1 and control cells with or without glutathione monoethyl ester (GMEE, 10 mM) supplementation. (B) Intracellular ROS levels after GMEE supplementation. Scale bar: 20 μm. (C) OCR in SH-PTDSS1 and control cells after GMEE treatment. (D) MMP after GMEE treatment. Scale bar: 20 μm. (E) Ultrastructural changes in mitochondria visualized by TEM after GMEE treatment, with red arrows indicating mitophagy. Scale bar: 500 nm. (F) MDA concentration changes after GMEE supplementation in Eca109 cells. (G) Colony formation capacity after GMEE treatment in Eca109 cells. (H) Western blot analysis of apoptotic protein expression after GMEE treatment in Eca109 cells. (I) Quantitative analysis results. (J)Trans-well invasion assays after GMEE treatment in Eca109 cells. (K)Wound healing assays after GMEE treatment in Eca109 cells. Scale bar: 100 μm. **P<0.05, **P<0.01, ***P<0.001.*

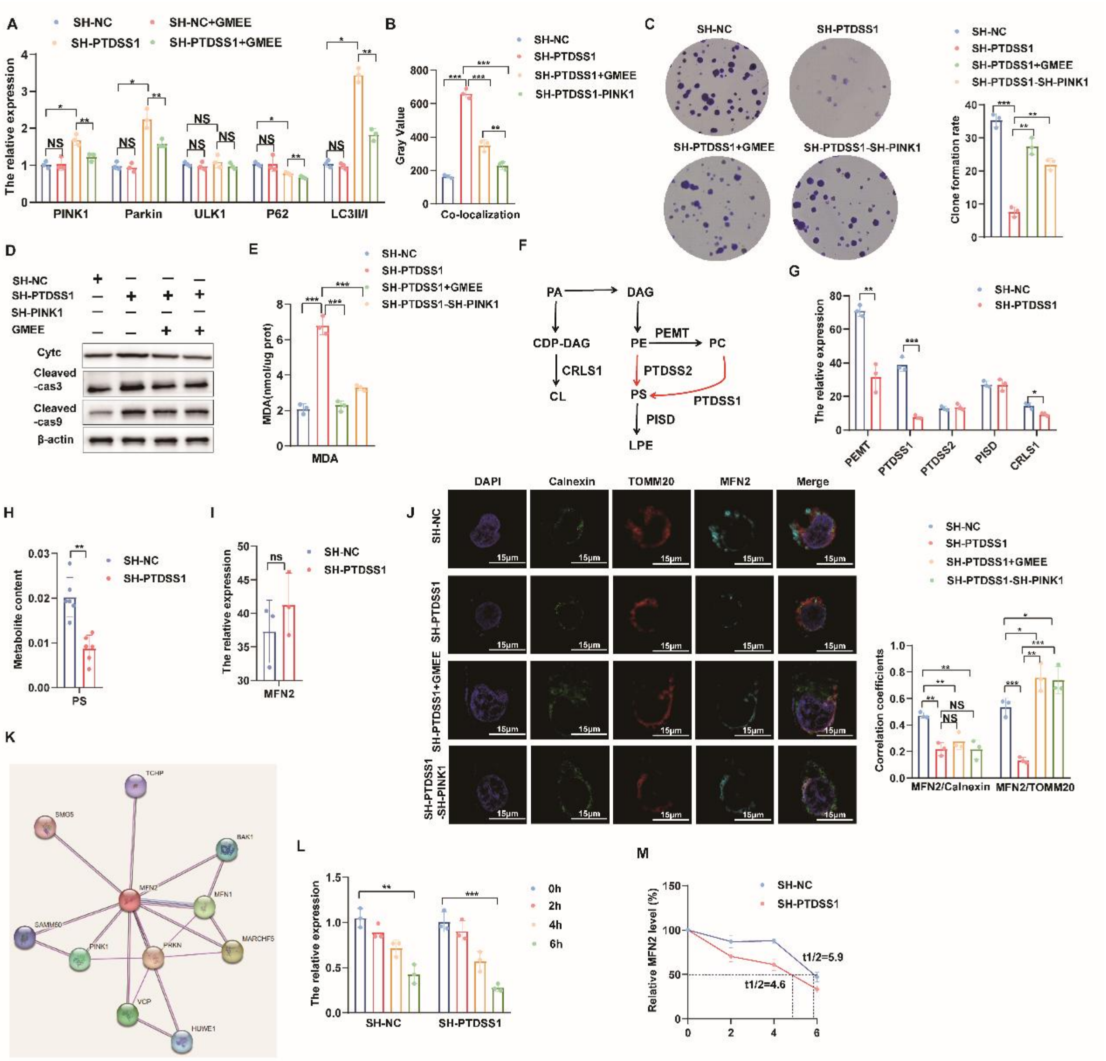

Figure S5. The depletion of GSH resulting from the knockdown of PTDSS1 can

induce mitophagy via the PINK1/Parkin/MFN2 pathway to promote ferroptosis and apoptosis.

(A) Quantitative analysis results for Figure 5B. (B) Fluorescence quantification data of Figure 5C. (C) Colony formation capacity in Kyse30 cells after GMEE treatment or PINK1 knockdown. (D) Western blot analysis of apoptotic protein expression after GMEE treatment or PINK1 knockdown in Kyse30 cells. (E) Intracellular MDA concentration in Kyse30$^{SH\text{-}PTDSS1}$ cells after GMEE treatment or PINK1 knockdown. (F) Schematic of phosphatidylserine metabolism. (G) RNA expression of key genes in phosphatidylserine metabolic pathway. (H) Phosphatidylserine content measured by metabolomics. (I) MFN2 RNA expression from transcriptomic analysis. (J) The results of through triple-label immunofluorescence co-localization (Calnexin for ER, TOMM20 for mitochondria, and MFN2). (K) MFN2 protein interaction network predicted using STRING database (https://cn.string-db.org/). (L) Quantitative analysis results for Figure 5L. (M) Cycloheximide (CHX) chase assay for MFN2 in SH-NC or SH-PTDSS1 transfected Kyse30 cells treated with CHX (500 μg/ml) for the indicated time points.

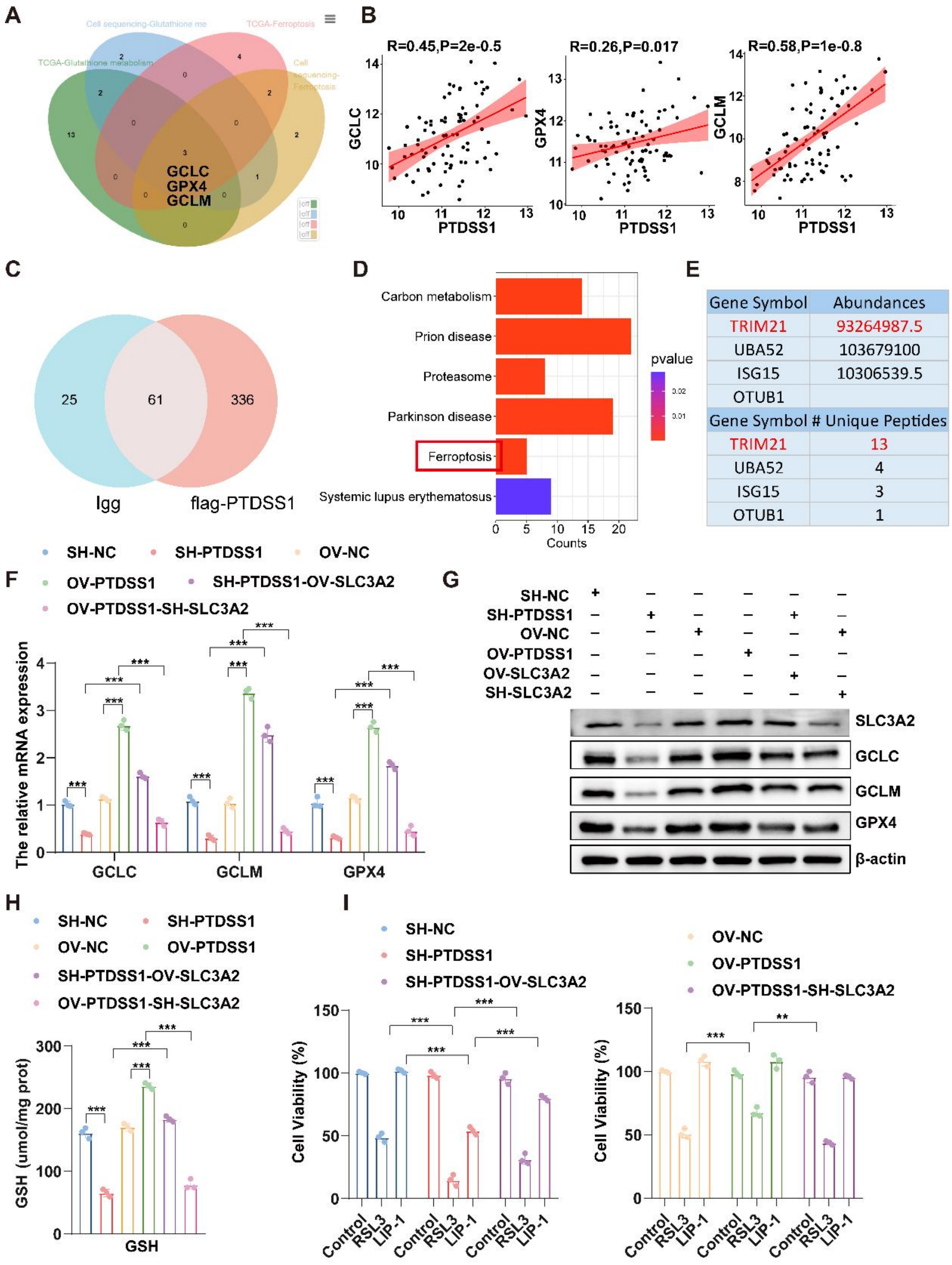

Figure S6. The knockdown of PTDSS1 regulates the generation of GSH by facilitating the combination of TRIM21 and SLC3A2.

(A) Venn diagram showing common differentially expressed genes in glutathione metabolism and ferroptosis pathways between TCGA and cell transcriptomes. (B) Correlation analysis between PTDSS1 expression and three key differentially expressed genes (GCLM, GCLC, and GPX4) in TCGA ESCC database. (C)

Proteomic analysis of PTDSS1-interacting proteins, with IgG as negative control. (D) KEGG pathway enrichment analysis of differentially expressed proteins. (E) Identification of ubiquitinated proteins interacting with PTDSS1. (F-G) RNA and protein expression of GCLM, GCLC, and GPX4 following PTDSS1 and SLC3A2 knockdown. (H) Intracellular GSH content after PTDSS1 and SLC3A2 knockdown. (I) Cell viability assessment after treatment with ferroptosis activator (RSL3) and ferroptosis inhibitor (Liproxstatin-1/LIP-1) in PTDSS1 and SLC3A2 knockdown cells.
