# Supplementary material for: Targeting PTDSS1 to modulate GSH synthesis triggers mitophagy and induces ferroptosis in esophageal squamous cell carcinoma cells
Source: Cell Death Dis. 2026 Apr 23;17(1):538. doi: 10.1038/s41419-026-08702-4 (PMC13236975; doi:10.1038/s41419-026-08702-4)
Supplement: Supplementary file 2 — Supplementary Table [file 41419_2026_8702_MOESM2_ESM.docx]

**Table S1. Antibody information**

**IHC/M-IHC antibody**

| **Antibody** | **Concentration** | **manufacturers** |
| --- | --- | --- |
| **PTDSS1** | **1:200** | **Abcam (R)** |
| **Pan-CK** | **1:500** | **Abcam (R)** |
| **SLC3A2** | **1:200** | **Proteintech (R)** |
| **MFN2** | **1:200** | **Abcam (R)** |
| **LC3** | **1:200** | **CST (R)** |
| **DAPI** |  | **Merck** |

**WB antibody**

| **Antibody** | **Concentration** | **manufacturers** |
| --- | --- | --- |
| **PTDSS1** | **1:1000** | **Abcam (R)** |
| **β-actin** | **1:1000** | **Proteintech (R)** |
| **Cleaved-PARP** | **1:1000** | **Proteintech (R)** |
| **Cleaved-cas9** | **1:500** | **Proteintech (R)** |
| **Cleaved-cas3** | **1:1000** | **Proteintech (R)** |
| **PINK1** | **1:1000** | **Proteintech (R)** |
| **Parkin** | **1:1000** | **Abcam (M)** |
| **ULK1** | **1:1000** | **Proteintech (R)** |
| **LC3** | **1:1000** | **CST (R)** |
| **MFN2** | **1:1000** | **Abcam (R)** |
| **P62** | **1:1000** | **Proteintech (R)** |
| **TOMM20** | **1:1000** | **Abclonal (R)** |
| **Ub** | **1:1000** | **CST (R)** |
| **Igg** | **1:50** | **Thermo (R)** |
| **flag** | **1:50** | **CST (M)** |
| **SLC3A2** | **1:1000** | **Proteintech (R)** |
| **TRIM21** | **1:1000** | **Proteintech (R)** |
| **GCLM** | **1:1000** | **Abcam (R)** |
| **GCLC** | **1:1000** | **Proteintech (R)** |
| **GPX4** | **1:1000** | **Proteintech (R)** |

**Table S2. Primer sequences for RT-PCR**

| **Gene** | **Forward primer (5' to 3')** | **Reverse primer (5' to 3')** |
| --- | --- | --- |
| **PTDSS1** | **AGACCTACTCGGAGTGTGAAGATGG** | **CCTGGAAGAATGGCTTTCGTTGTTG** |
| **SLC3A2** | **TGAATGAGTTAGAGCCCGAGA** | **GTCTTCCGCCACCTTGATCTT** |
| **GCLC** | **GGGGTGACGAGGTGGAGTA** | **GTTGGGGTTTGTCCTCTCCC** |
| **GCLM** | **AGGAGCTTCGGGACTGTATCC** | **GGGACATGGTGCATTCCAAAA** |
| **GPX4** | **ATGGTTAACCTGGACAAGTACC** | **GACGAGCTGAGTGTAGTTTACT** |
| **GAPDH** | **AGACAGCCGCATCTTCTTGT** | **CTTGCCGTGGGTAGAGTCAT** |
