# Supplementary material for: Targeting PTDSS1 to modulate GSH synthesis triggers mitophagy and induces ferroptosis in esophageal squamous cell carcinoma cells
Source: Cell Death Dis. 2026 Apr 23;17(1):538. doi: 10.1038/s41419-026-08702-4 (PMC13236975; doi:10.1038/s41419-026-08702-4)
Supplement: Supplementary file 3 — Original WB images [file 41419_2026_8702_MOESM3_ESM.docx]

**Figure1**


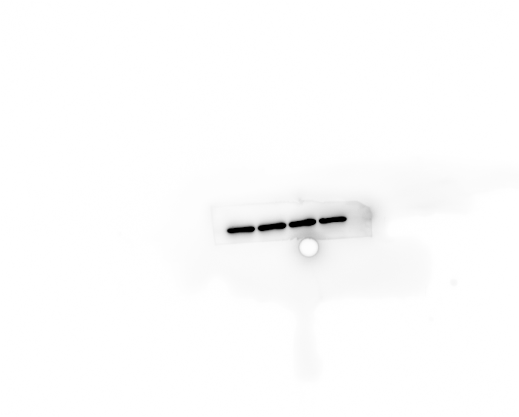

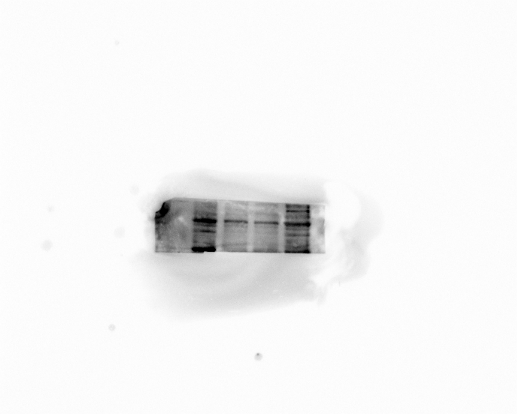


PTDSS1 β-actin


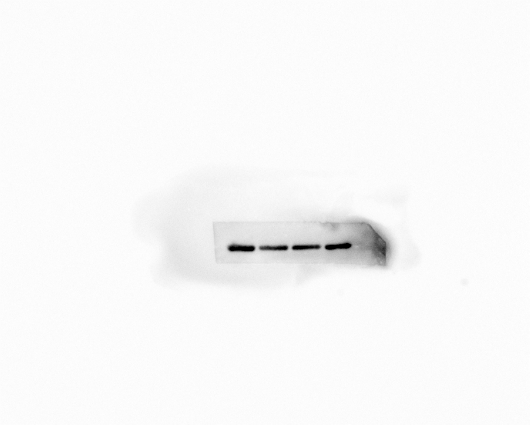

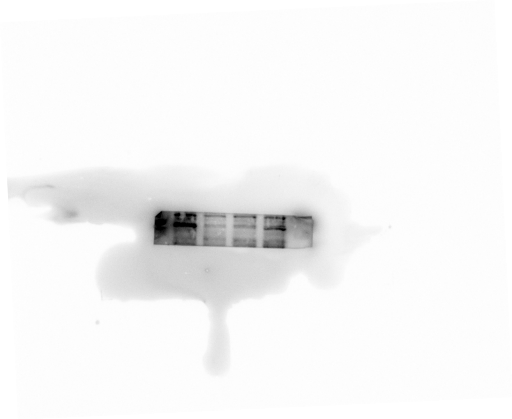


PTDSS1 β-actin

**Figure 4**


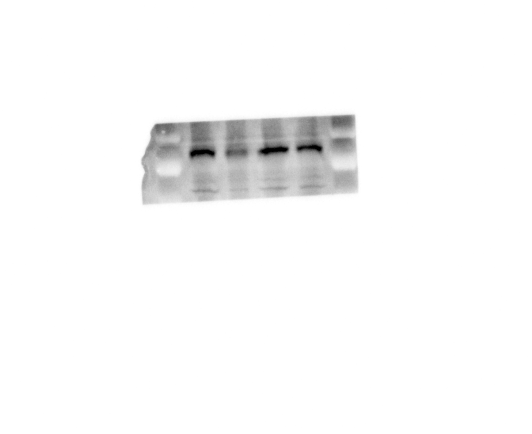

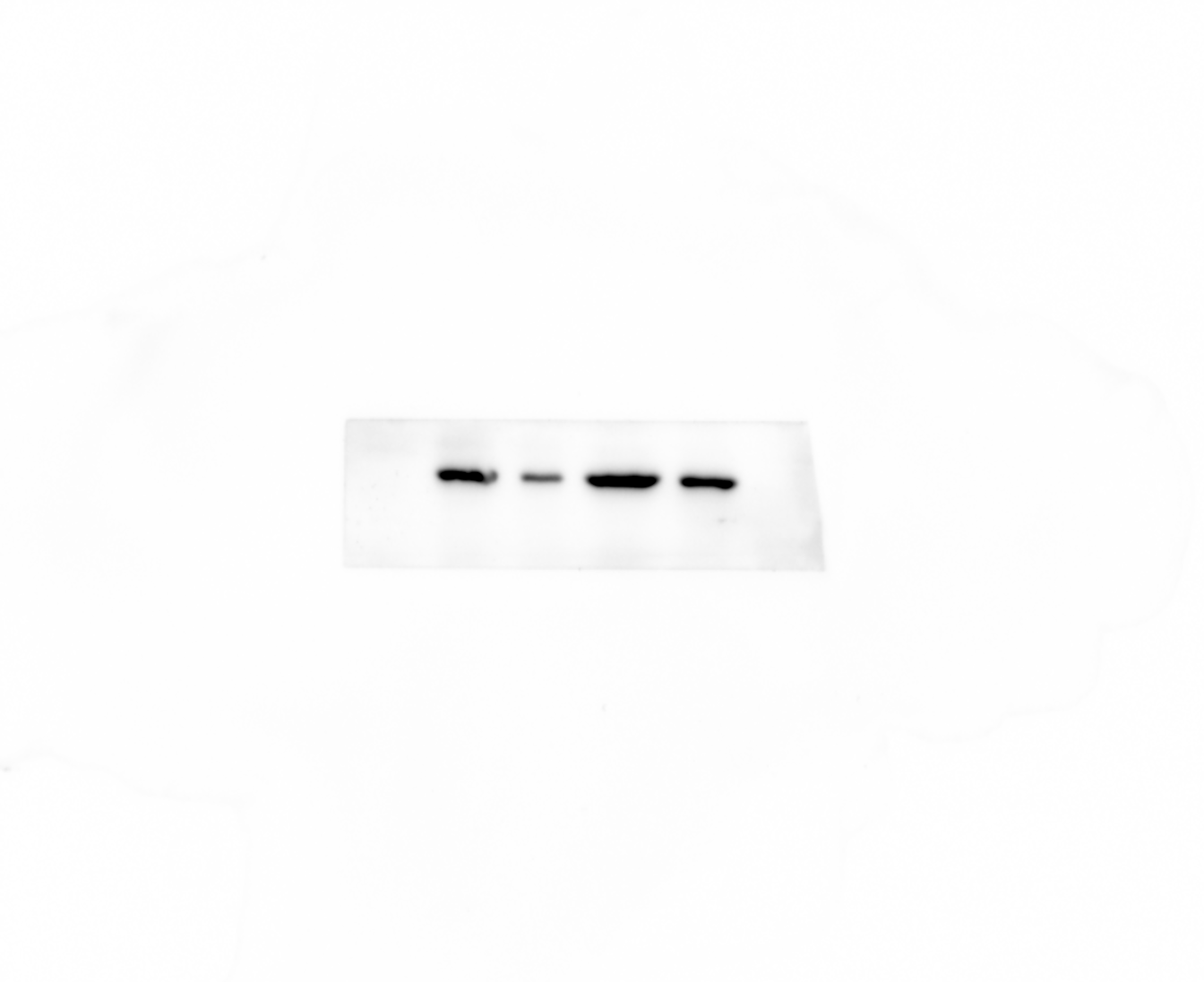


Cleaved-PARP Cleaved-cas9


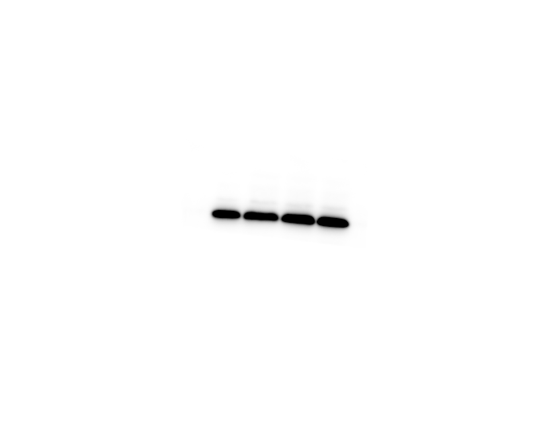

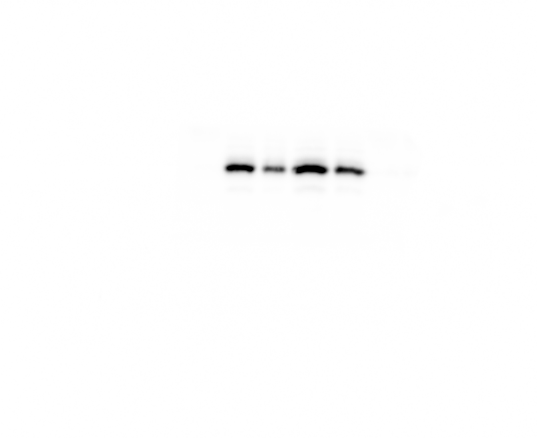


Cleaved-cas3 β-actin

**Figure S4**


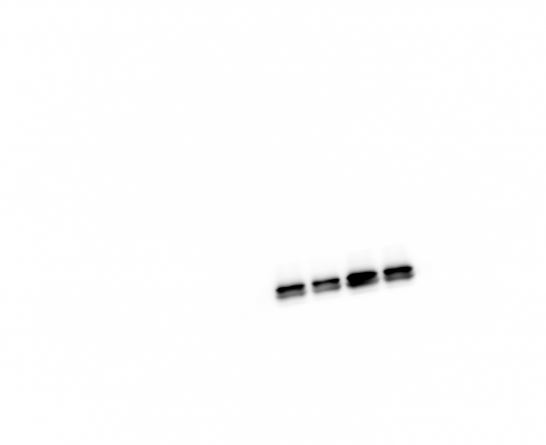

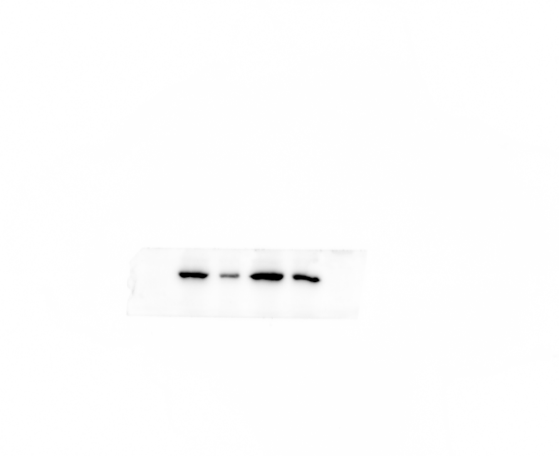



 Cleaved-PARP Cleaved-cas9


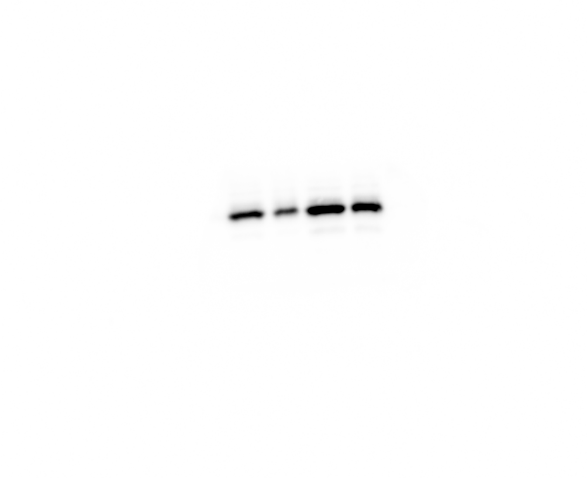
 β-actin

Cleaved-cas3

Figure 5


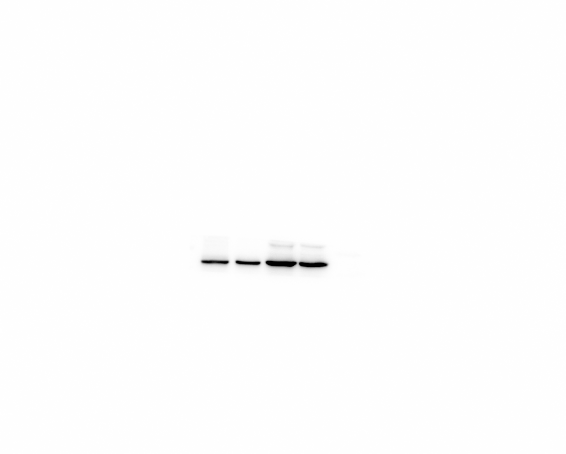

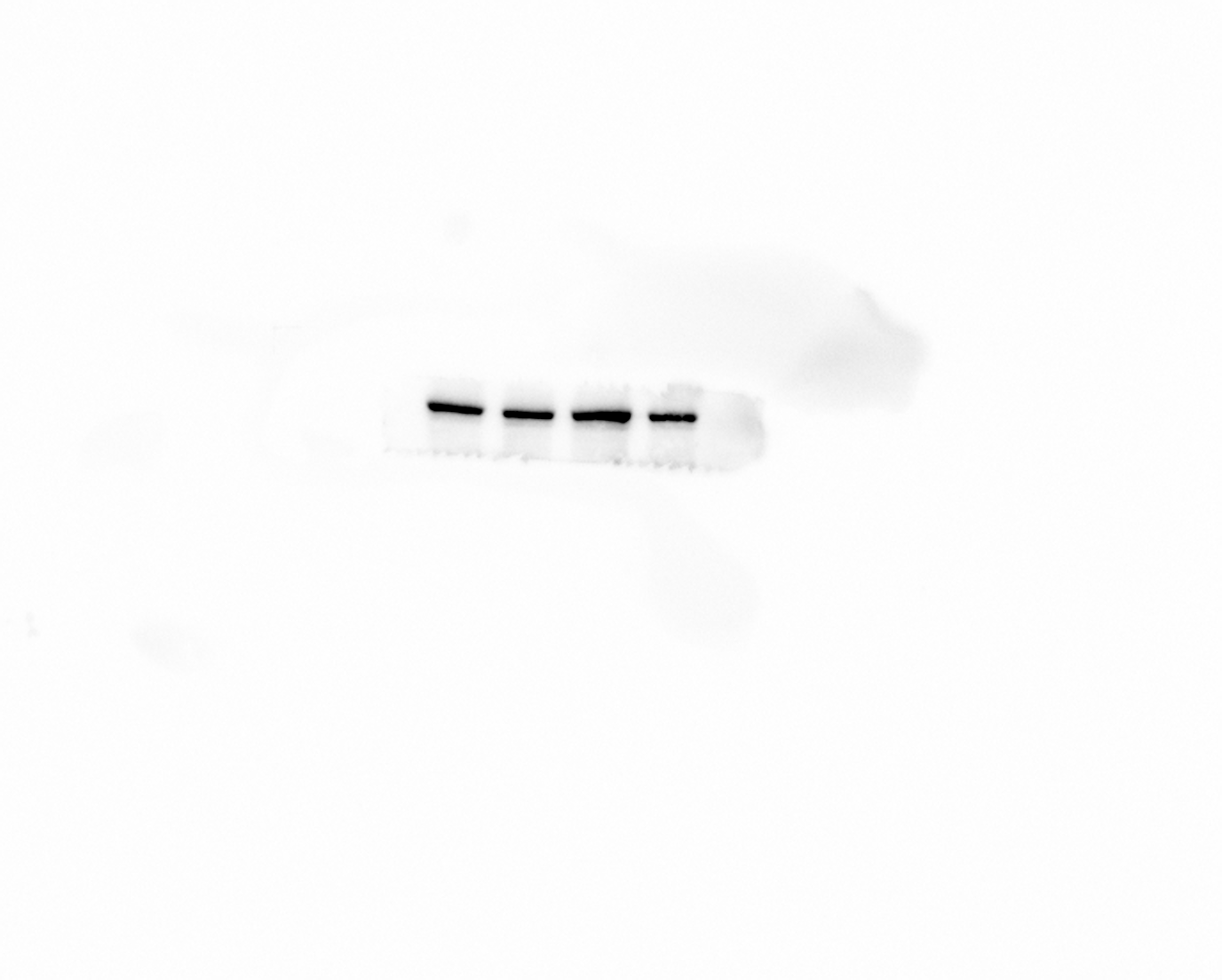


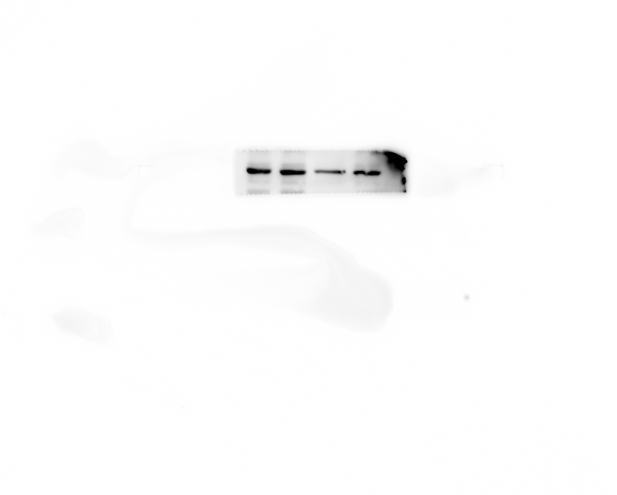

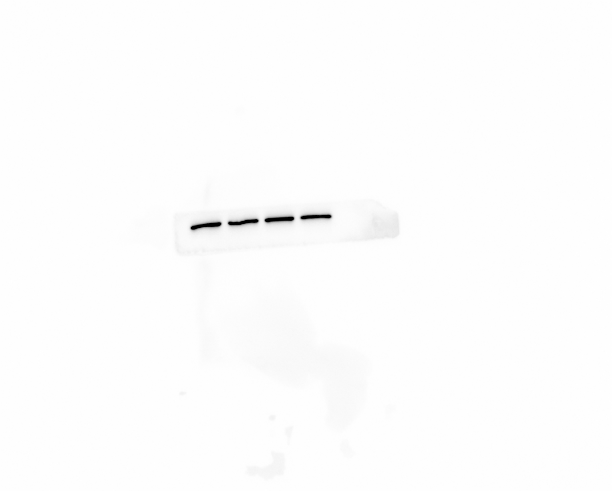
PINK1 Park

ULK1 P62


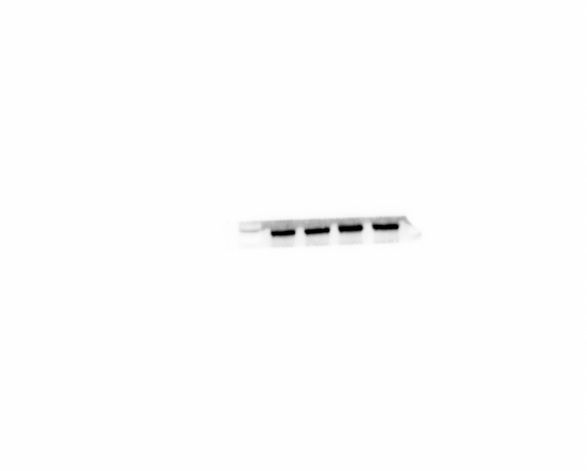

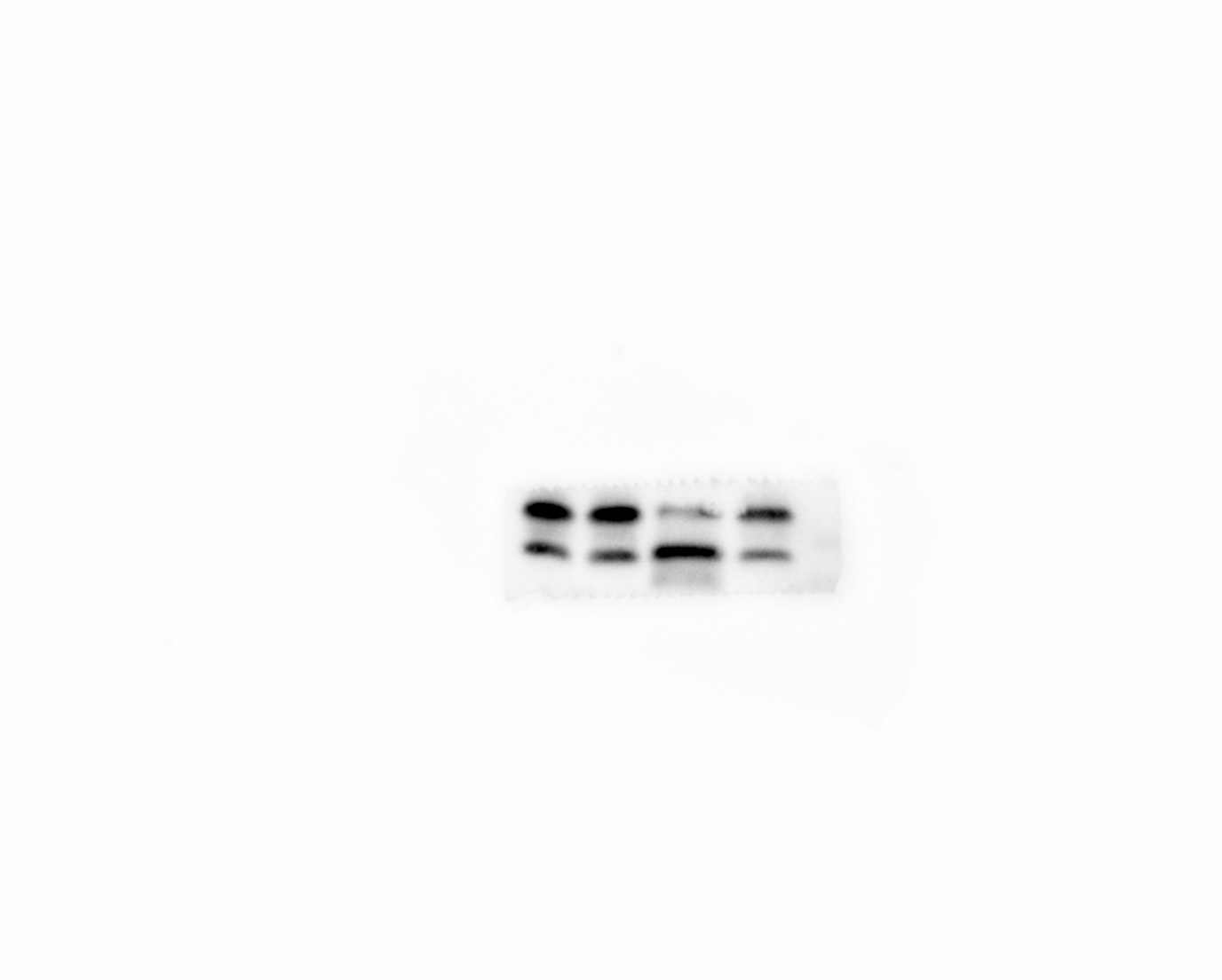


LC3I/II β-actin


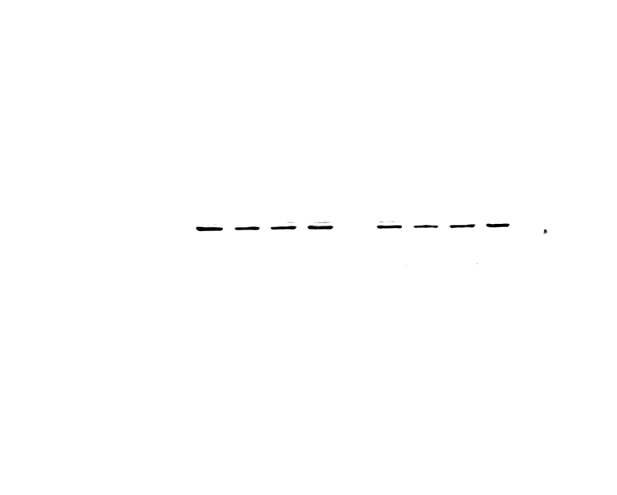

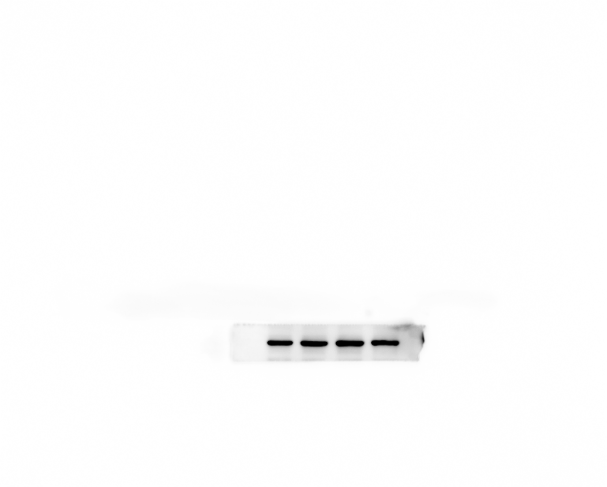

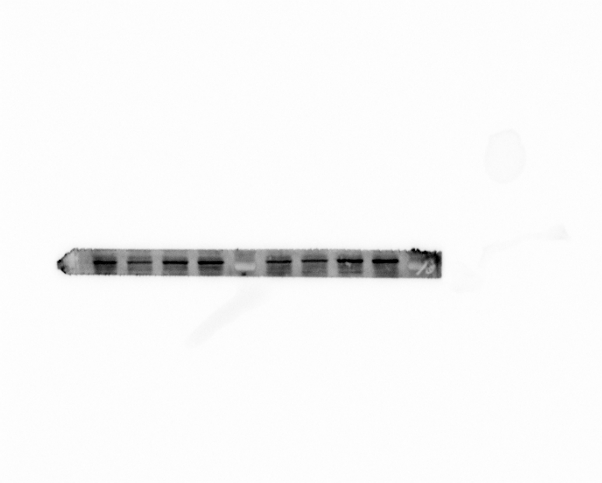
MFN2 β-actin


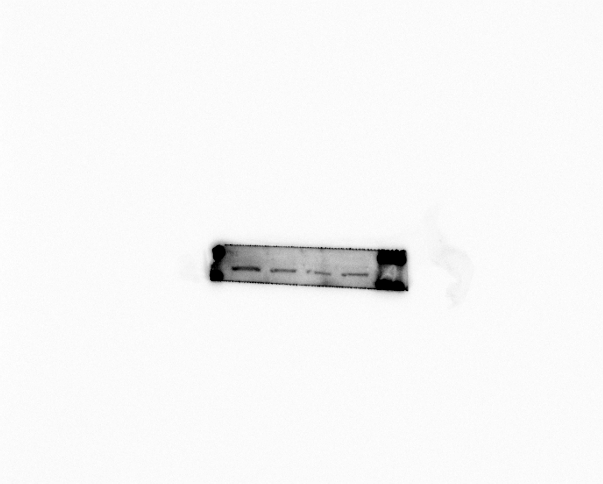
MFN2 Na/K-ATP


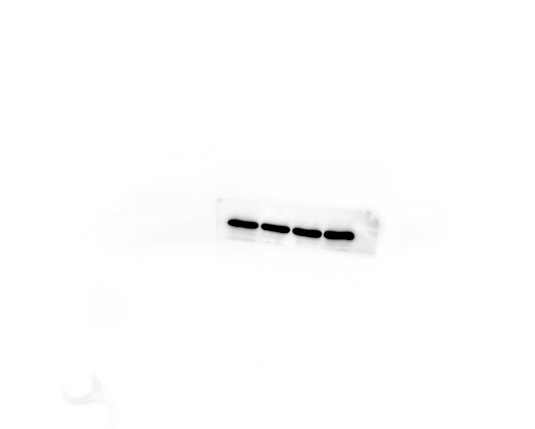

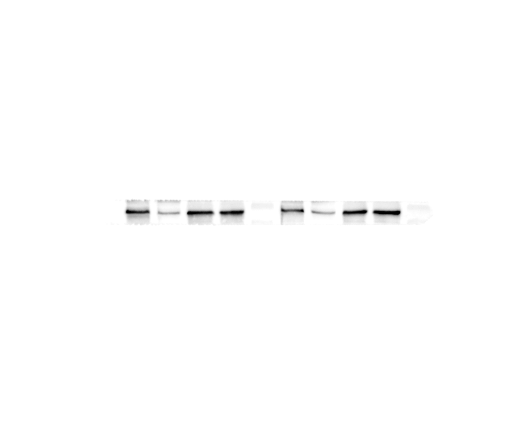


MFN2 TOMO20


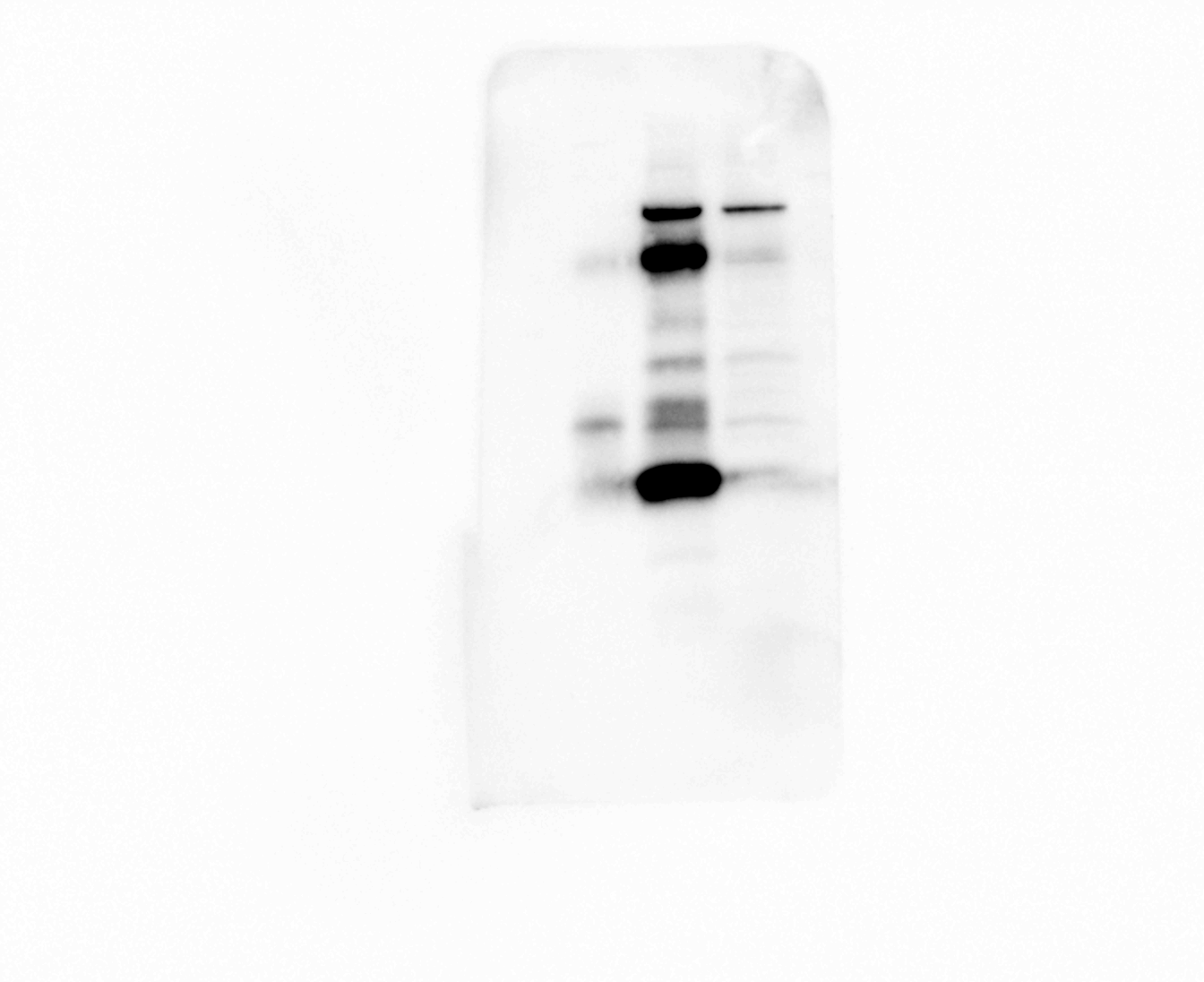

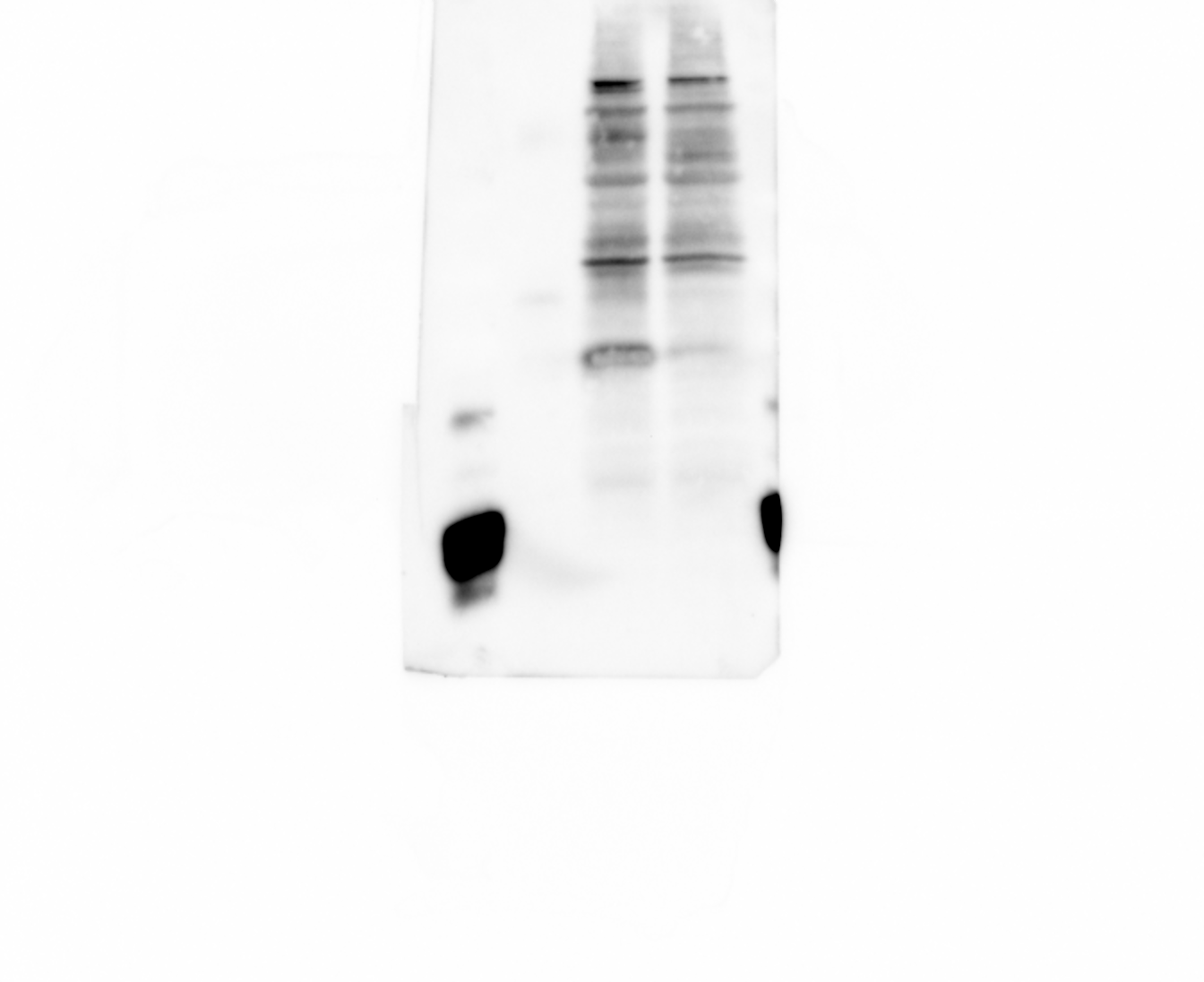


MFN2 PINK1


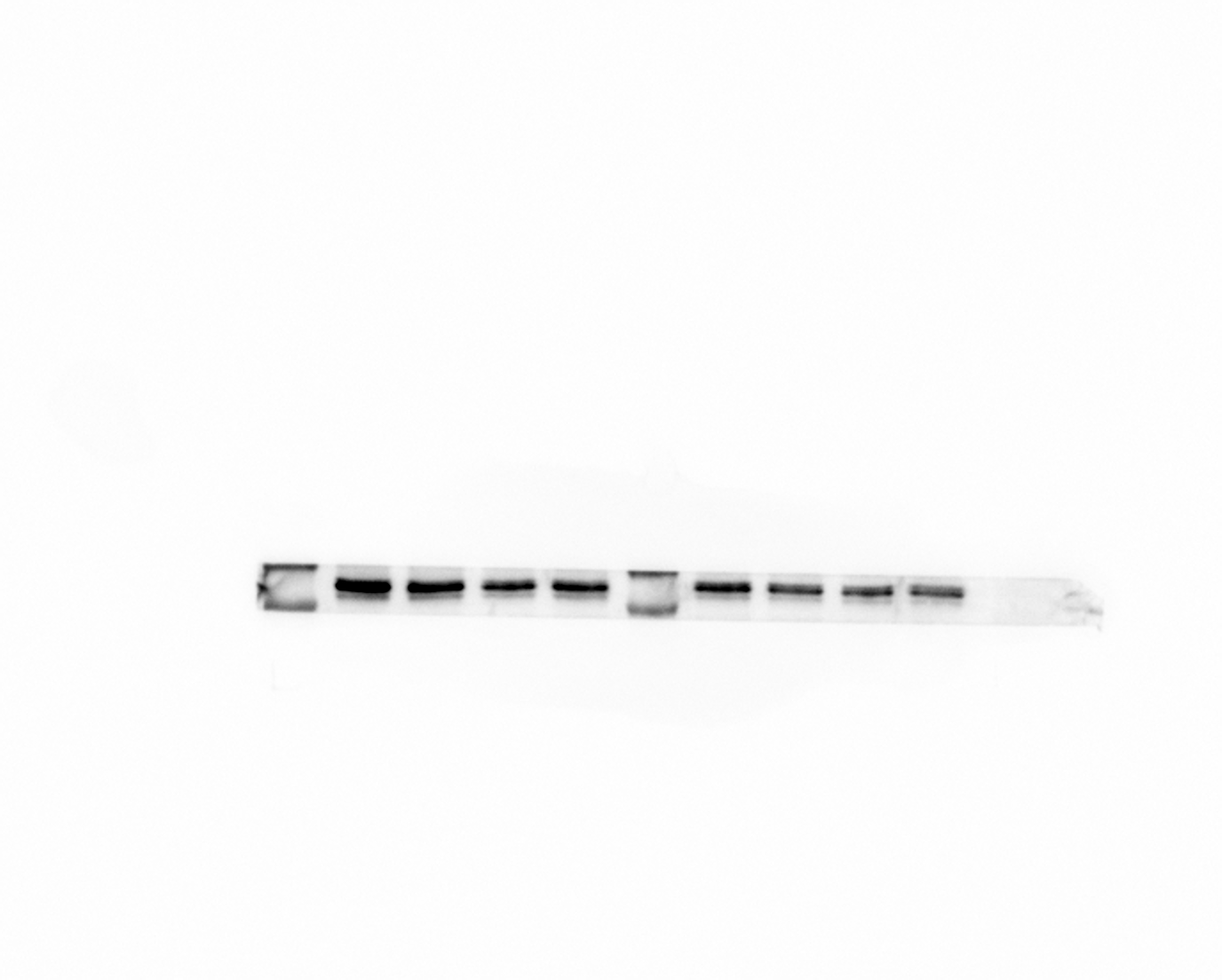

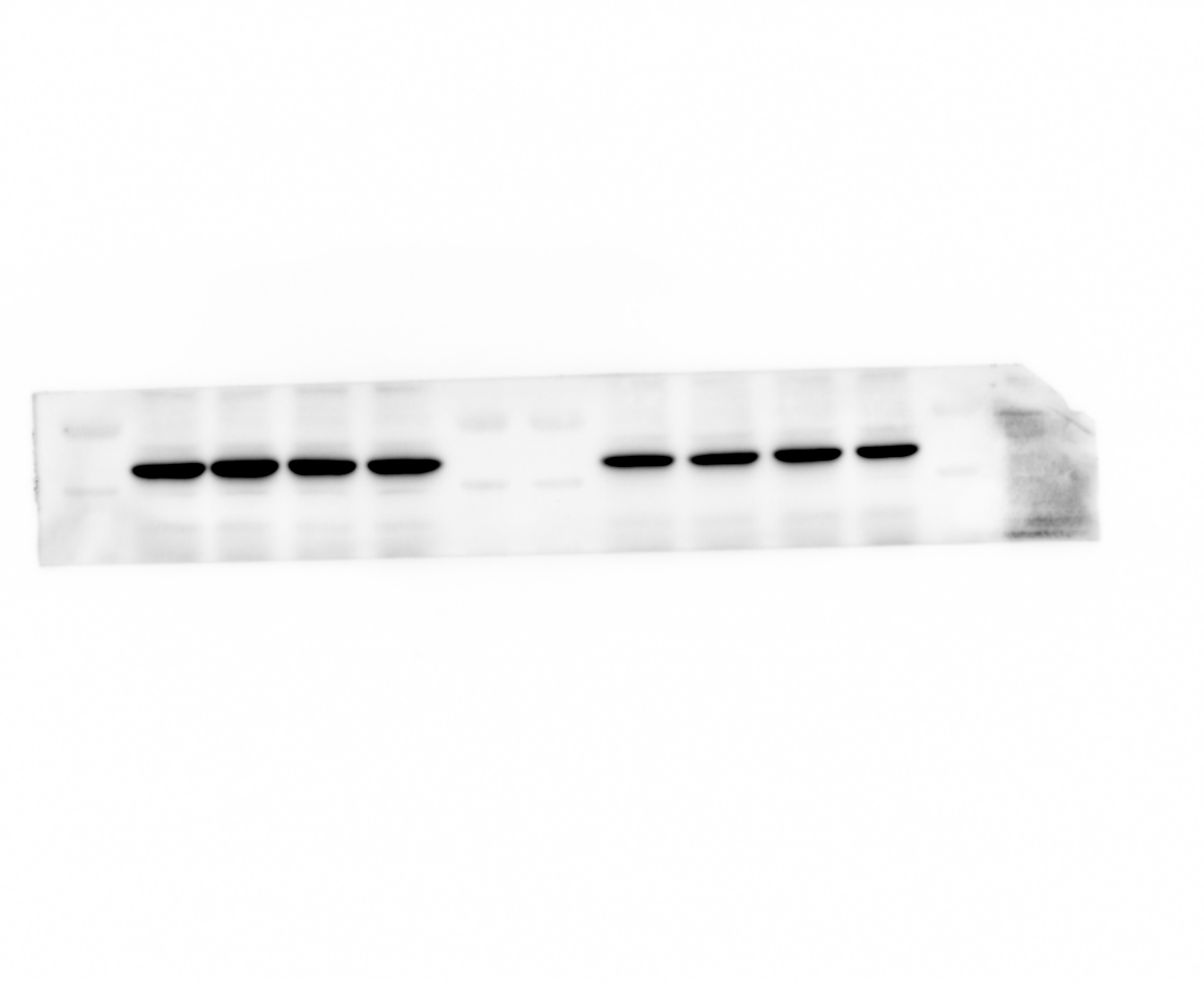


MFN2 β-actin


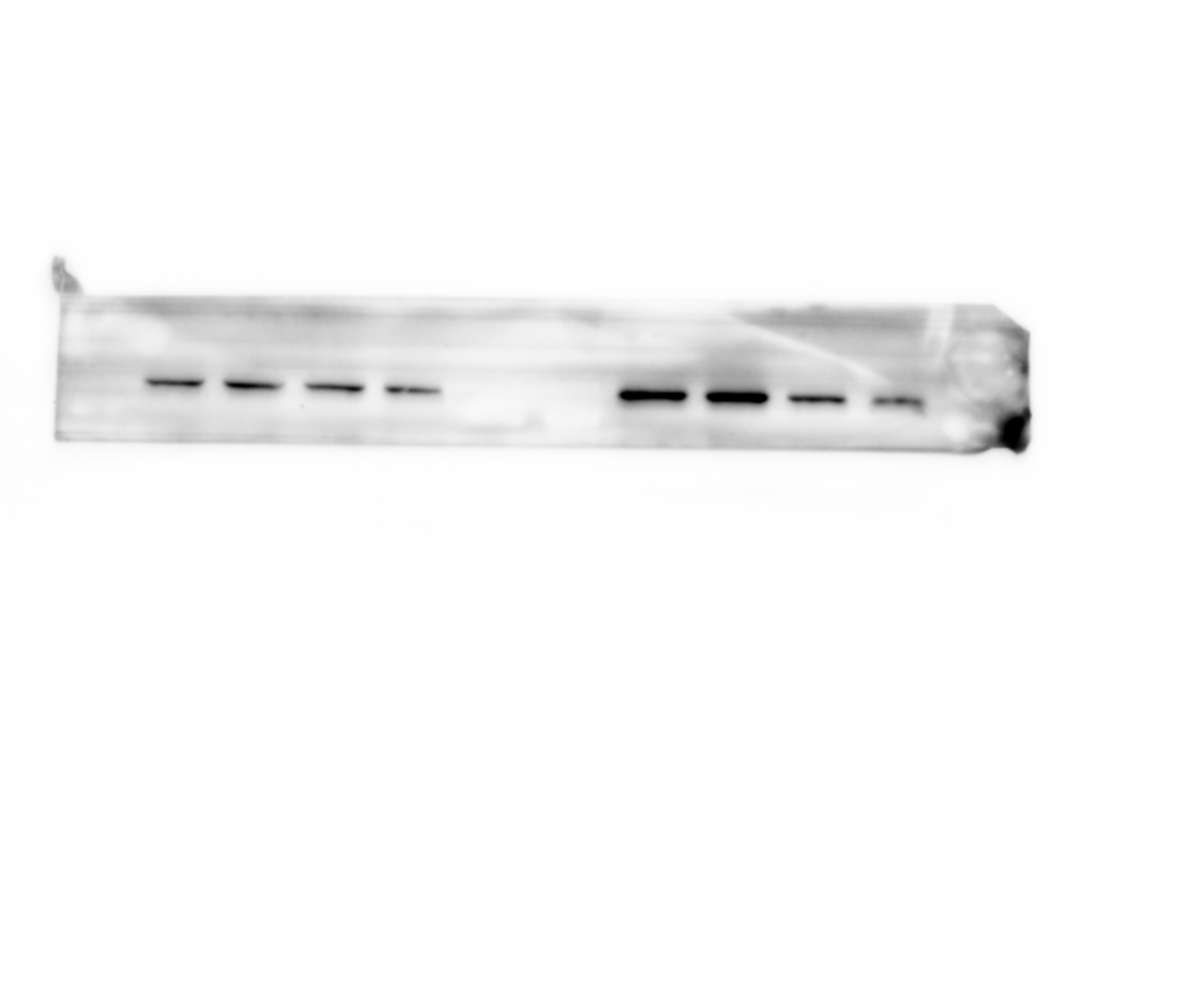


MFN2


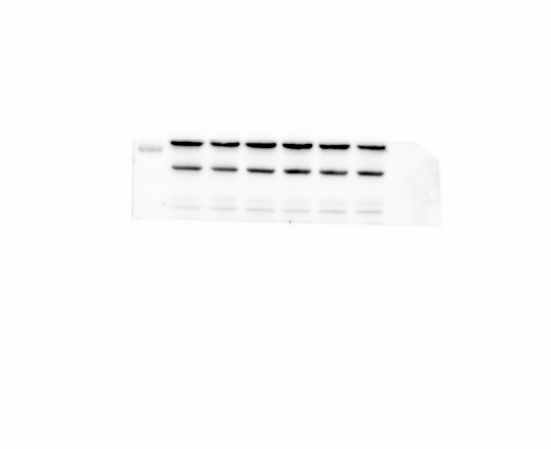

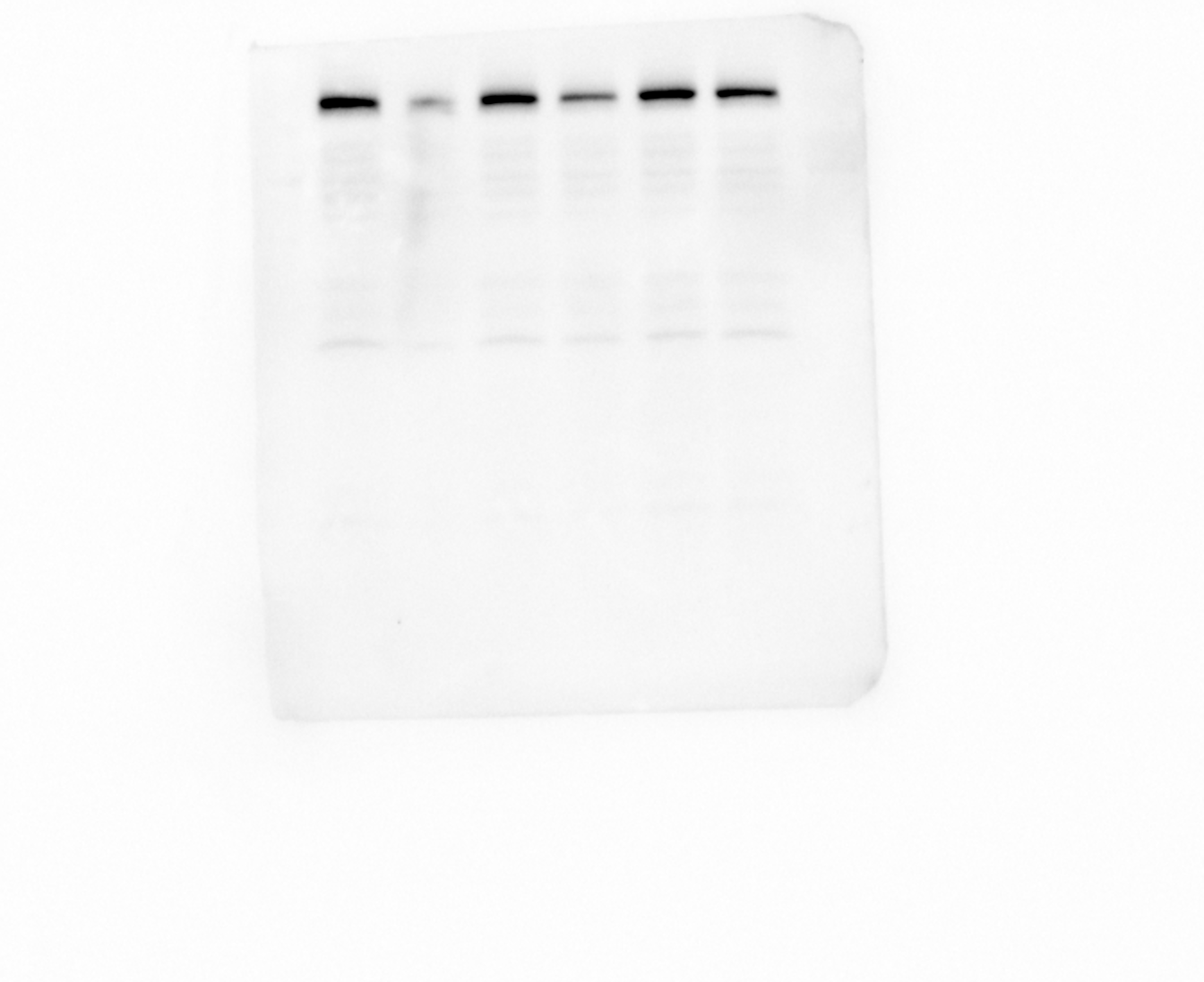


MFN2 β-actin


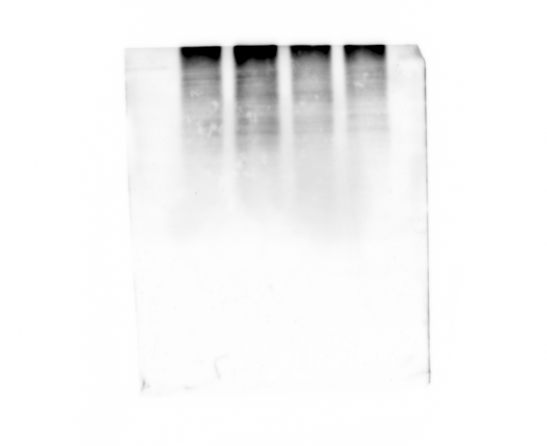

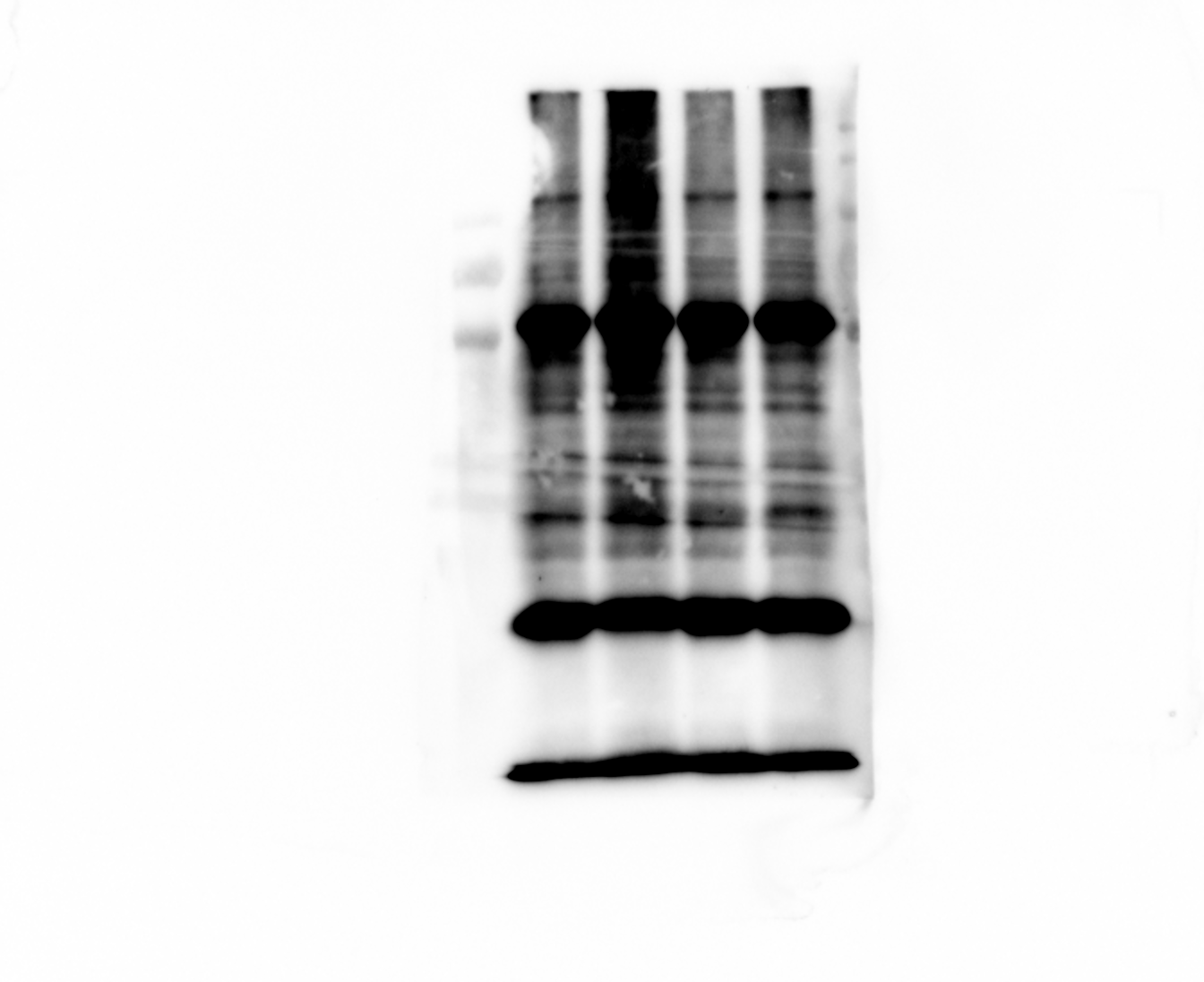


UB UB

**Fig S5**


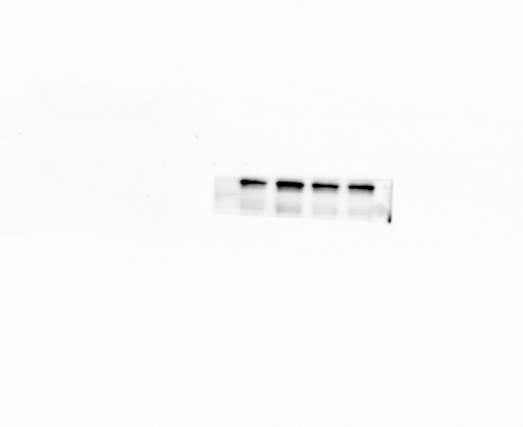

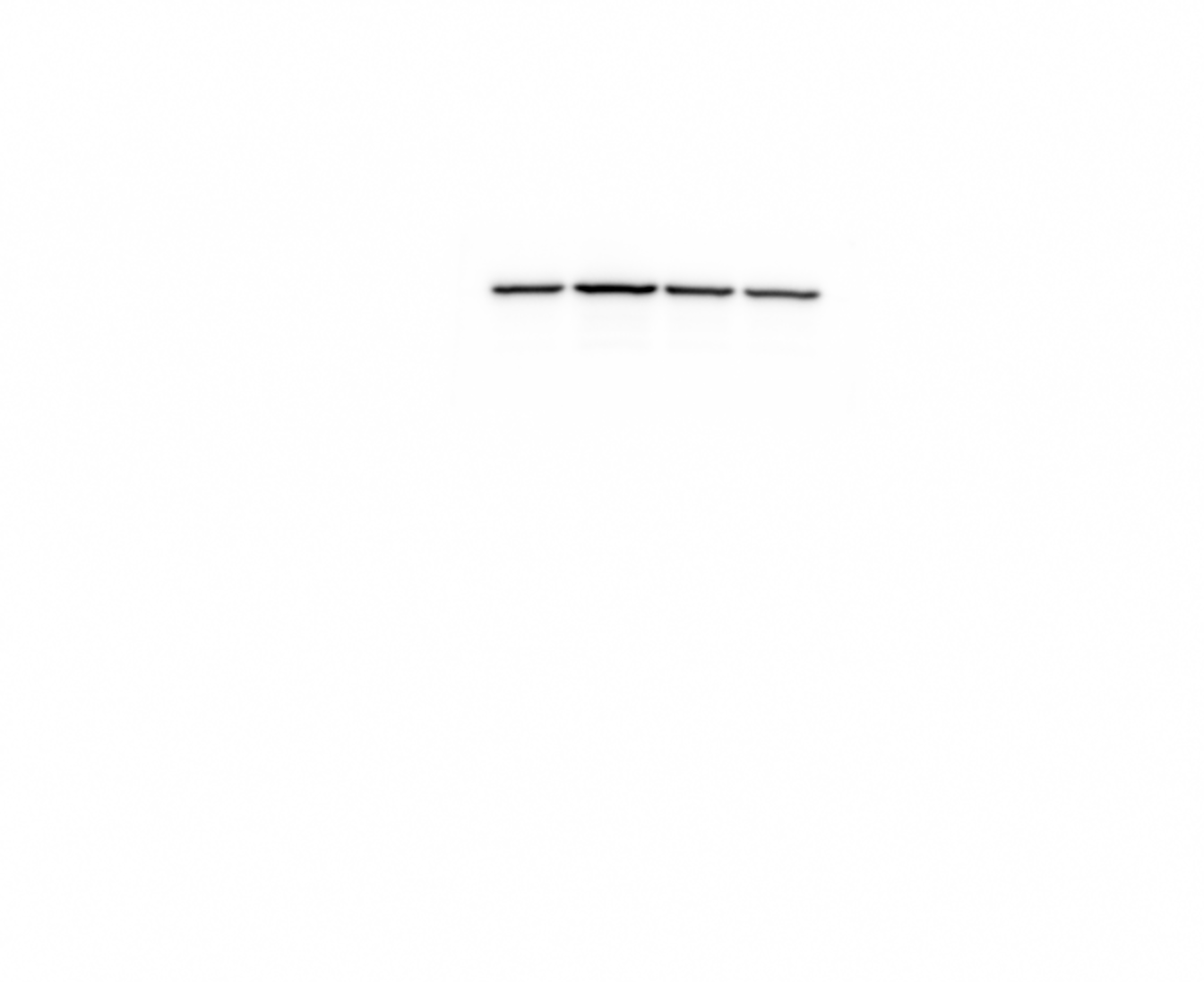


Cytc Cleaved-cas3


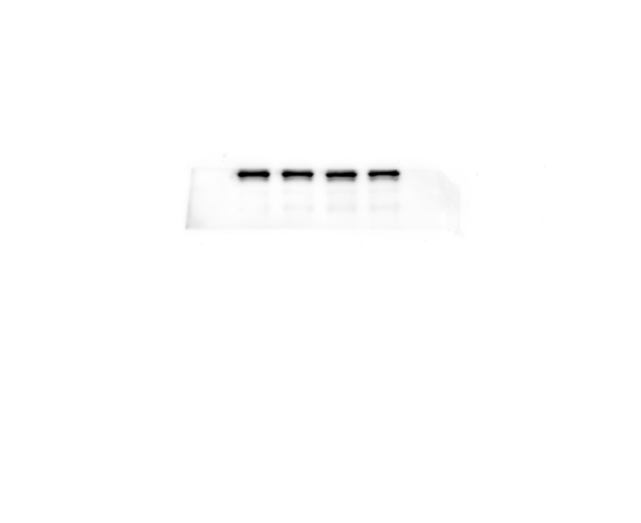

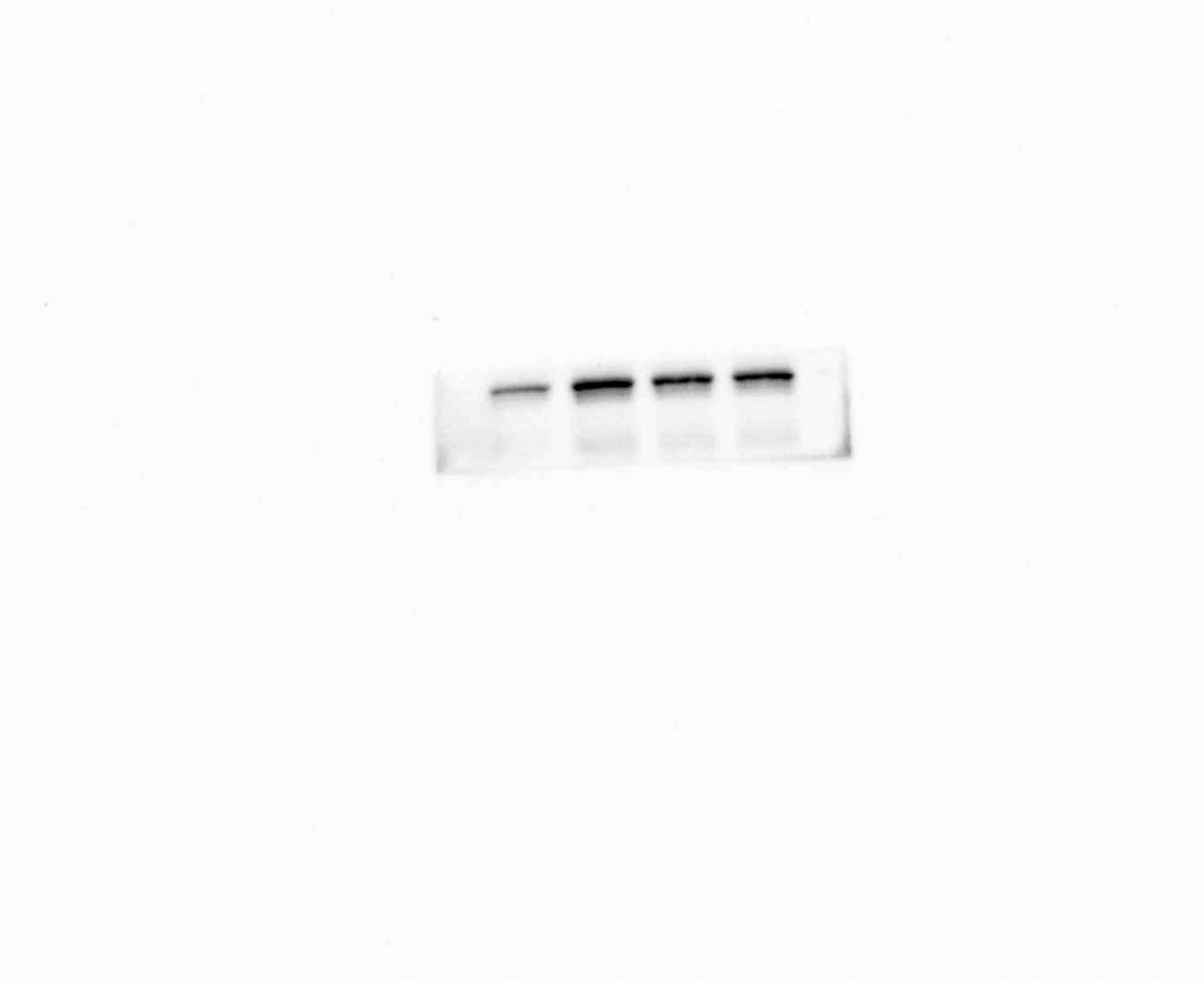


Cleaved-cas9 β-actin



**Fig 6**


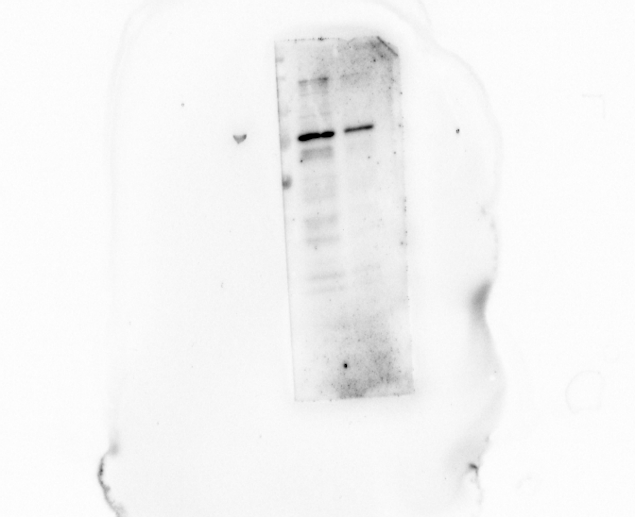


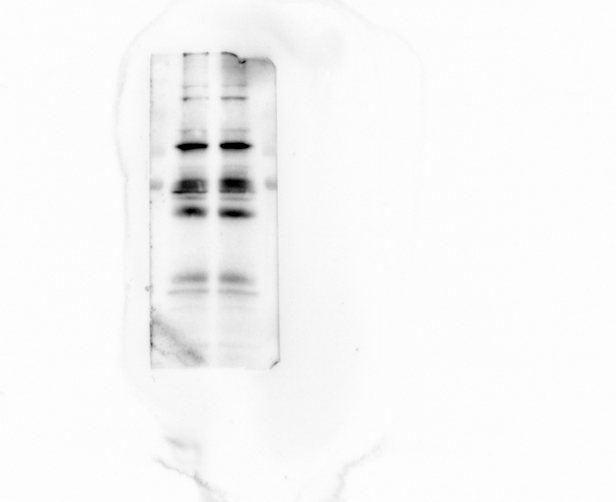

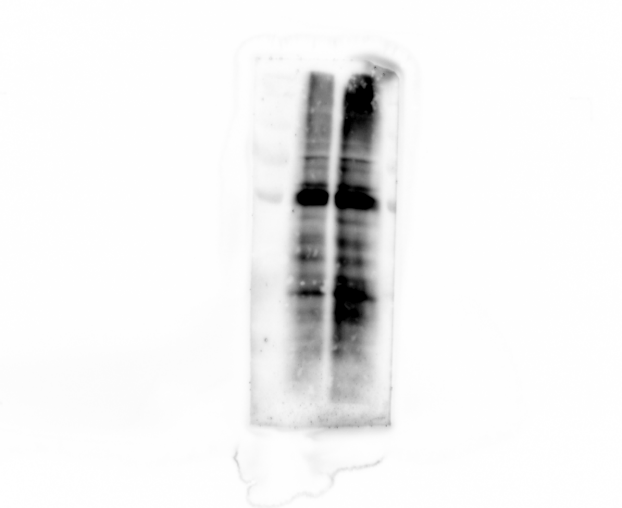
**SLC3A2 β-actin**

**UB SLC3A2**


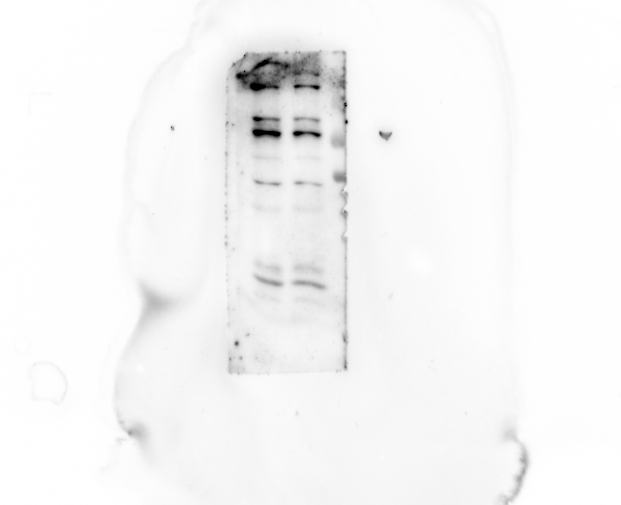

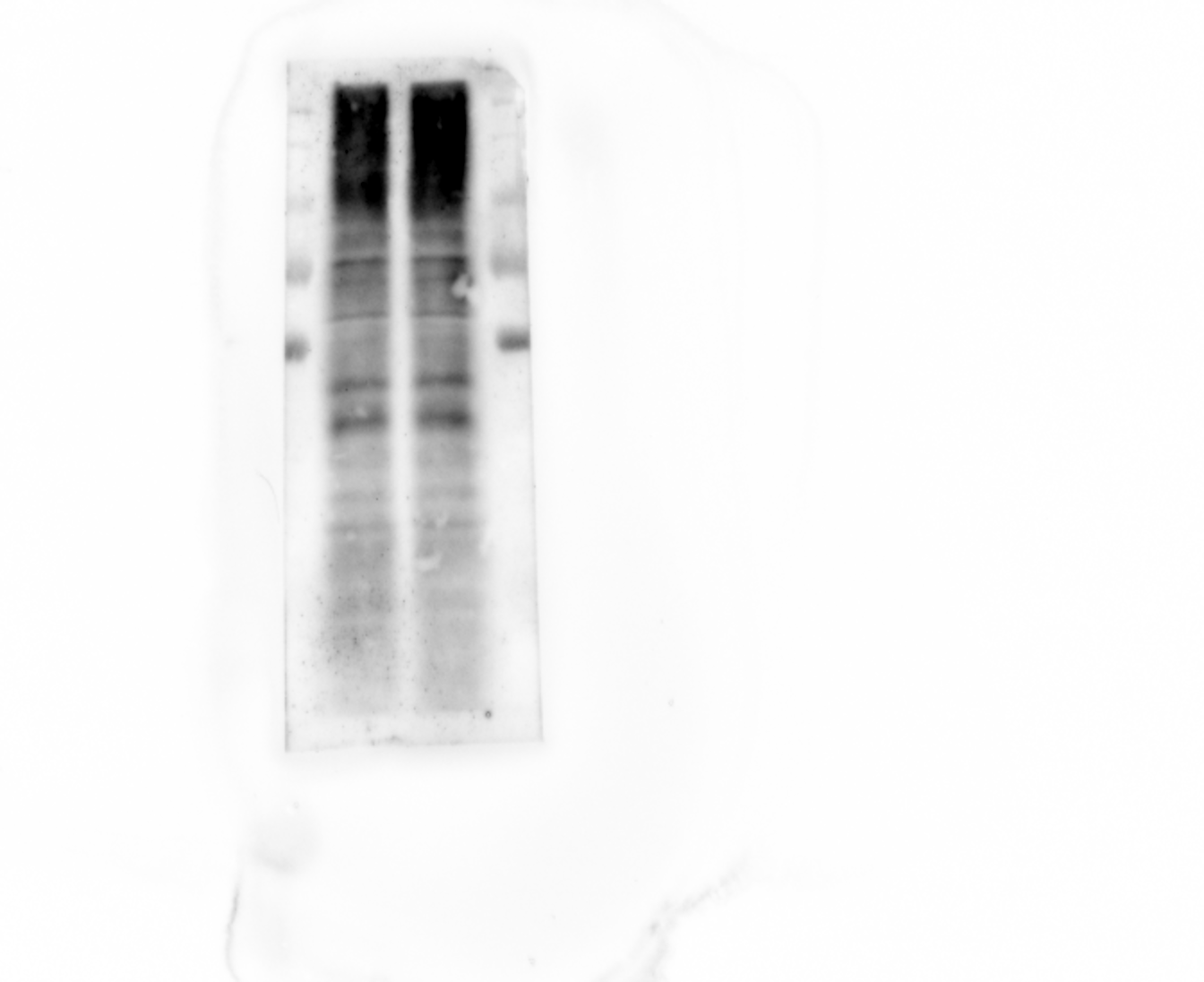


**UB SLC3A2**


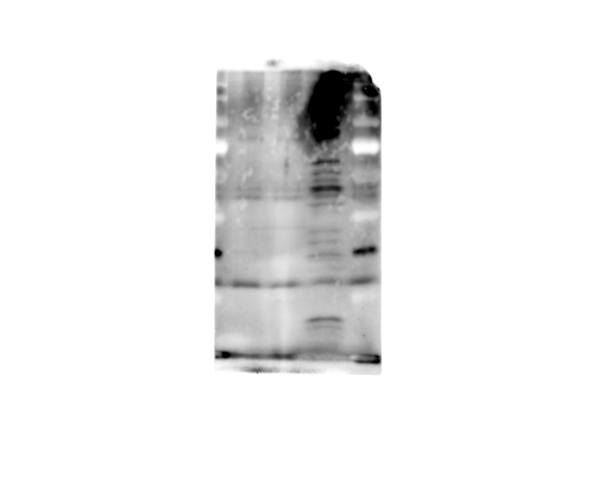

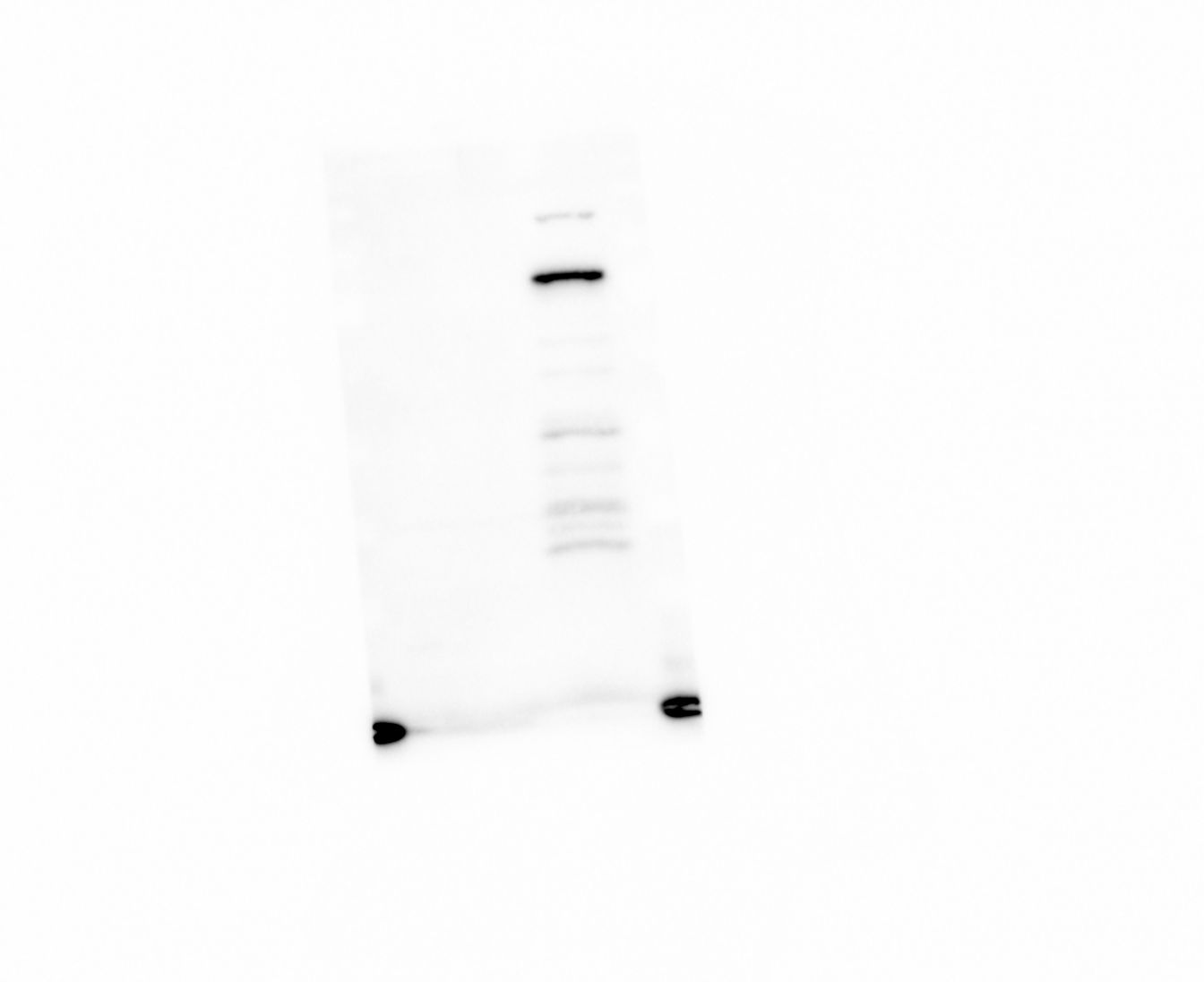


**SLC3A2 TRIM21**


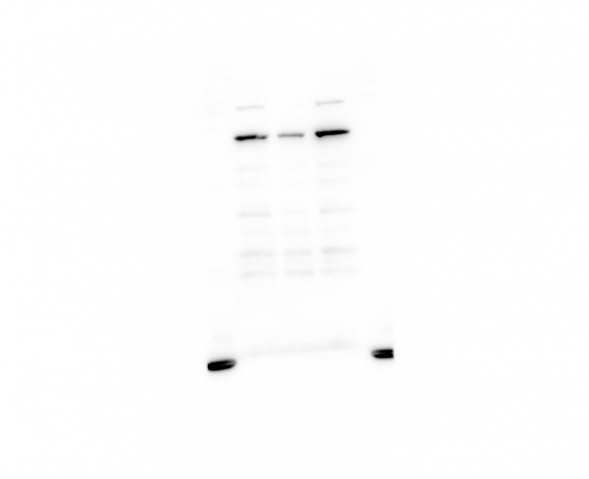

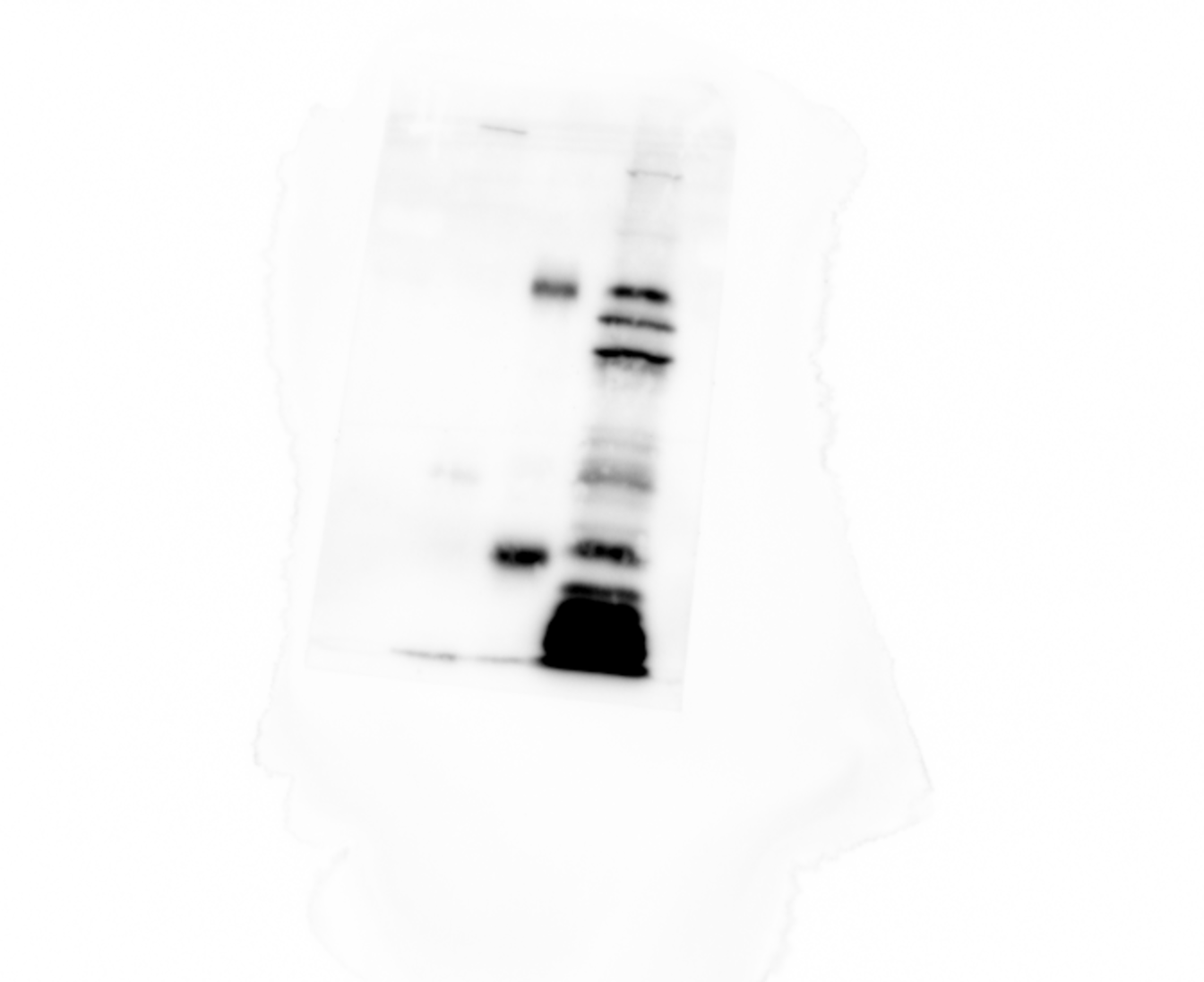


**PTDSS1 SLC3A2**


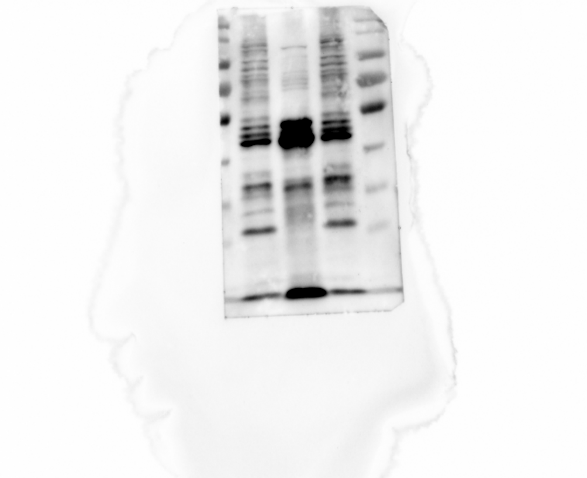

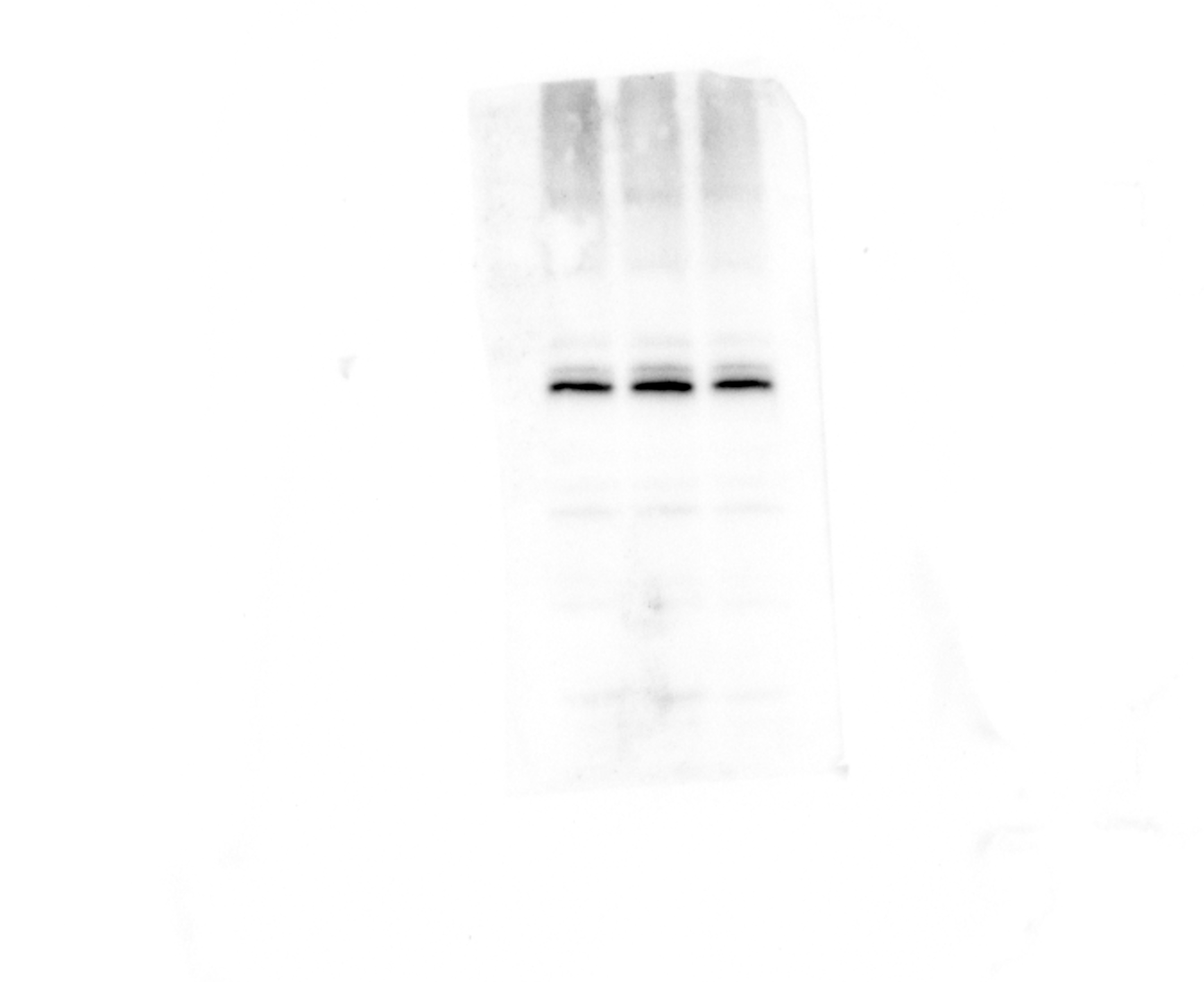


**TRIM21 PTDSS1**


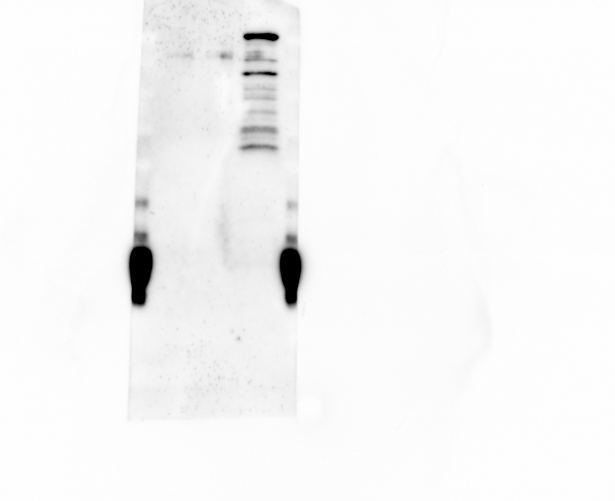

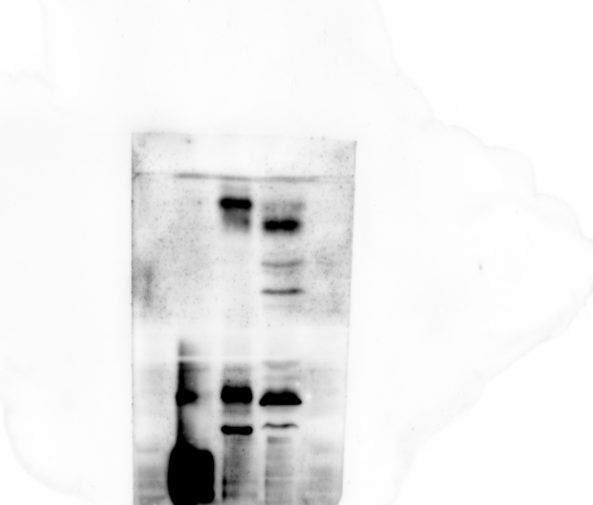


**TRIM21 SLC3A2**


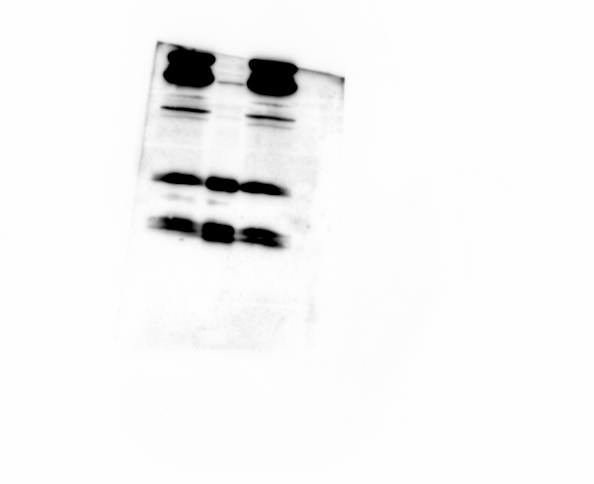

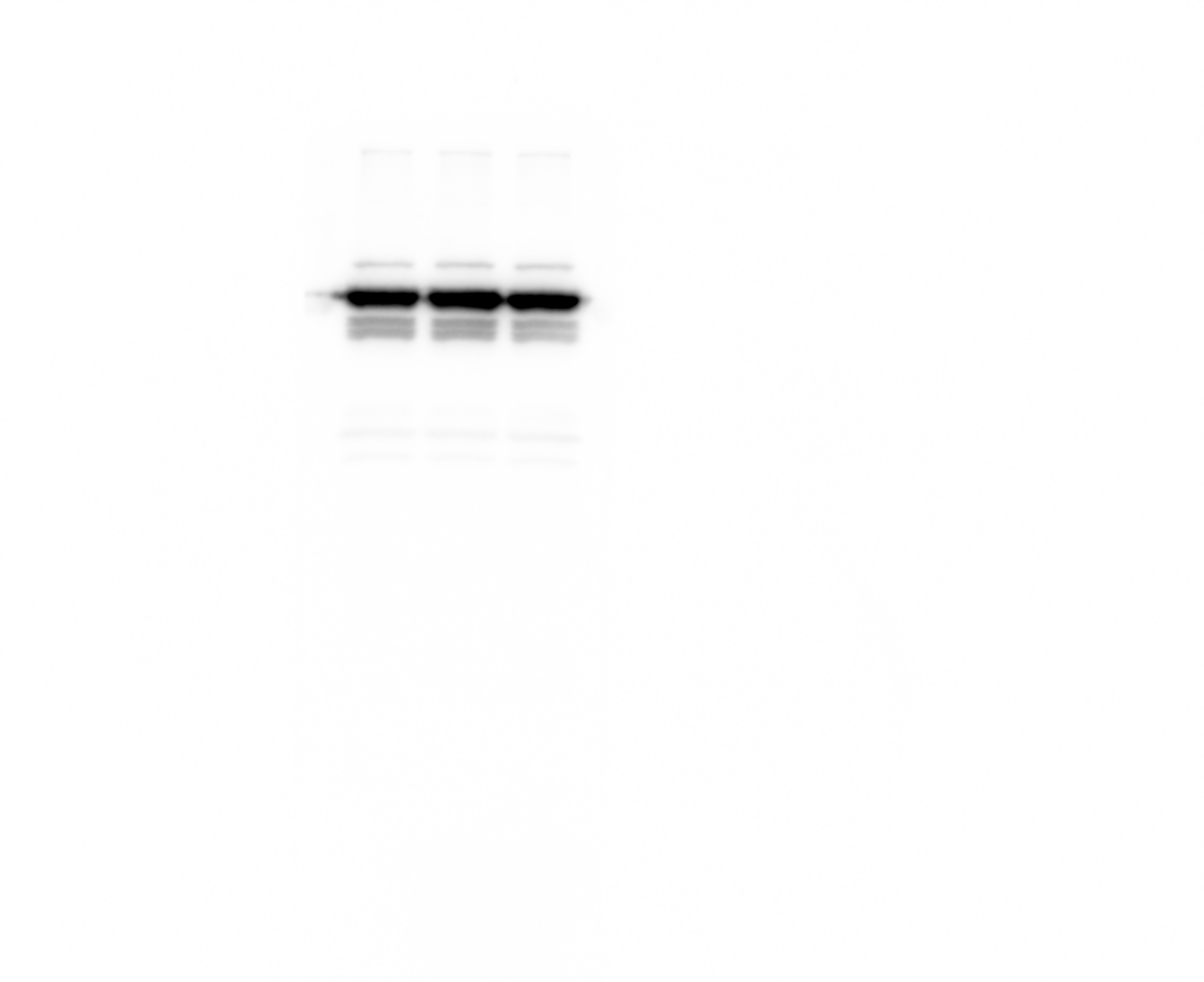


**TRIM21 SLC3A2**


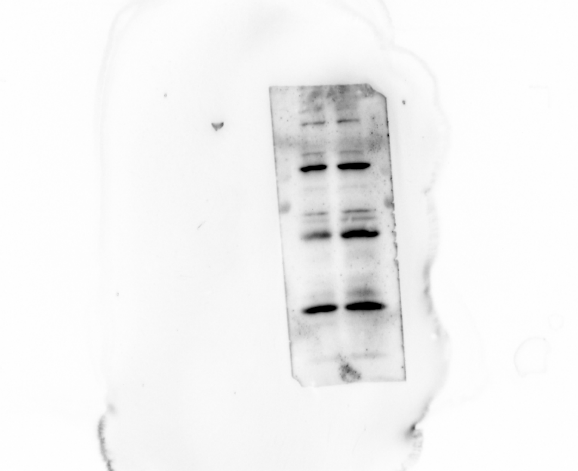

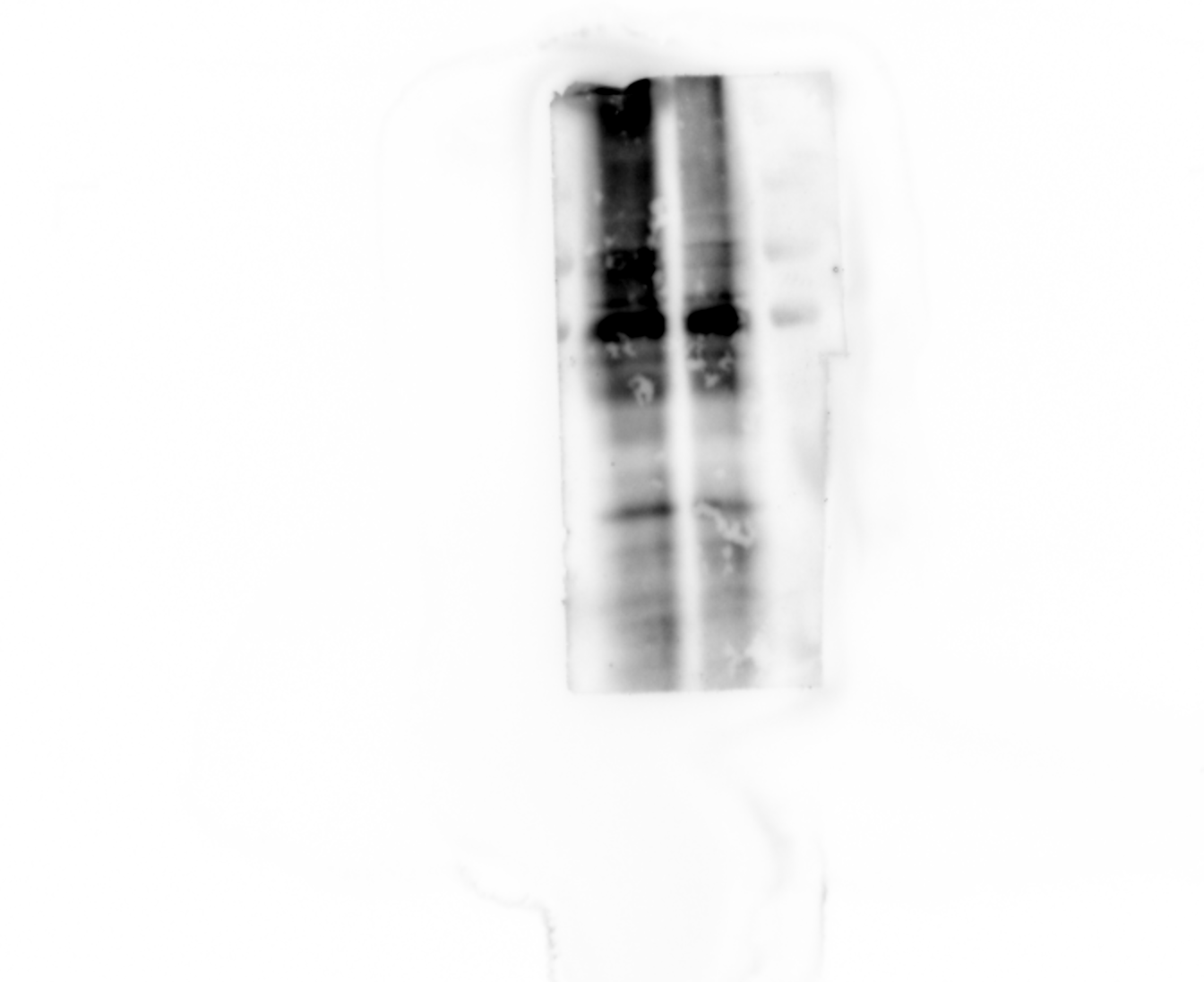


**UB SLC3A2**


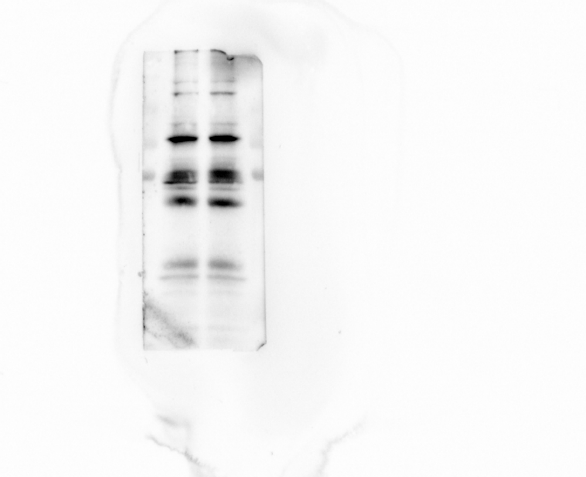

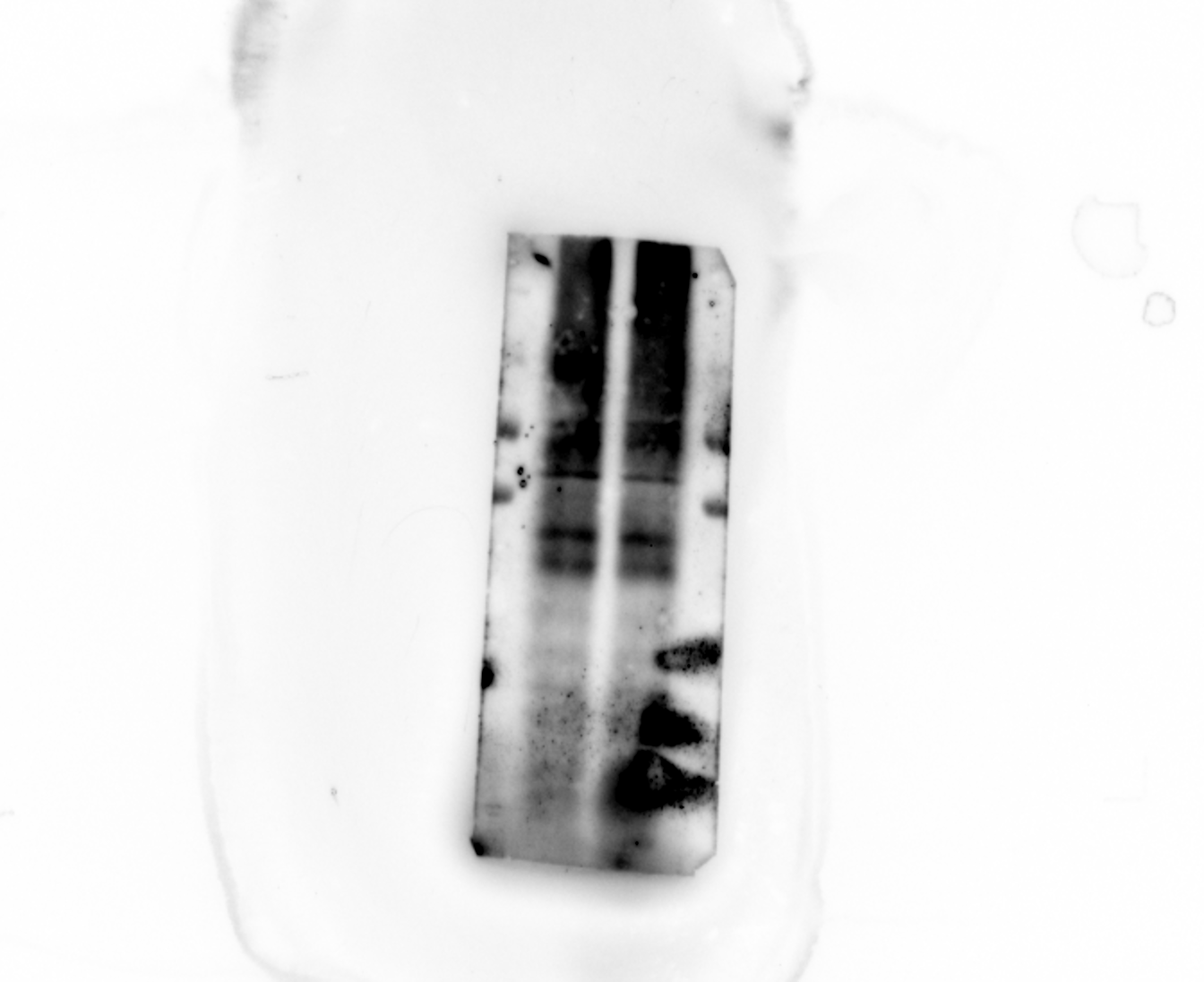


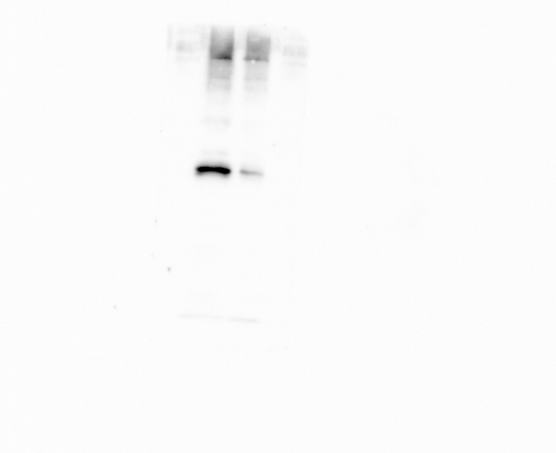
**UB SLC3A2**


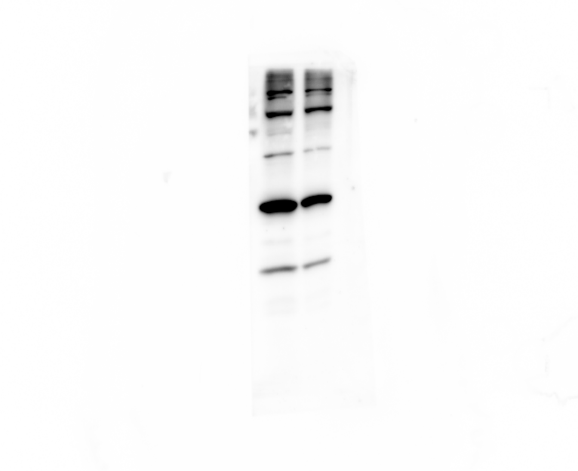


**TRIM21 PTDSS1**


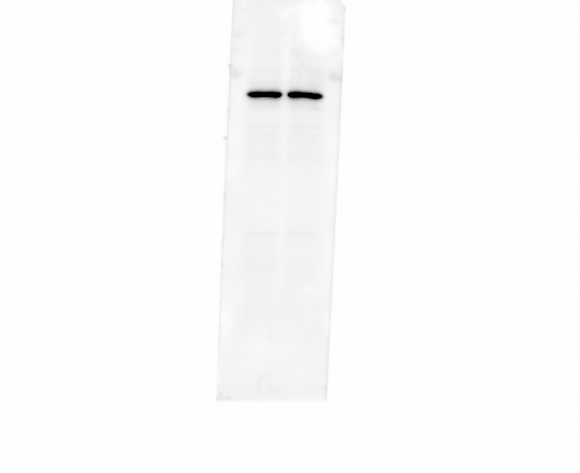

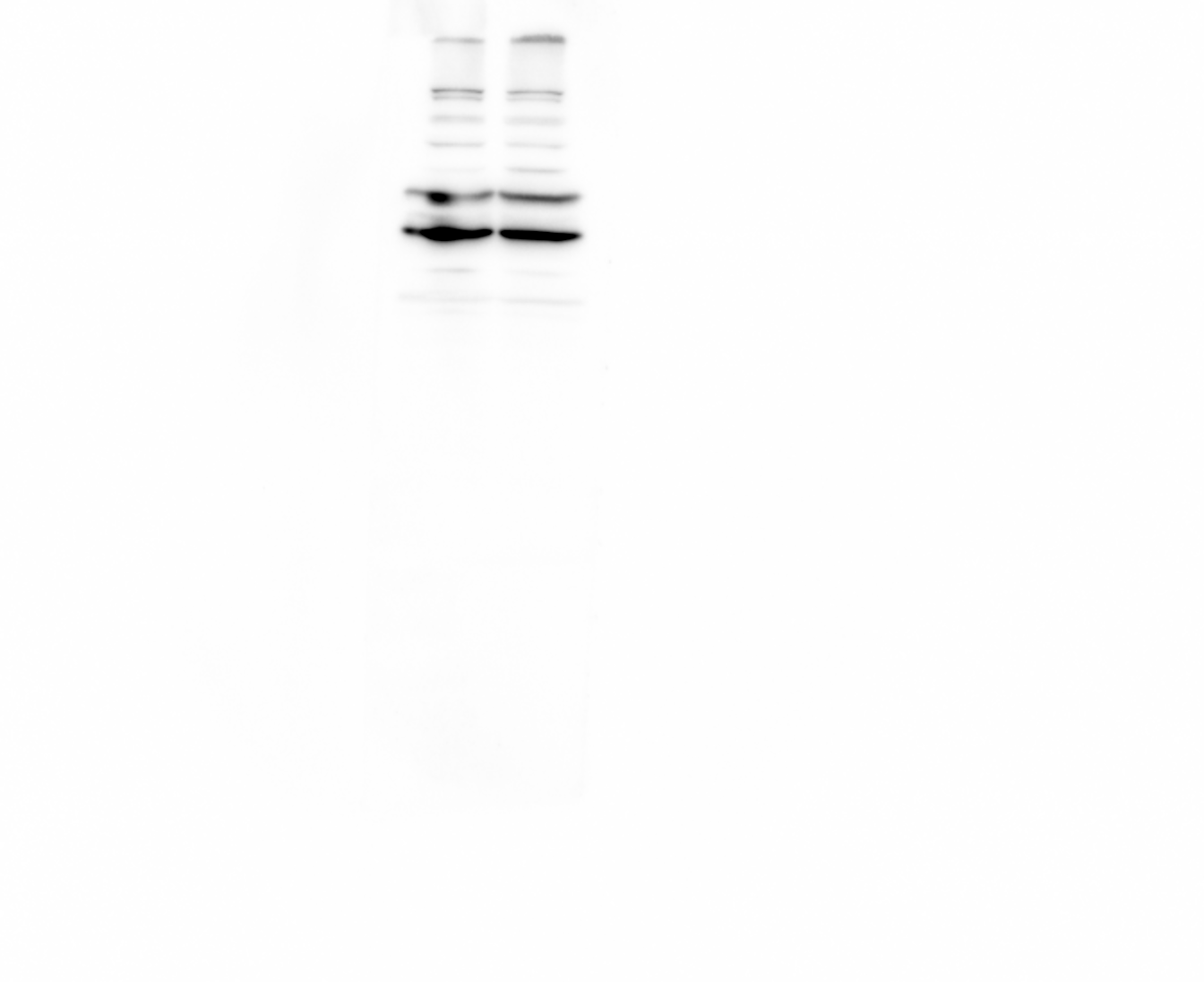


**TRIM21 β-actin**


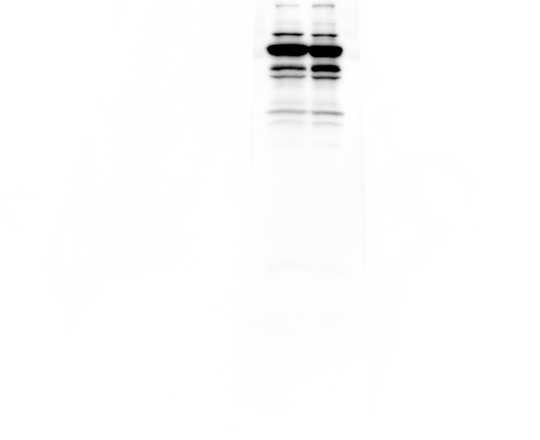

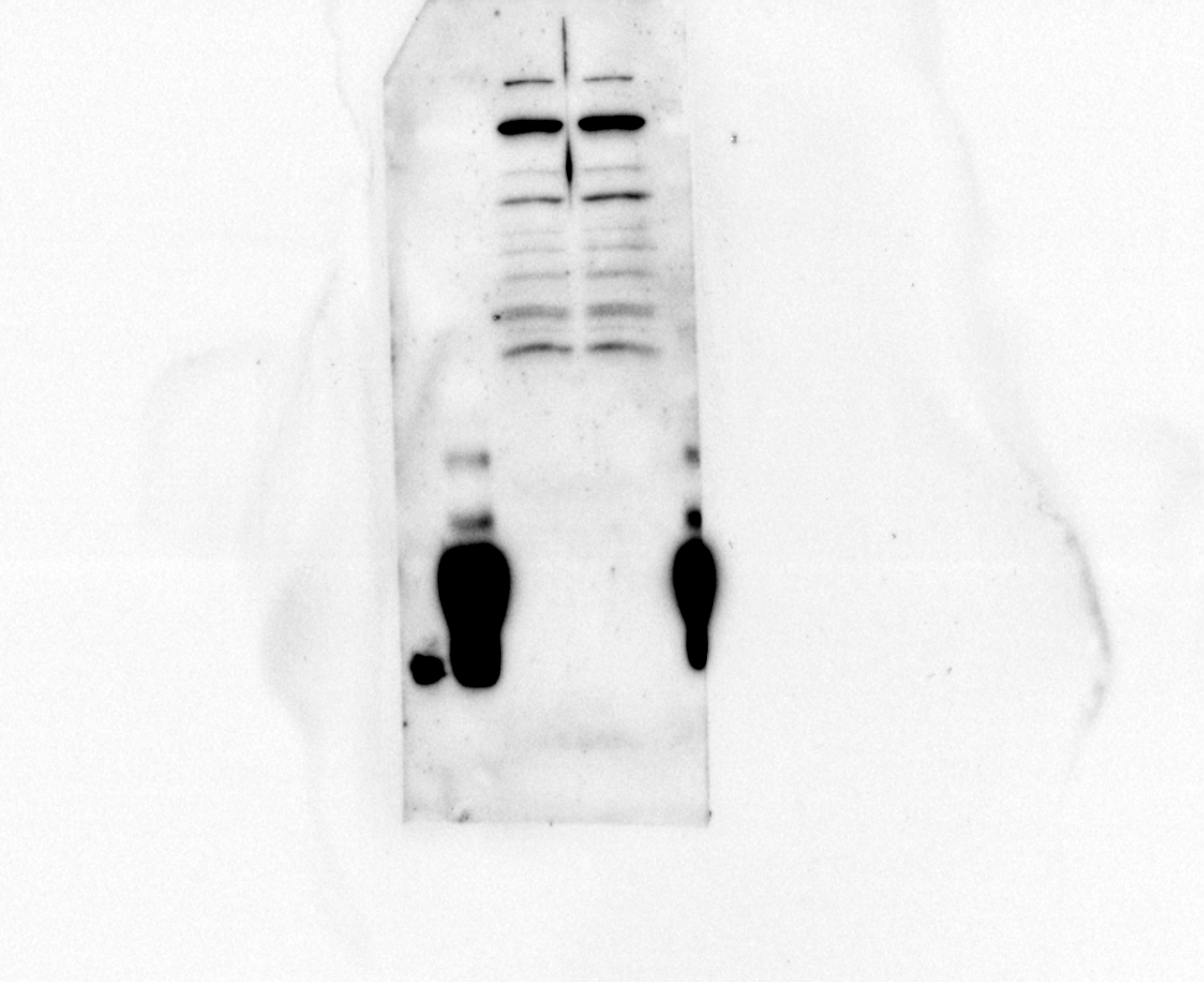


**TRIM21**  **Flag-SLC3A2**


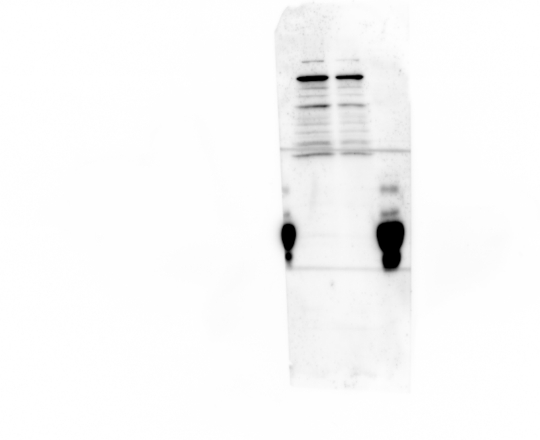

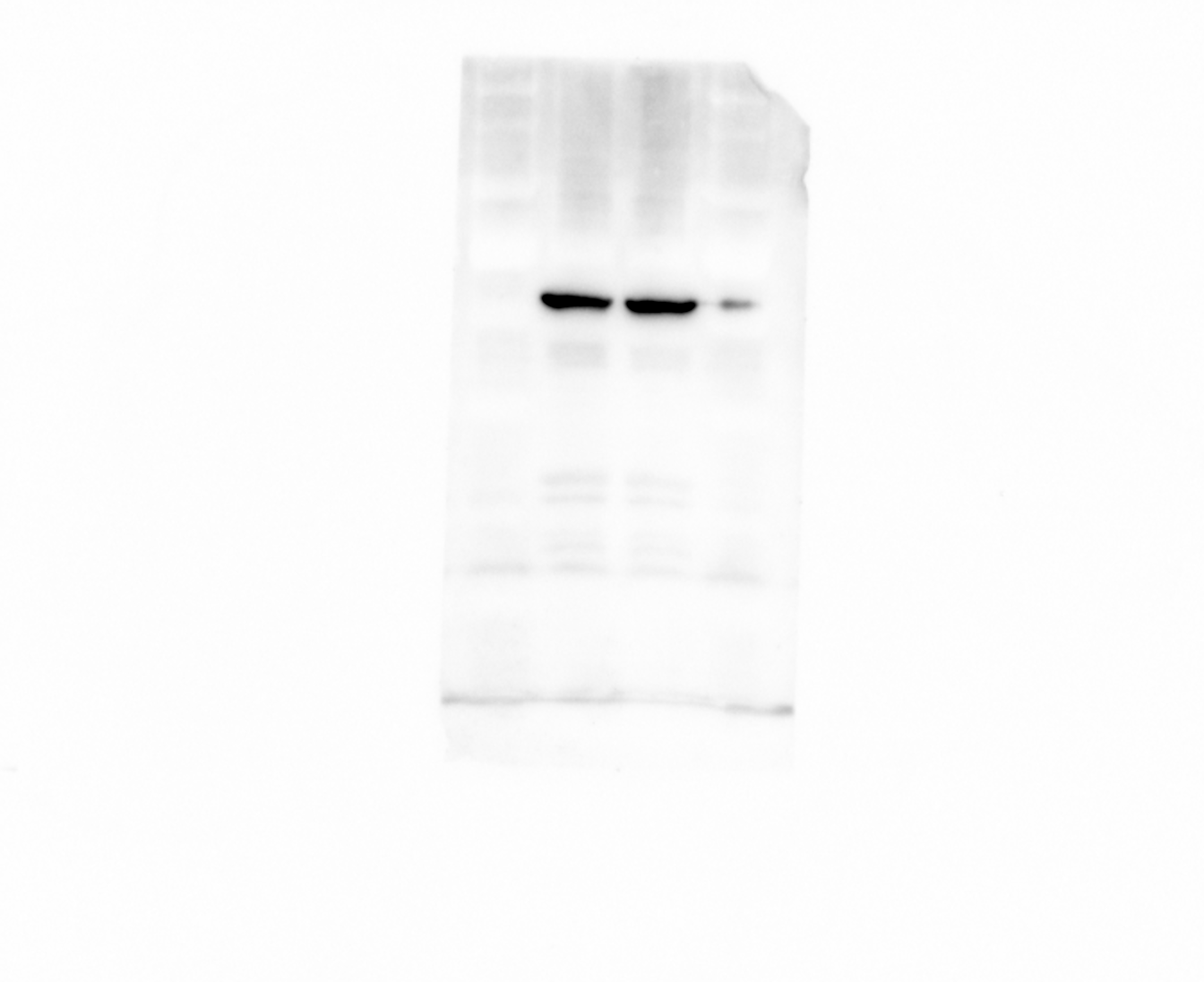


**TRIM21 Flag-SLC3A2**


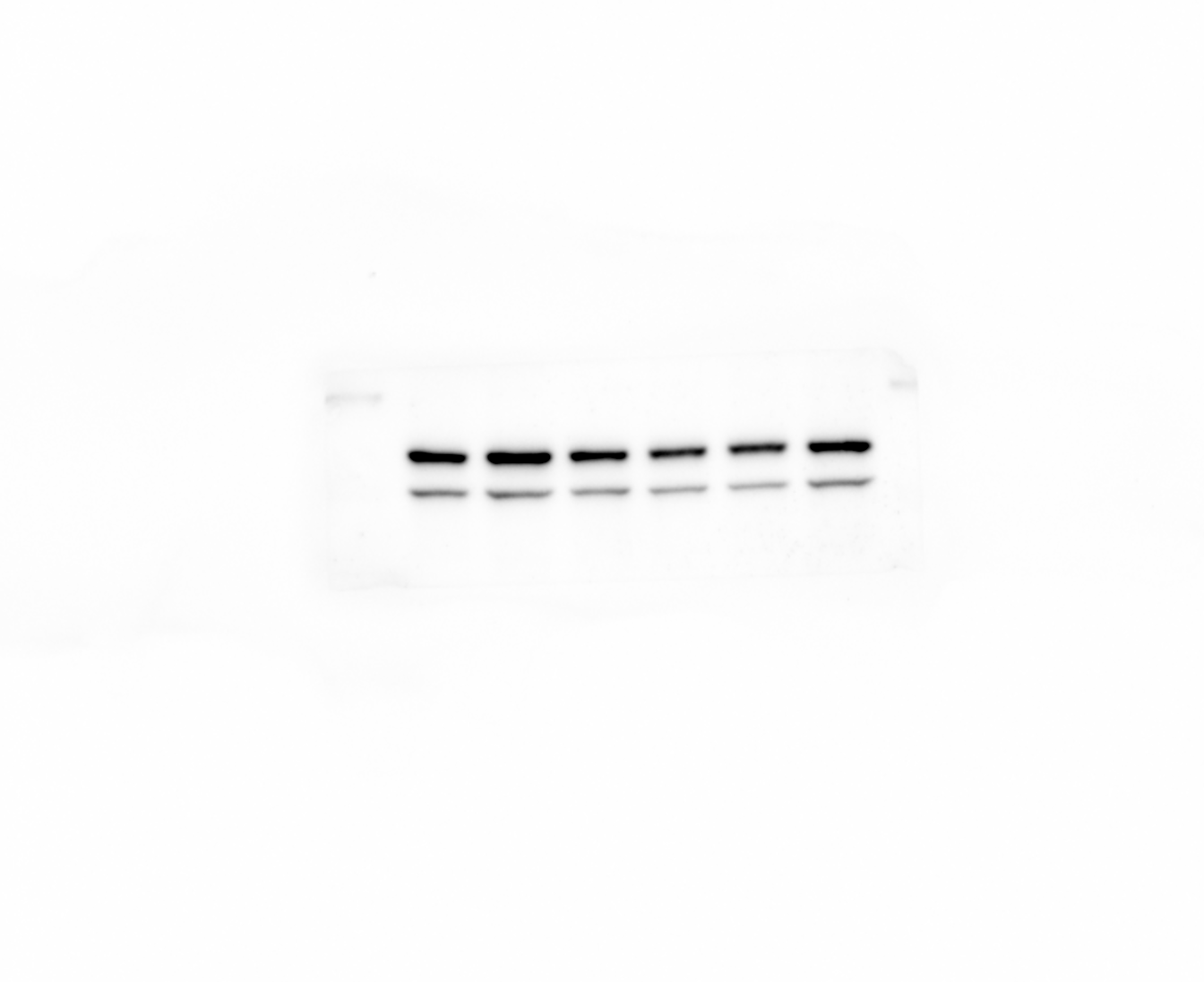

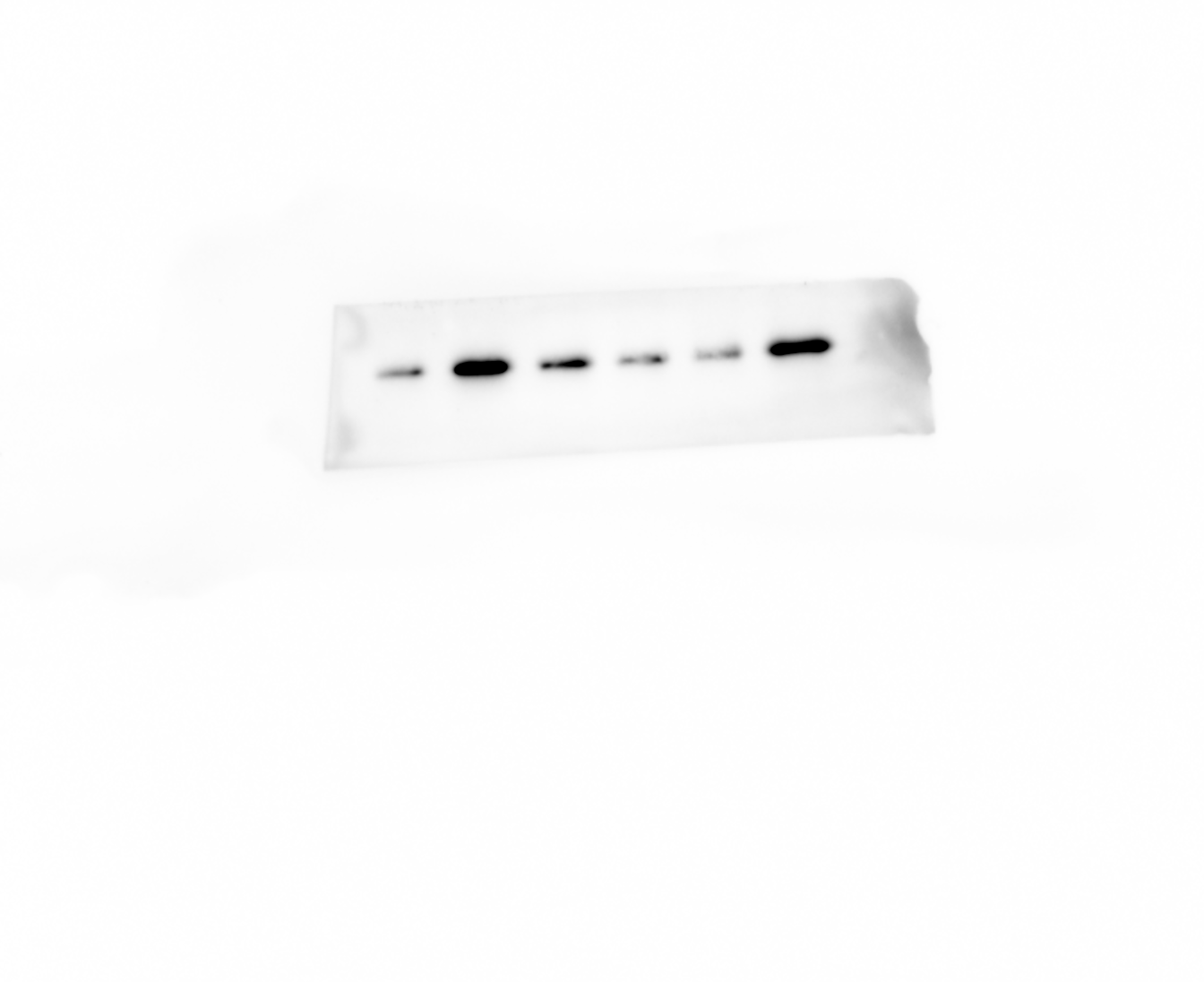


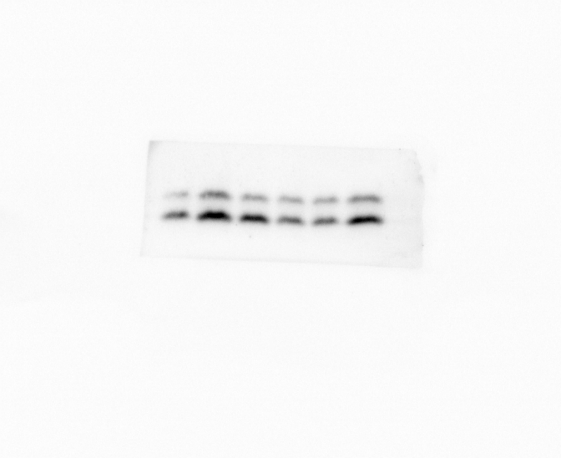
PINK1 Parkin


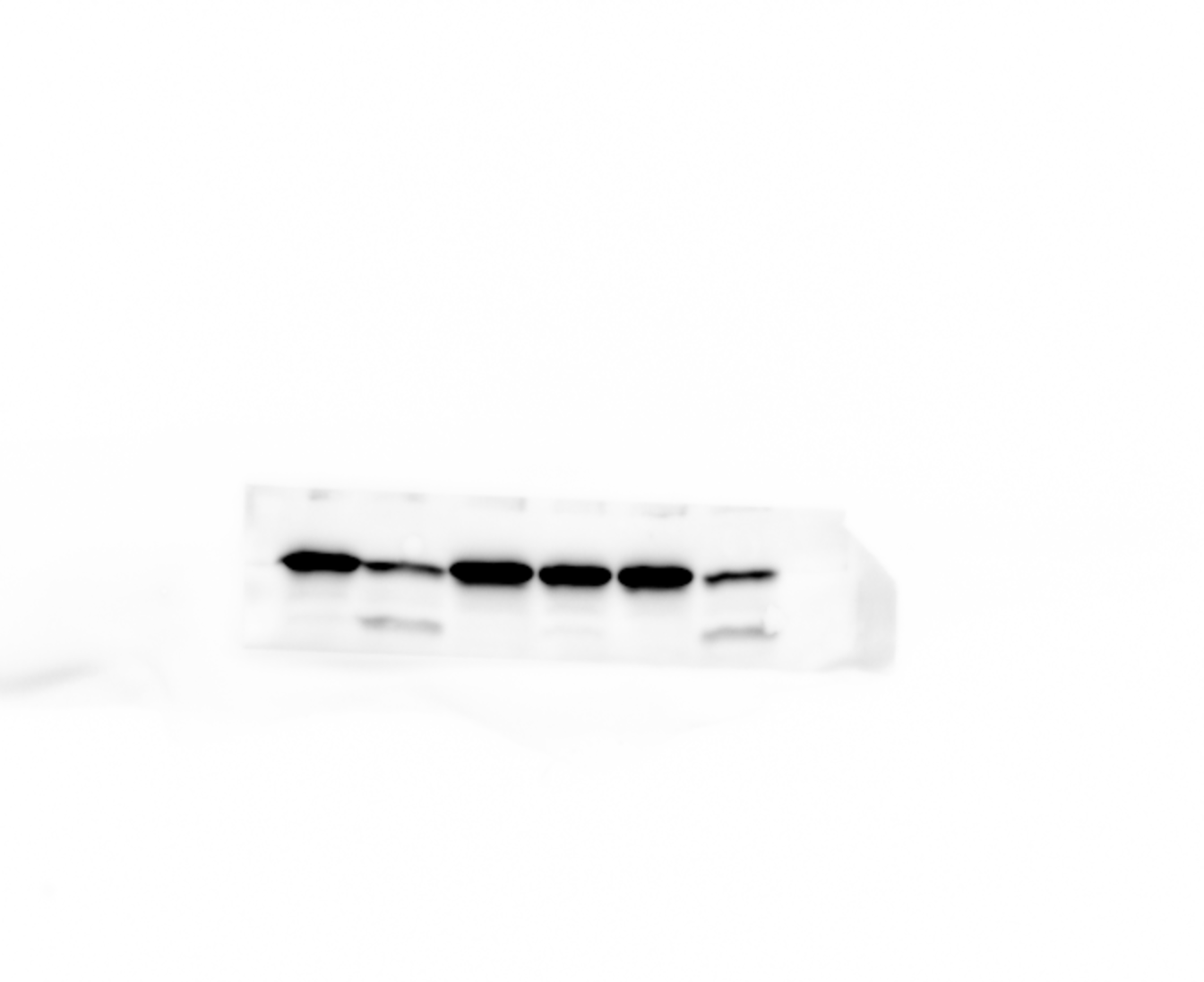


**P62 LCEI/II**


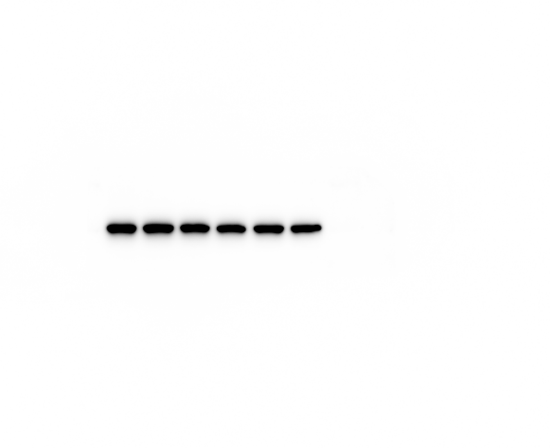

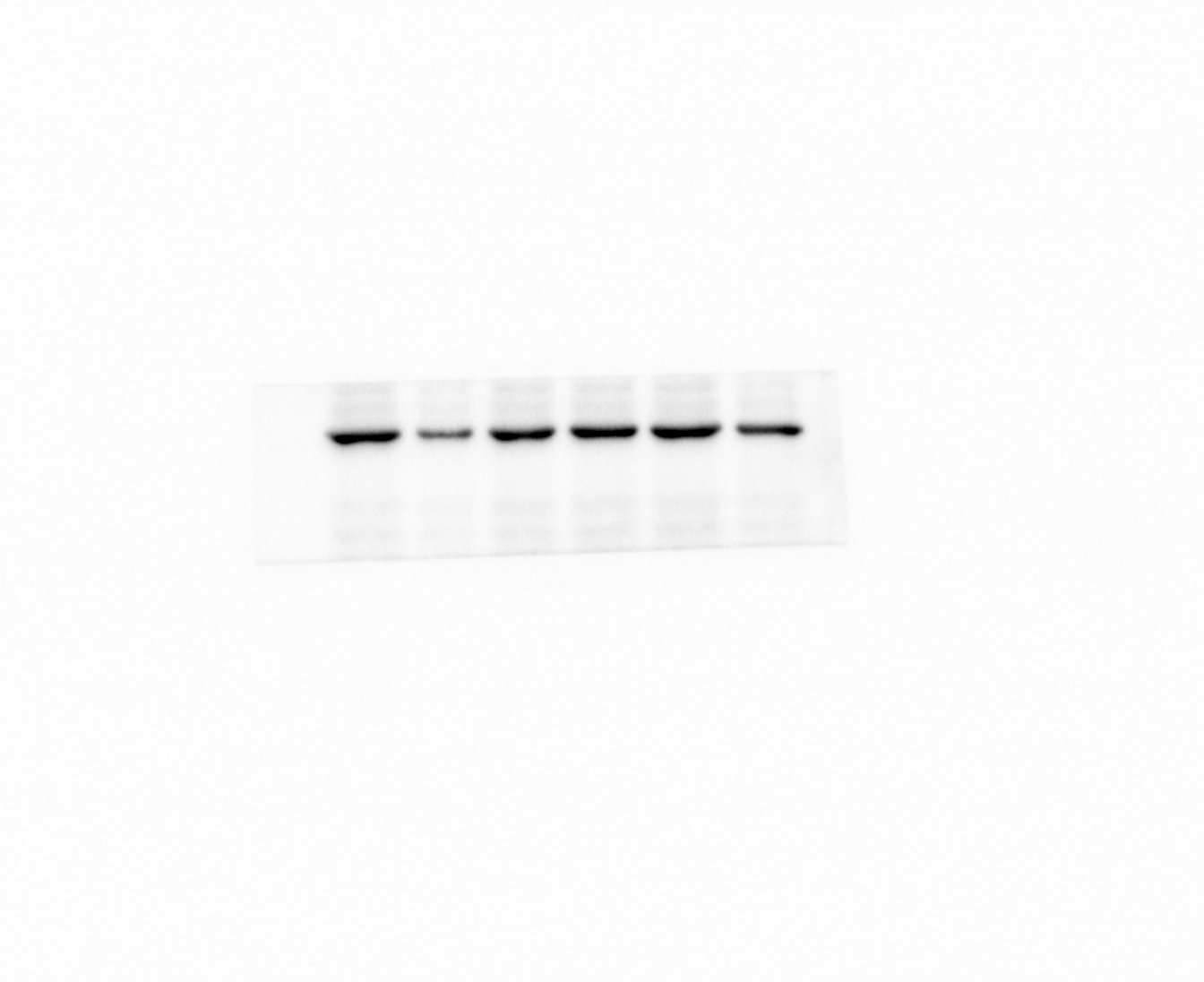


**MFN2 β-actin**

**Fig S6**


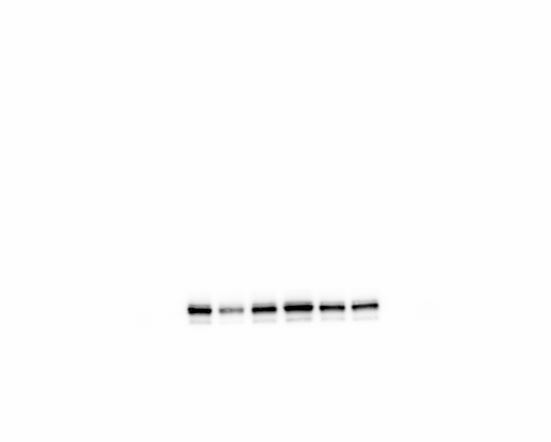

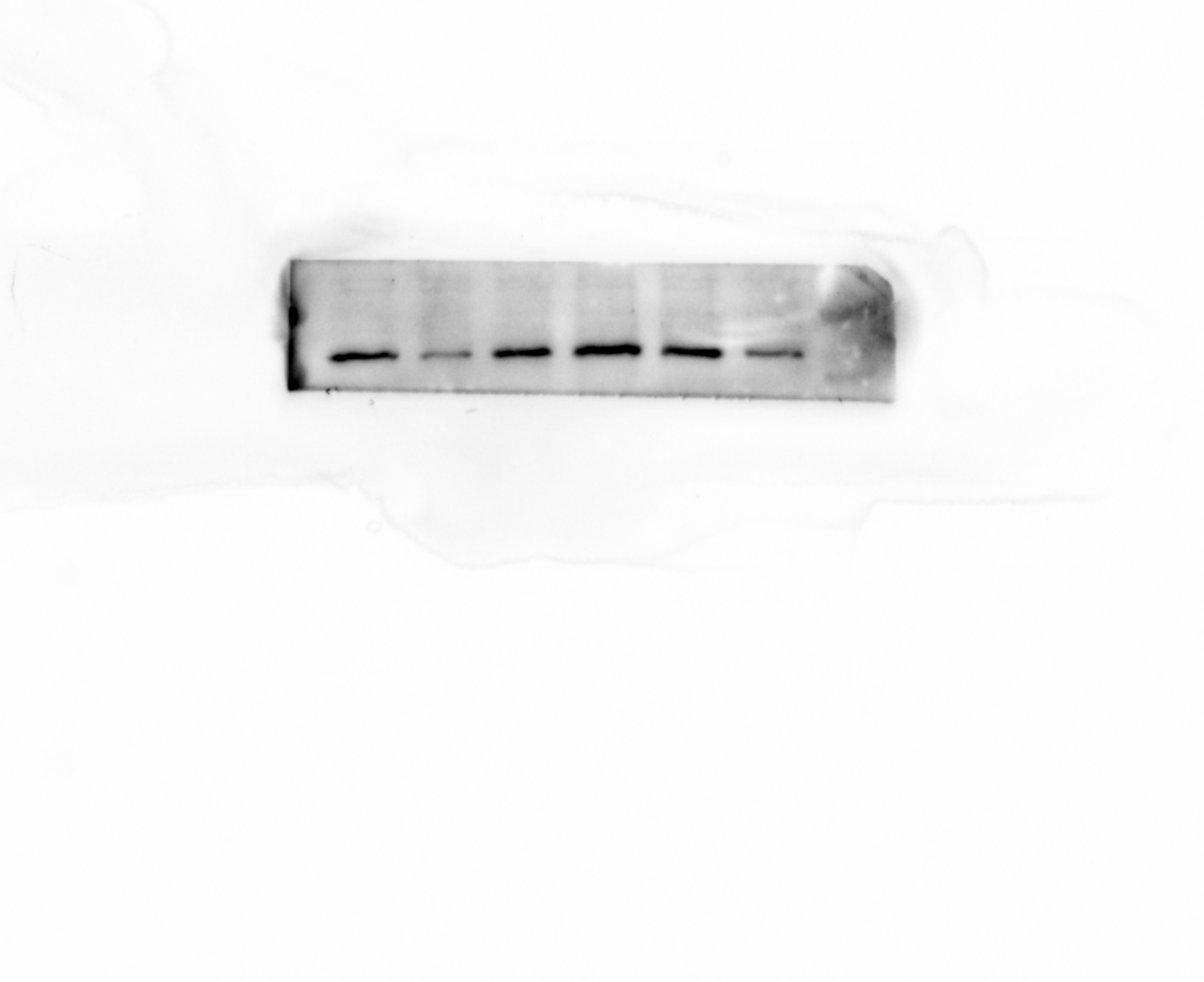


**SLC3A2 GCLC**


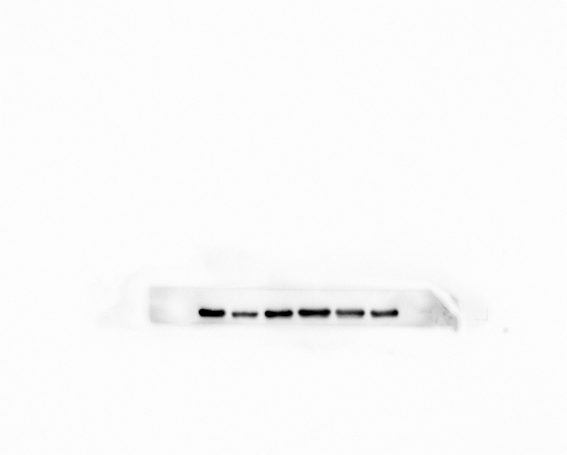

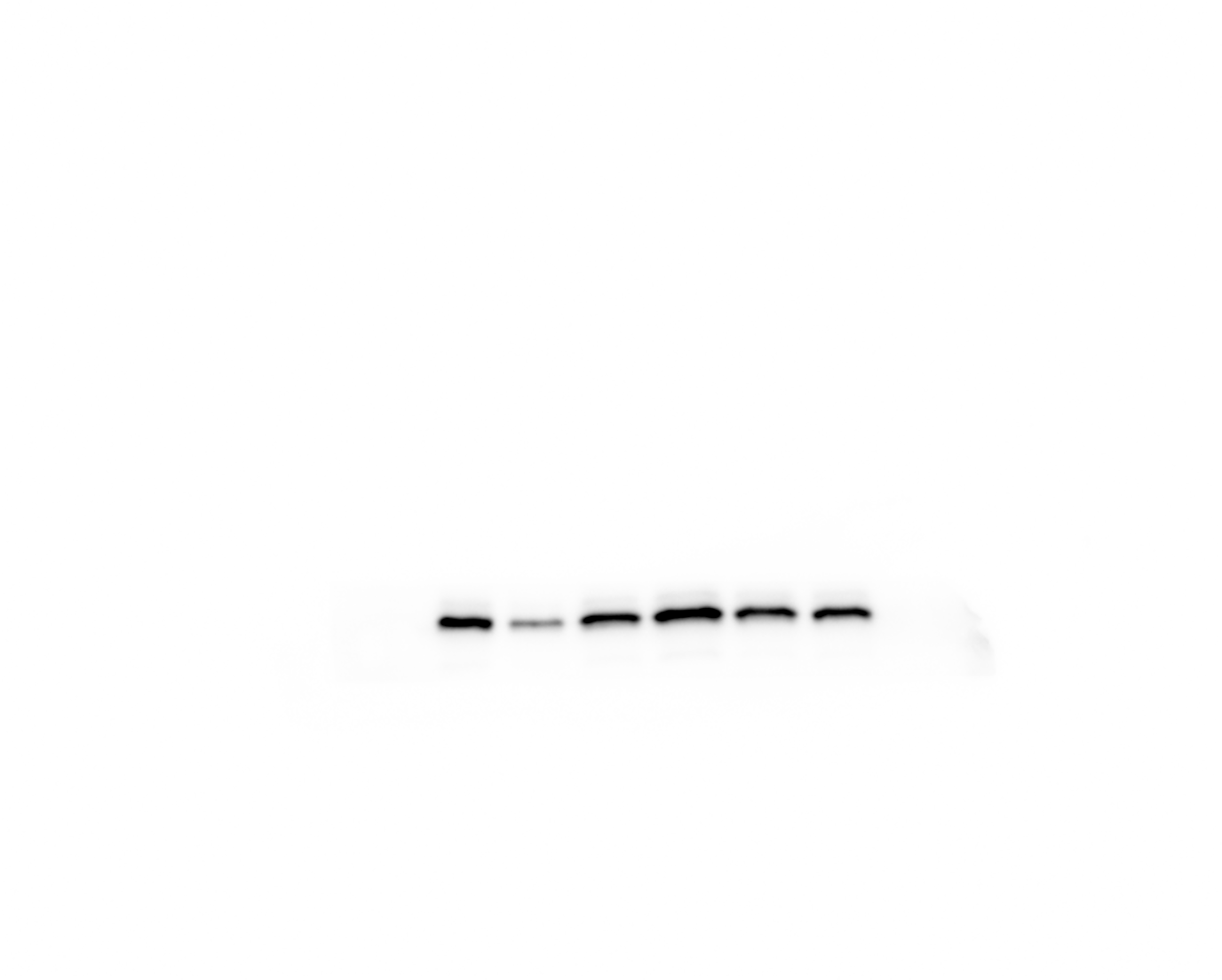


**GCLM GPX4**


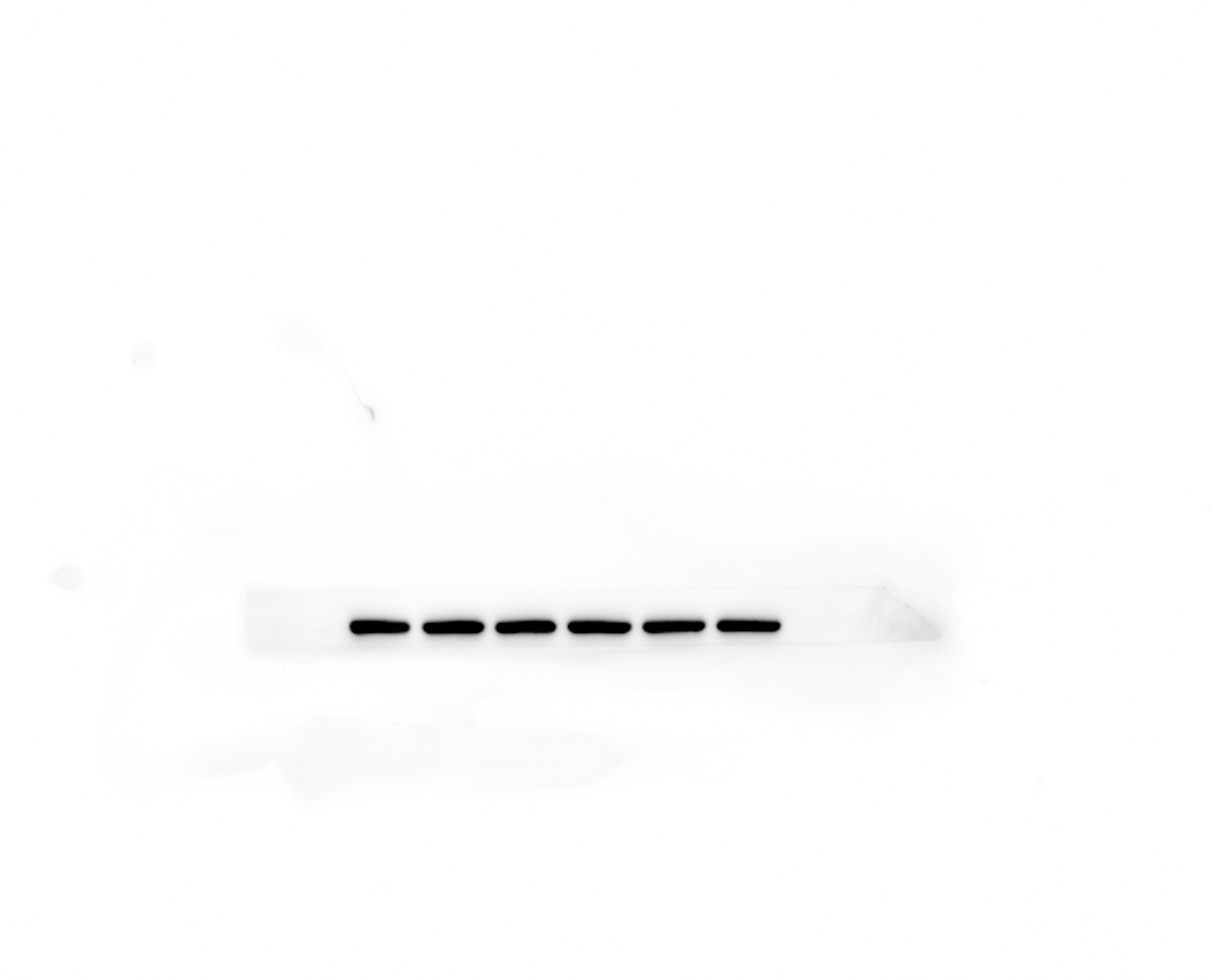


**β-actin**

**Fig 7**


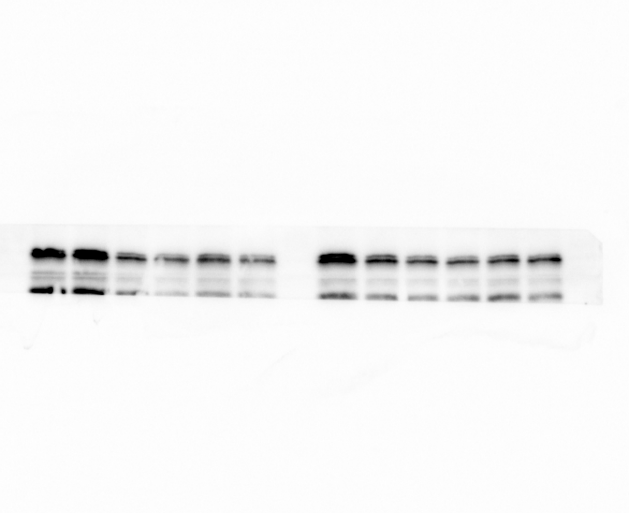

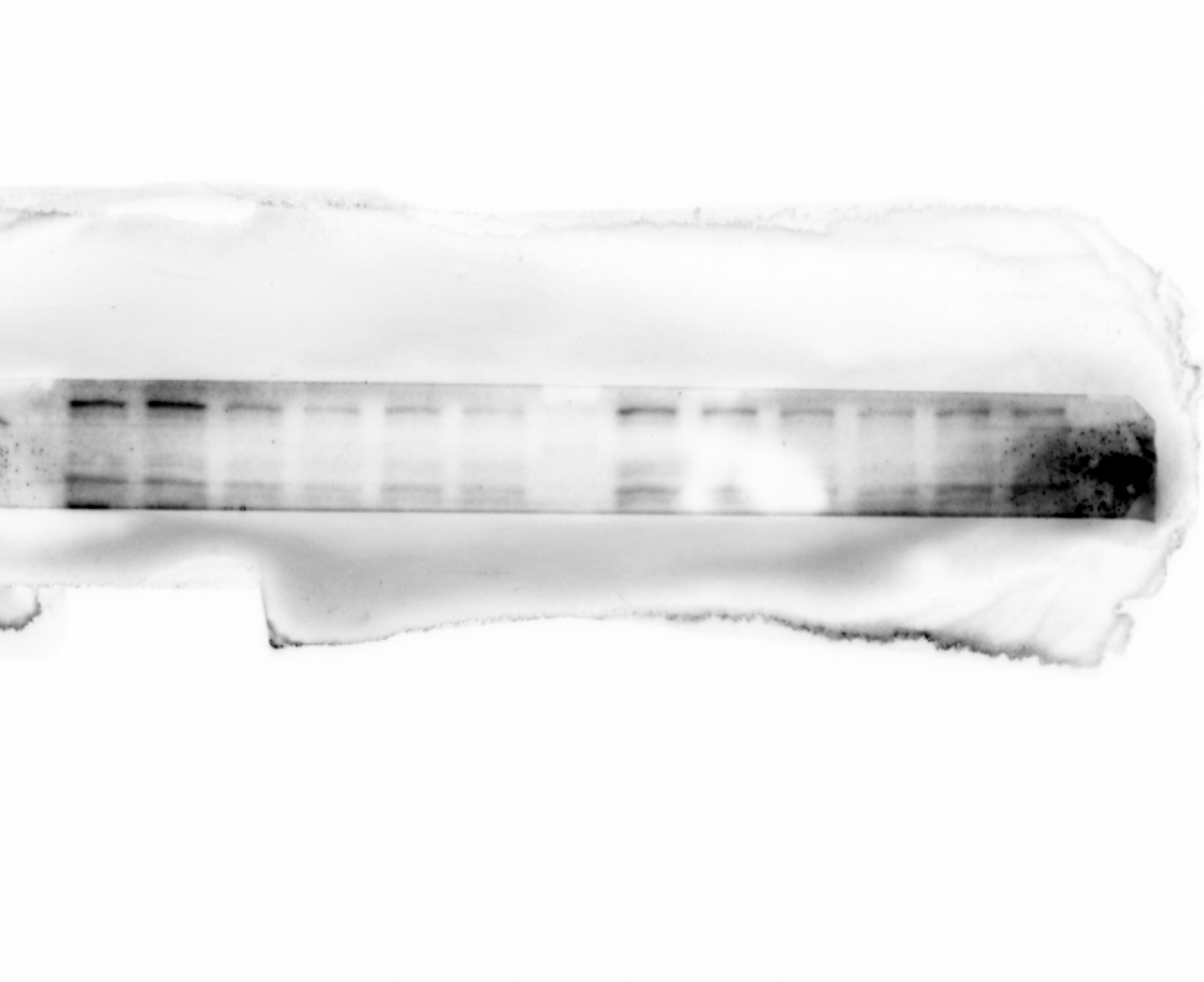


**SLC3A2 GCLM**


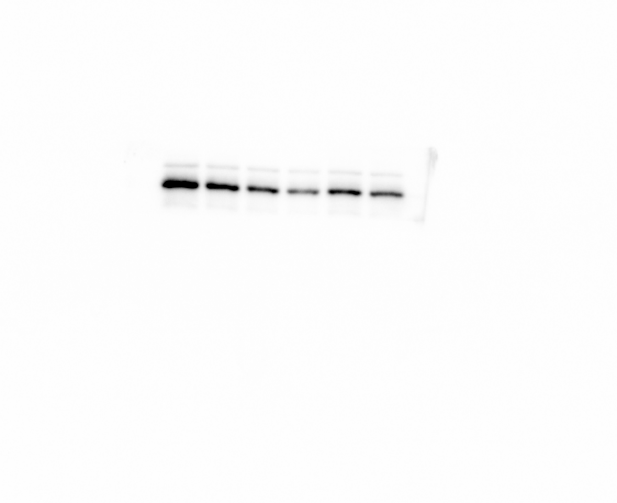

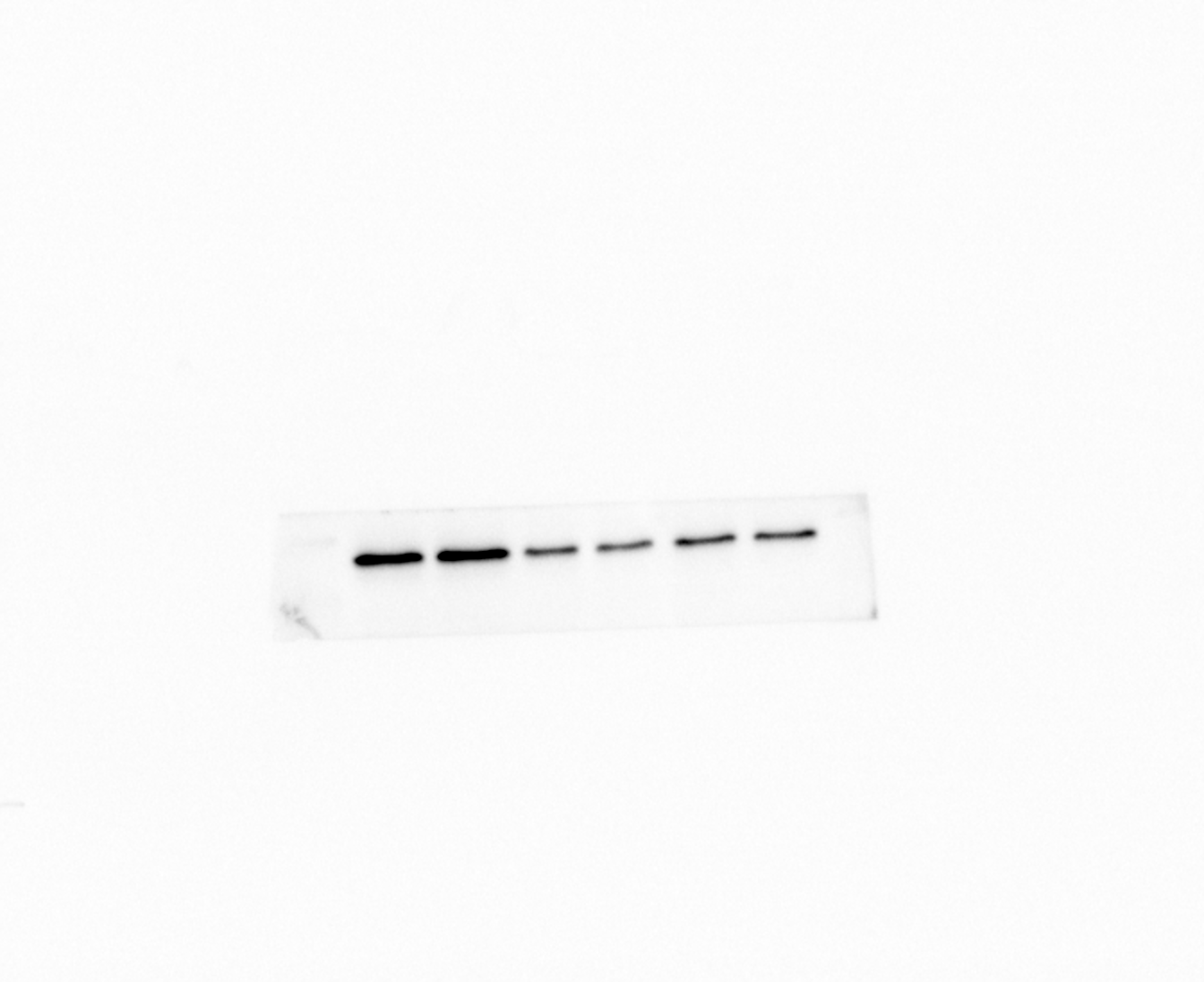


**GCLC GPX4**


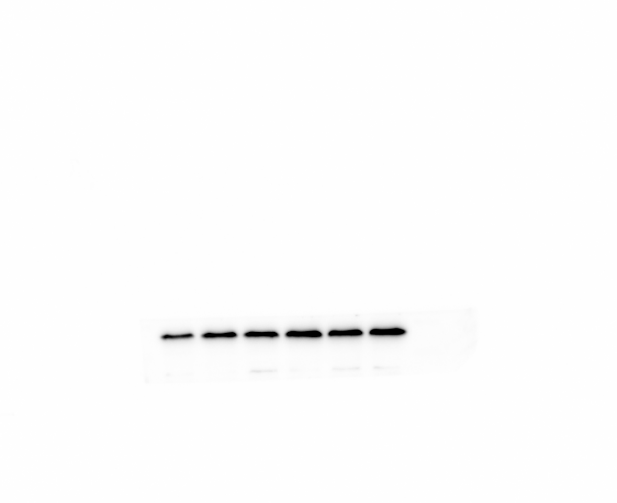

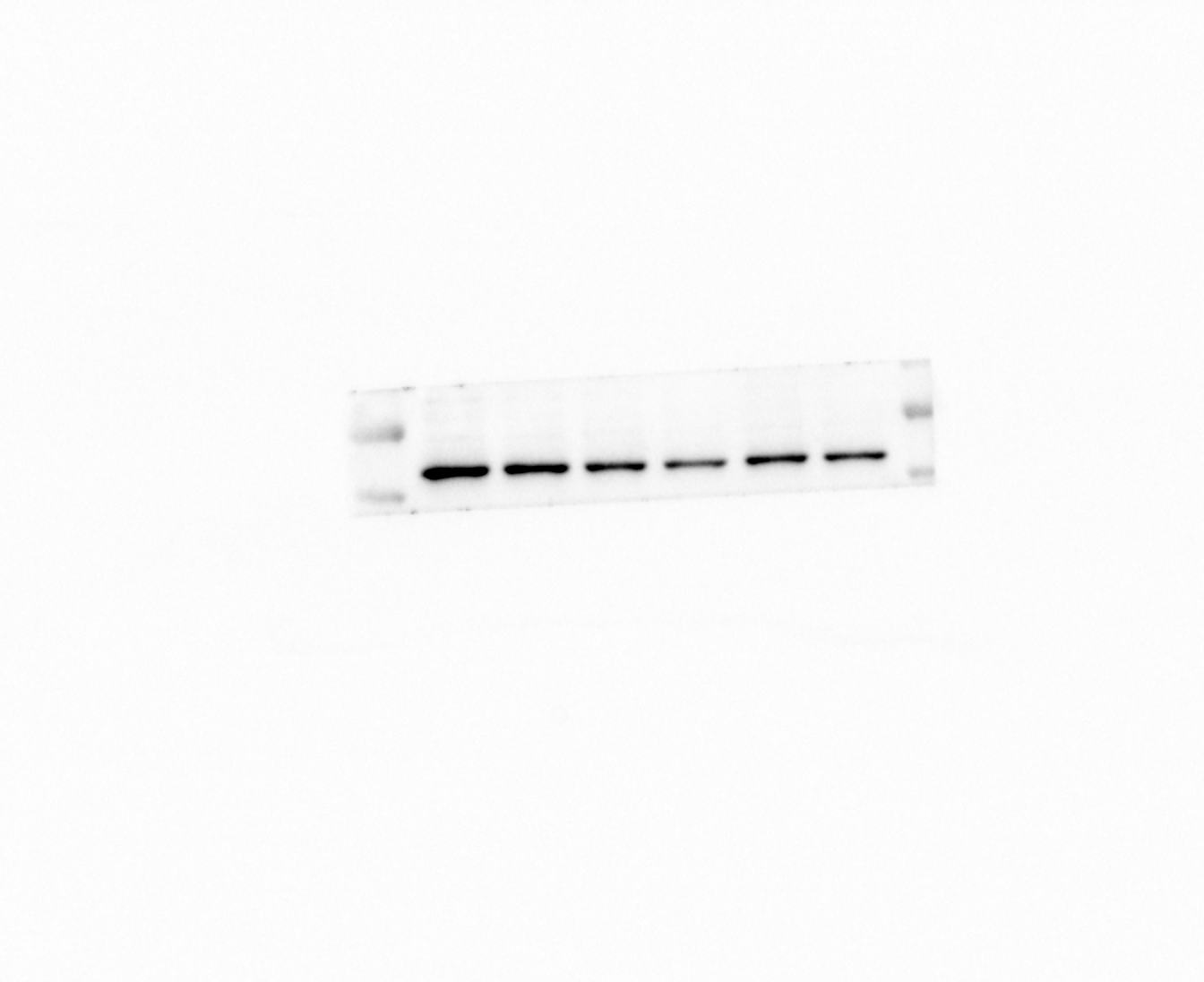


**MFN2 PINK1**


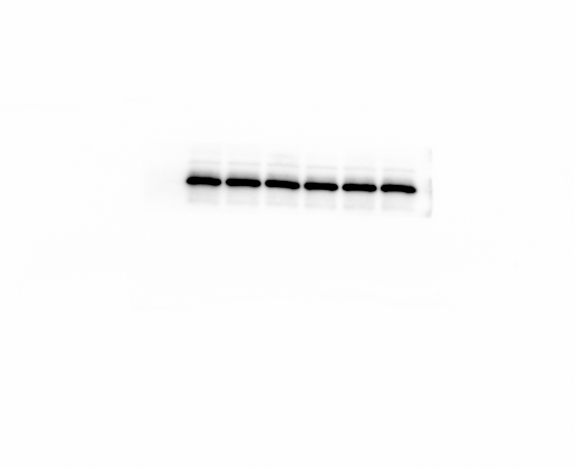

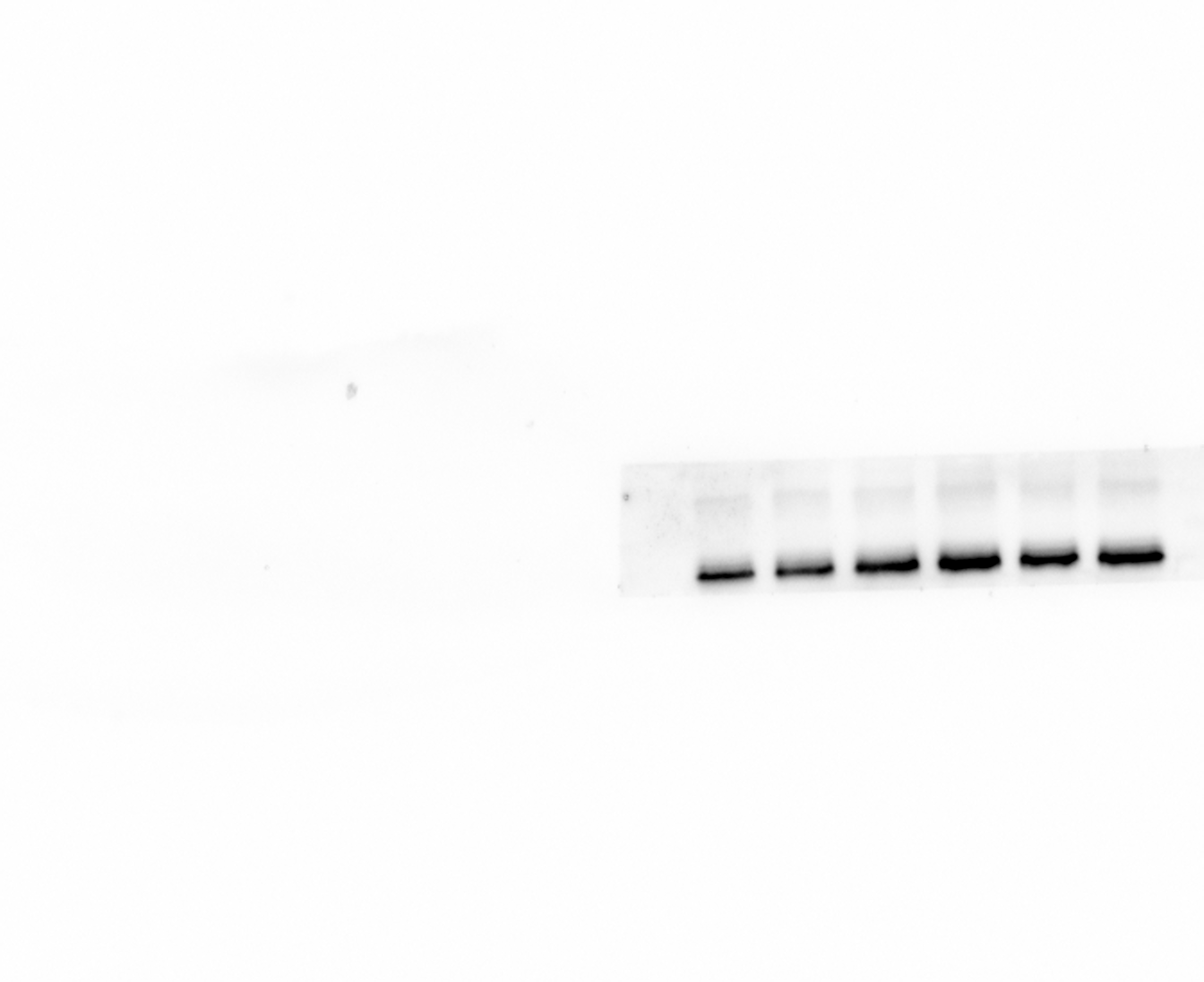


**Parkin β-actin**
